# Global, regional, and national burden of fracture of vertebral column, 1990–2021: analysis of data from the global burden of disease study 2021

**DOI:** 10.3389/fpubh.2025.1573888

**Published:** 2025-04-30

**Authors:** Yanni Lan, Shou Chen, Guipeng Lan, Cun Li, Jiyong Wei

**Affiliations:** ^1^Department of Pharmacy, The People’s Hospital of Guangxi Zhuang Autonomous Region & Guangxi Academy of Medical Sciences, Nanning, China; ^2^Departments of Spine Orthopedics, The Fourth Affiliated Hospital of Guangxi Medical University, Liuzhou, China; ^3^Department of Bone Surgery, The Eight People’s Hospital of Nanning, Nanning, China; ^4^Department of Orthopedic Surgery, The First People’s Hospital of Nanning, The Fifth Affiliated Hospital of Guangxi Medical University, Nanning, China

**Keywords:** fracture of vertebral column, global burden of disease, incidence, prevalence, years lived with disability

## Abstract

**Background:**

Fractures of the vertebral column, encompassing various spinal injuries, represent a significant public health burden worldwide. These injuries can lead to long-term disability, reduced quality of life, and substantial healthcare costs.

**Methods:**

We utilized comprehensive data sources from the Global Health Data Exchange (GHDx). The study employed the incidence, prevalence, and years lived with disability (YLDs) metric to quantify the burden. First, numbers and age-standardized rates (ASRs) of incidence, prevalence, and YLDs were assessed globally and by sub-types including sex, age, socio-demographic index (SDI) regions, Global Burden of Disease Study (GBD) regions, and countries in 2021. Furthermore, the temporal trend of the disease burden was explored by the linear regression model from 1990 to 2021. The cluster analysis was used to evaluate the changing pattern of related disease burden across GBD regions. Finally, the age-period-cohort (APC) model were used to predict the future disease burden in the next 25 years.

**Results:**

Exposure to fracture of vertebral column contributed to 7,497,446 incidence, 5,371,438 prevalence, and 545,923 YLDs globally in 2021. The disease burden was higher in males than in females. And it was higher in older adults. High SDI regions were high-risk areas. From 1990 to 2021, the number of cases showed the increasing trend, and the ASRs showed the decreasing trend. The predicted results showed that the number of cases for both genders would still increase from 2022 to 2046.

**Conclusion:**

Our findings highlight the substantial and growing global burden of vertebral column fractures, with significant variations across regions and countries. Targeted interventions to address modifiable risk factors, such as osteoporosis and falls, are essential to mitigate the burden.

## Introduction

Fractures of the vertebral column, which encompass injuries to the spine including those to the cervical, thoracic, lumbar, and sacral regions, represent a significant and multifaceted health burden globally. These injuries can result in varying degrees of disability, chronic pain, reduced quality of life, and substantial economic costs (1, 2). Despite advancements in trauma care and rehabilitation, the incidence and associated morbidity of vertebral column fractures remain high, particularly in aging populations and in low- and middle-income countries (LMICs) (3, 4).

Previous studies have documented the significant impact of vertebral column fractures on individual and societal levels. For instance, a systematic review by Sing et al. (5) reported that spinal fractures are a major contributor to the global burden of osteoporosis-related fractures, with a substantial increase in incidence projected over the next few decades due to population aging. Similarly, a study by Murray et al. (6) in the Global Burden of Disease Study 2010 highlighted the substantial disability-adjusted life years (DALYs) lost due to spinal injuries, emphasizing the need for targeted interventions to reduce this burden.

Regional variations in the burden of vertebral column fractures have also been well-documented. In high-income countries (HICs), while the incidence of traumatic spinal injuries may be declining due to improved road safety and workplace regulations, the burden of fragility fractures, particularly in the older adult, continues to rise (7, 8). Conversely, in LMICs, the burden is often exacerbated by a combination of factors including limited access to healthcare, inadequate trauma care infrastructure, and high rates of road traffic accidents and falls (9, 10).

Moreover, recent literature has emphasized the role of modifiable risk factors in the etiology of vertebral column fractures. Osteoporosis, a major underlying condition, is increasingly recognized as a global public health issue, with a high prevalence across all age groups and in both genders (11). Other risk factors such as smoking, alcohol consumption, and sedentary lifestyles have also been implicated in the development of fragility fractures (12, 13).

Despite these advancements in understanding the burden and etiology of vertebral column fractures, there remains a significant gap in the comprehensive assessment of global, regional, and national trends over extended periods. The Global Burden of Disease Study (GBD) provides a unique opportunity to fill this gap by leveraging a comprehensive and standardized approach to estimate the burden of diseases, injuries, and risk factors across different populations and time periods (14). The latest iteration of the GBD, the GBD 2021, includes updated data sources, methodologies, and risk factor estimates, providing a robust platform for analyzing trends in the burden of vertebral column fractures.

In this study, we analyze the global, regional, and national burden of vertebral column fractures from 1990 to 2021 using data from the GBD 2021. By examining trends in incidence, prevalence, and years lived with disability (YLDs), we aim to provide a comprehensive assessment of the burden of vertebral column fractures and identify key areas for intervention. Through this analysis, we hypothesize that our findings may reveal evolving epidemiological patterns of vertebral column fractures and could inform the prioritization of future research and policy development.

## Methods

### Study design and data sources

This study employed a comprehensive, retrospective analysis to estimate the global, regional, and national burden of vertebral column fractures from 1990 to 2021. Data were sourced from the GBD Study 2021, a collaborative effort involving hundreds of researchers worldwide to quantify health loss due to diseases, injuries, and risk factors. The GBD database integrates various data streams, including population-based surveys, hospital records, vital registration systems, and published literature, ensuring a robust and comprehensive dataset for analysis (15, 16).

### Case definition and classification

Vertebral column fractures, encompassing fractures of the cervical, thoracic, and lumbar spine, were defined according to the International Classification of Diseases (ICD) codes. Specifically, ICD-9 codes 805.0–806.9 and ICD-10 codes S12.0-S12.9, S22.0-S22.1, and S32.0 were utilized to identify cases. Fractures were further classified as traumatic or non-traumatic based on the underlying cause, with traumatic fractures attributed to external forces such as falls, motor vehicle accidents, or violence, and non-traumatic fractures often associated with underlying medical conditions like osteoporosis (17).

### Estimation procedures

The burden of vertebral column fractures was assessed using incidence, prevalence, and YLDs. Incidence and prevalence were estimated by age, sex, location, and year using DisMod-MR 2.1, a Bayesian meta-regression tool developed by the Institute for Health Metrics and Evaluation (IHME) (18). DisMod-MR leverages available data to produce internally consistent, cause-specific estimates by fitting a series of mathematical models to the data.

YLDs were calculated by multiplying the prevalence of vertebral column fractures by the disability weights derived from population-based surveys. Disability weights reflect the severity of health loss associated with specific conditions on a scale from 0 (perfect health) to 1 (death). For vertebral column fractures, disability weights varied based on the type and severity of the injury, as well as the duration of recovery (16).

### Uncertainty analysis

To account for uncertainty in the estimates, 95% uncertainty intervals (UIs) were calculated for all metrics. These intervals reflect the precision of the estimates, with narrower intervals indicating greater certainty. Uncertainty was incorporated into the DisMod-MR model through the use of prior distributions and likelihood functions, which allowed for the propagation of uncertainty from input data to final estimates (18).

### Geographical

The GBD 2021 study categorizes 204 countries and territories into five socio-demographic index (SDI) quintiles—low, low-middle, middle, high-middle, and high SDI regions—based on a composite metric of development status. The SDI is calculated as the geometric mean of three normalized indicators: (1) per capita income (lag-distributed income per capita), (2) average educational attainment (years of schooling for individuals aged 15+), and (3) total fertility rate under age 25 (16). These SDI groups are not geographically contiguous but instead reflect comparable levels of socioeconomic development. For instance, high SDI regions include countries such as the United States and Japan, while low SDI regions encompass nations like Niger and Somalia. This stratification enables the identification of burden patterns tied to development disparities rather than geographic proximity alone. A full list of SDI classifications by country is available in the GBD 2021 methodology report.

### Statistical analysis

First, the number of incidence, prevalence, and YLDs of fracture of vertebral column and their corresponding ASRs were reported in 2021 globally and by different sub-types including sex, age, SDI regions, GBD regions, and countries. Second, the temporal trend of the disease burden was explored globally and by sub-types from 1990 to 2021. The estimated annual percentage change (EAPC) value was estimated by linear regression model. Based on the EAPC values, the cluster analysis was used to evaluate the changing pattern of related disease burden across GBD regions. Finally, the future disease burden from 2020 to 2044 was predicted by using the age-period-cohort (APC) model under the maximum likelihood framework.

When the *p*-value was less than 0.05, it was considered statistically significant. The R (version 4.0.2) software was used for database construction, collation, and analysis.

## Results

### The disease burden attributable to fracture of vertebral column in 2021

In 2021, the number of fracture of vertebral column-related incidence was 7,497,446 [95% uncertainty intervals (UI): 5,834,963–9,737,255]. The corresponding age-standardized incidence rate (ASIR) was 92.75 (95% UI: 72.12–119.99) per 100,000 population. The number of fracture of vertebral column-related prevalence was 5,371,438 (95% UI: 4,703,837–6,196,132) in 2021. The corresponding age-standardized prevalence rate (ASPR) was 65.19 (95% UI: 56.89–75.28) per 100,000 population. The number of YLDs attributable to fracture of vertebral column was 545,923 (95% UI: 366,571–757,099). And the corresponding ASR of YLDs was 6.62 (95% UI: 4.43–9.20) per 100,000 population (Tables 1–3).

**Table 1:** The number of incidence cases and the age-standardized incidence rate attributable to fracture of vertebral column in 1990 and 2021, and its trends from 1990 to 2021 globally.

	Number of incidence cases (95% UI) in 1990	The age-standardized incidence rate/100,000 (95% UI) in 1990	Number of incidence cases (95% UI) in 2021	The age-standardized incidence rate/100,000 (95% UI) in 2021	EAPC (95% CI)
Global	5,856,226 (4,615,149–7,402,697)	115.75 (91.28–145.92)	7,497,446 (5,834,963–9,737,255)	92.75 (72.12–119.99)	−0.77 (−0.81 to −0.73)
Sex					
Female	2,303,829 (1,749,085–3,021,699)	92.47 (70.45–121.93)	3,213,761 (2,380,647–4,398,723)	76.86 (57.09–104.49)	−0.66 (−0.7 to −0.61)
Male	3,552,397 (2,848,067–4,417,084)	136.55 (110.03–168.79)	4,283,684 (3,389,625–5,420,126)	107.4 (85.22–135.88)	−0.82 (−0.86 to −0.78)
Age					
<5 years	414,335 (328,806–525,725)	66.83 (53.04–84.8)	250,243 (192,994–326,235)	38.02 (29.32–49.57)	−1.92 (−2.1 to −1.75)
5–9 years	412,227 (294,839–568,968)	70.64 (50.53–97.5)	333,401 (232,544–468,010)	48.53 (33.85–68.12)	−1.11 (−1.21 to −1.02)
10–14 years	414,202 (292,167–579,448)	77.32 (54.54–108.17)	377,252 (258,410–535,107)	56.59 (38.76–80.27)	−1.08 (−1.16 to −1)
15–19 years	587,581 (442,417–774,051)	113.12 (85.17–149.02)	507,985 (376,482–680,799)	81.41 (60.34–109.11)	−1.06 (−1.16 to −0.95)
20–24 years	622,879 (474,476–819,615)	126.58 (96.42–166.56)	556,476 (422,807–748,538)	93.19 (70.8–125.35)	−1.1 (−1.17 to −1.02)
25–29 years	534,266 (401,336–729,920)	120.71 (90.67–164.91)	530,275 (395,036–723,648)	90.13 (67.14–123)	−0.97 (−1.03 to −0.92)
30–34 years	466,921 (348,457–622,750)	121.15 (90.41–161.58)	550,438 (404,094–738,212)	91.06 (66.85–122.12)	−0.99 (−1.04 to −0.94)
35–39 years	418,378 (308,476–578,543)	118.77 (87.57–164.24)	520,849 (382,243–723,954)	92.87 (68.15–129.08)	−1 (−1.09 to −0.91)
40–44 years	330,194 (248,525–441,923)	115.26 (86.75–154.26)	447,752 (334,193–605,007)	89.51 (66.81–120.94)	−0.99 (−1.08 to −0.9)
45–49 years	261,940 (193,128–348,872)	112.81 (83.17–150.25)	432,721 (311,519–590,478)	91.39 (65.79–124.7)	−0.81 (−0.89 to −0.72)
50–54 years	256,384 (188,946–350,408)	120.61 (88.89–164.84)	444,512 (323,104–611,278)	99.91 (72.62–137.39)	−0.6 (−0.67 to −0.54)
55–59 years	227,968 (166,224–319,727)	123.09 (89.75–172.64)	434,337 (312,319–620,530)	109.76 (78.92–156.81)	−0.31 (−0.34 to −0.28)
60–64 years	208,072 (147,269–285,094)	129.55 (91.69–177.51)	398,273 (275,529–562,898)	124.44 (86.09–175.88)	−0.09 (−0.12 to −0.05)
65–69 years	178,074 (126,551–248,905)	144.06 (102.38–201.36)	381,804 (261,891–538,256)	138.41 (94.94–195.13)	−0.04 (−0.08 to −0.01)
70–74 years	148,911 (103,878–211,330)	175.89 (122.7–249.62)	357,208 (241,825–518,169)	173.54 (117.48–251.74)	−0.14 (−0.19 to −0.1)
75–79 years	154,241 (103,574–222,196)	250.57 (168.26–360.97)	317,703 (204,582–463,651)	240.89 (155.12–351.56)	−0.14 (−0.23 to −0.05)
80–84 years	124,235 (81,107–181,452)	351.19 (229.27–512.93)	306,193 (197,871–449,087)	349.6 (225.92–512.76)	−0.06 (−0.11 to −0.01)
85–89 years	68,581 (42,694–102,303)	453.85 (282.53–677.01)	215,413 (135,783–318,360)	471.14 (296.98–696.3)	0.07 (−0.02 to 0.16)
90–94 years	21,857 (13,212–32,969)	510.07 (308.31–769.36)	101,006 (61,788–149,055)	564.62 (345.39–833.2)	0.36 (0.25–0.47)
95+ years	4,978 (2,859–8,093)	488.92 (280.82–794.93)	33,605 (19,361–52,897)	616.58 (355.22–970.53)	0.67 (0.5–0.84)
SDI region					
High-middle SDI	1,445,832 (1,124,134–1,834,572)	135.4 (105.36–171.8)	1,531,877 (1,165,619–2,014,820)	109.16 (83.64–142.31)	−0.89 (−0.96 to −0.81)
High SDI	1,769,308 (1,367,209–2,274,778)	190.52 (147.68–245.09)	2,141,941 (1,580,376–2,884,480)	157.17 (119.04–207.76)	−0.68 (−0.7 to −0.65)
Low-middle SDI	864,955 (682,062–1,107,951)	80.93 (64.61–103.72)	1,265,742 (996,257–1,631,788)	69.74 (54.83–90.51)	−0.62 (−0.73 to −0.5)
Low SDI	358,350 (273,884–476,730)	74.64 (58.14–97.29)	640,628 (493,996–832,902)	63.4 (49.74–80.95)	−0.41 (−0.63 to −0.18)
Middle SDI	1,410,783 (1,113,366–1,791,737)	83.76 (66.15–105.83)	1,910,369 (1,474,209–2,500,467)	76.23 (58.96–99.58)	−0.23 (−0.3 to −0.16)
GBD region					
Advanced Health System	2,712,766 (2,105,454–3,436,731)	197.47 (153.61–250.88)	2,892,815 (2,176,530–3,860,517)	158.98 (120.76–207.92)	−0.82 (−0.88 to −0.77)
Africa	451,080 (348,649–593,543)	72.04 (56.54–92.01)	658,959 (533,739–814,927)	51.82 (41.8–64.43)	−1.01 (−1.18 to −0.84)
African Region	335,643 (254,486–452,470)	66.61 (51.4–86.19)	498,296 (399,890–621,975)	47.23 (37.97–58.78)	−1.12 (−1.36 to −0.88)
America	1,079,154 (847,343–1,352,306)	152.51 (120.04–191.7)	1,423,890 (1,104,790–1,839,031)	127.21 (98.52–161.67)	−0.47 (−0.54 to −0.4)
Andean Latin America	33,496 (26,602–42,689)	86.87 (69.52–109.34)	48,851 (38,490–62,399)	73.08 (57.51–93.44)	−0.38 (−0.48 to −0.28)
Asia	2,577,738 (2,027,202–3,290,937)	86 (67.7–109.3)	3,813,335 (2,957,627–4,993,738)	80.46 (62.38–105.45)	−0.29 (−0.37 to −0.22)
Australasia	52,676 (41,245–66,800)	256.41 (200.62–325.05)	82,214 (60,157–109,852)	232.18 (174.33–303.58)	−0.22 (−0.31 to −0.13)
Basic Health System	1,922,336 (1,518,345–2,433,428)	84.52 (66.57–106.97)	2,544,522 (1,966,022–3,316,196)	78.5 (60.71–102.16)	−0.17 (−0.24 to −0.1)
Caribbean	25,557 (20,513–31,965)	73.69 (59.04–92.42)	41,527 (33,084–53,030)	85.96 (68.43–109.85)	0.43 (−0.33 to 1.18)
Central Africa	29,711 (24,043–37,040)	47.13 (38.13–57.98)	74,722 (59,694–93,659)	48.58 (38.81–59.65)	−1.05 (−1.78 to −0.31)
Central Asia	79,705 (63,132–101,605)	112.43 (89–143.51)	86,933 (68,675–113,052)	90.11 (71.1–117.03)	−0.9 (−1.1 to −0.71)
Central Europe	273,079 (209,969–362,160)	215.29 (165.39–285.84)	202,041 (153,511–270,779)	163.9 (123.54–221.11)	−1.06 (−1.13 to −0.99)
Central Latin America	214,246 (165,992–278,339)	130.24 (101.9–167.9)	226,974 (176,349–291,985)	89.37 (69.36–114.98)	−0.72 (−0.93 to −0.5)
Central Sub-Saharan Africa	28,715 (22,884–36,413)	53.51 (42.85–65.95)	60,232 (47,863–74,329)	47.93 (37.98–58.52)	−1.18 (−1.88 to −0.48)
Commonwealth High Income	211,556 (161,416–272,655)	180.09 (138.59–232.84)	293,529 (213,461–400,844)	164.43 (122.12–220.61)	−0.22 (−0.32 to −0.11)
Commonwealth Low Income	87,682 (68,689–111,550)	42.13 (33.56–52.94)	144,805 (111,713–186,225)	38.33 (30.09–49.26)	−1.06 (−1.72 to −0.38)
Commonwealth Middle Income	870,840 (679,649–1,131,889)	83.67 (65.47–109.88)	1,435,668 (1,109,918–1,887,765)	74.61 (57.23–98.8)	−0.42 (−0.5 to −0.35)
East Asia	811,283 (619,339–1,061,744)	68.21 (52.45–88.53)	1,219,234 (908,987–1,625,137)	75.19 (56.5–100.88)	−0.03 (−0.35 to 0.29)
East Asia & Pacific—WB	1,507,503 (1,173,874–1,914,318)	83.77 (65.43–106.75)	2,016,775 (1,545,776–2,658,828)	77.89 (60.21–102.73)	−0.45 (−0.61 to −0.29)
Eastern Africa	172,077 (114,097–276,745)	94.56 (65.1–147.64)	176,930 (138,546–227,821)	49.5 (38.94–62.34)	−1.44 (−1.9 to −0.98)
Eastern Europe	481,008 (372,151–626,966)	210.83 (163.25–274.93)	355,895 (275,645–470,575)	168.73 (130.9–221.27)	−1.05 (−1.33 to −0.77)
Eastern Mediterranean Region	337,138 (272,072–412,262)	91.24 (74.68–110.79)	629,557 (498,179–798,500)	84.23 (66.86–106.18)	0.31 (0.12–0.51)
Eastern Sub-Saharan Africa	165,425 (110,510–263,433)	83.97 (57.93–130.52)	178,286 (139,448–228,457)	46.21 (36.44–58.42)	−1.5 (−1.98 to −1.01)
Europe	1,738,291 (1,334,716–2,220,054)	208.26 (160.77–266.46)	1,591,133 (1,190,020–2,142,572)	162.97 (122.97–213.71)	−0.93 (−1.04 to −0.82)
Europe & Central Asia—WB	1,792,667 (1,376,776–2,287,610)	203.55 (157.38–259.99)	1,654,728 (1,244,837–2,221,707)	157.68 (119.42–206.14)	−0.97 (−1.08 to −0.87)
European Region	1,804,330 (1,385,802–2,302,313)	203.24 (157.2–259.6)	1,672,764 (1,258,842–2,245,435)	157.62 (119.35–206)	−0.97 (−1.08 to −0.86)
High-income Asia Pacific	343,178 (266,655–439,410)	191.02 (148.76–244.2)	304,508 (226,180–407,850)	130.33 (99.13–170.3)	−1.42 (−1.53 to −1.32)
High-income North America	527,853 (411,566–663,516)	177.24 (137.54–223.05)	757,555 (564,304–997,819)	158.96 (122.41–206.63)	−0.41 (−0.52 to −0.3)
Latin America & Caribbean—WB	554,241 (434,319–711,555)	127.53 (100.23–162.59)	669,535 (524,127–856,355)	99.86 (77.96–127.64)	−0.57 (−0.73 to −0.42)
Limited Health System	1,132,243 (883,323–1,439,600)	80.1 (62.69–104.43)	1,842,514 (1,438,098–2,402,459)	70.63 (54.96–93.32)	−0.47 (−0.57 to −0.37)
Middle East & North Africa—WB	286,707 (229,395–350,777)	113.15 (91–138.06)	474,231 (375,363–596,346)	99.37 (79.35–124.87)	0.2 (0–0.4)
Minimal Health System	81,883 (62,301–111,066)	60.74 (47.14–79.8)	210,707 (155,248–293,178)	64.28 (48.75–86.26)	−0.31 (−0.78 to 0.16)
North Africa and Middle East	353,175 (284,333–433,155)	104.06 (84.64–127.44)	617,361 (486,189–785,817)	98.91 (77.9–125.93)	0.31 (0.12–0.49)
North America	527,756 (411,494–663,387)	177.21 (137.52–223.01)	757,460 (564,241–997,685)	158.94 (122.39–206.6)	−0.41 (−0.52 to −0.3)
Northern Africa	111,690 (88,681–141,121)	92.37 (73.38–116.51)	153,398 (121,832–192,972)	74.69 (59.35–93.47)	−0.66 (−0.73 to −0.59)
Oceania	3,067 (2,410–3,890)	51.91 (40.94–65.99)	7,692 (5,963–9,984)	61 (46.98–79.4)	0.12 (−0.36 to 0.59)
Region of the Americas	1,079,154 (847,343–1,352,306)	152.51 (120.04–191.7)	1,423,890 (1,104,790–1,839,031)	127.21 (98.52–161.67)	−0.47 (−0.54 to −0.4)
South-East Asia Region	984,593 (769,887–1,278,062)	84.07 (66.11–109.48)	1,495,107 (1,150,029–1,986,053)	74.35 (57.01–98.7)	−0.57 (−0.7 to −0.44)
South Asia	798,849 (621,332–1,050,617)	83.33 (65.19–110.14)	1,317,848 (1,008,026–1,757,072)	75.77 (57.31–101.33)	−0.45 (−0.56 to −0.34)
South Asia—WB	837,150 (652,990–1,095,008)	84.48 (65.96–111.28)	1,408,008 (1,089,438–1,877,428)	78.38 (60.68–104.76)	−0.4 (−0.51 to −0.29)
Southeast Asia	324,576 (261,725–403,165)	72.45 (58.16–90.58)	418,745 (331,267–531,579)	60.26 (47.65–76.78)	−0.61 (−0.85 to −0.36)
Southern Africa	59,852 (47,626–75,073)	65.87 (52.54–80.64)	81,827 (65,879–101,856)	47.51 (38.36–58.7)	−1.25 (−1.41 to −1.08)
Southern Latin America	75,422 (59,443–96,533)	153.26 (120.76–196.29)	104,148 (80,434–133,864)	149.29 (115.32–191.82)	−0.06 (−0.18 to 0.05)
Southern Sub-Saharan Africa	36,118 (28,571–45,630)	71.74 (56.47–91.52)	42,218 (33,535–52,630)	52.06 (41.43–65.02)	−1.2 (−1.33 to −1.08)
Sub-Saharan Africa—WB	340,101 (254,879–468,590)	66.94 (51.06–88.31)	506,748 (407,011–634,872)	47.16 (37.88–58.6)	−1.06 (−1.29 to −0.83)
Tropical Latin America	206,425 (156,537–274,453)	135.6 (103.86–181.36)	249,486 (191,127–326,516)	106.31 (81.32–139.24)	−0.73 (−0.86 to −0.59)
Western Africa	77,750 (62,724–97,123)	47.1 (38.02–59.52)	172,082 (136,556–215,531)	44.29 (35.4–55.8)	−0.28 (−0.4 to −0.15)
Western Europe	936,244 (700,241–1,249,608)	221.86 (167.7–294.33)	979,713 (696,247–1,351,596)	177.62 (128.55–243.91)	−0.76 (−0.82 to −0.7)
Western Pacific Region	1,288,735 (1,003,185–1,653,520)	86.45 (67.79–111.12)	1,755,048 (1,332,198–2,331,673)	82.2 (62.75–109.1)	−0.38 (−0.57 to −0.2)
Western Sub-Saharan Africa	86,128 (69,368–107,384)	47.18 (38.12–59.47)	195,986 (156,081–244,567)	44.76 (35.81–56.18)	−0.23 (−0.34 to −0.12)
World Bank High Income	2,179,386 (1,680,785–2,803,472)	200.46 (155.3–257.41)	2,471,880 (1,825,152–3,335,018)	162.34 (122.42–214.67)	−0.75 (−0.77 to −0.73)
World Bank Low Income	254,763 (179,675–378,052)	80.52 (58.7–115.76)	417,453 (311,870–571,169)	61.8 (47.4–81.38)	−0.36 (−0.76 to 0.05)
World Bank Lower Middle Income	1,527,947 (1,206,697–1,959,163)	83.24 (66.11–106.26)	2,258,379 (1,757,437–2,929,479)	70.25 (54.68–91.59)	−0.65 (−0.74 to −0.56)
World Bank Upper Middle Income	1,887,120 (1,473,922–2,419,377)	94.83 (74.33–121.41)	2,342,834 (1,802,662–3,081,468)	88 (67.67–115.18)	−0.32 (−0.4 to −0.25)
Country					
Afghanistan	9,881 (7,622–13,252)	98.58 (77.27–128.16)	71,912 (39,609–135,127)	213.43 (120.65–399.19)	1.57 (0.68–2.46)
Albania	6,220 (4,534–8,683)	182.98 (133.33–255.43)	4,093 (3,065–5,615)	158.04 (119.49–214.26)	−0.89 (−1.2 to −0.59)
Algeria	26,191 (20,593–33,268)	103.44 (81.39–131.38)	33,424 (26,014–42,502)	76.4 (59.65–97.35)	−1.36 (−1.54 to −1.19)
American Samoa	25 (19–32)	55.13 (43.37–71.51)	26 (20–34)	52.84 (41.37–69.61)	0 (−0.76 to 0.76)
Andorra	142 (104–193)	254.54 (185.81–347.7)	287 (201–396)	264.3 (189.72–367.12)	0.11 (0.06–0.17)
Angola	8,766 (5,932–13,579)	82.23 (57.44–122.05)	12,716 (10,049–15,775)	44.18 (34.83–54.88)	−2.61 (−3.6 to −1.62)
Antigua and Barbuda	43 (32–59)	69.44 (51.78–96.09)	66 (48–93)	73.58 (54.22–103.01)	−0.02 (−0.39 to 0.35)
Argentina	48,502 (38,733–62,319)	147.72 (117.87–189.66)	63,783 (50,216–81,438)	137.23 (108.21–175.44)	−0.24 (−0.38 to −0.1)
Armenia	4,684 (3,687–6,020)	134.69 (105.96–172.87)	2,228 (1,736–2,863)	75.52 (59.11–97.05)	−1.38 (−2.25 to −0.5)
Australia	42,056 (32,967–53,307)	246.9 (192.71–313.47)	68,218 (49,834–92,140)	227.85 (170.01–298.4)	−0.14 (−0.23 to −0.06)
Austria	23,520 (17,501–32,027)	273.26 (205.12–368.07)	22,033 (15,519–30,458)	199.19 (143.6–275.11)	−0.93 (−1 to −0.86)
Azerbaijan	6,919 (5,496–8,831)	90.26 (71.45–115.35)	7,801 (6,106–10,142)	74.02 (57.93–95.74)	−0.86 (−1.47 to −0.25)
Bahamas	153 (122–192)	58.97 (47.5–74.08)	238 (190–301)	60.84 (48.4–76.79)	0.46 (0.01–0.91)
Bahrain	363 (286–461)	69.34 (55.07–87.11)	1,038 (823–1,351)	65.18 (51.67–84.34)	−0.39 (−0.52 to −0.26)
Bangladesh	40,078 (30,883–52,840)	36.1 (28.27–45.92)	61,624 (46,731–81,467)	36.89 (28.08–48.53)	−0.63 (−1.61 to 0.37)
Barbados	133 (105–171)	51.24 (40.49–66.24)	162 (125–211)	53.56 (41.5–69.69)	−0.05 (−0.2 to 0.1)
Belarus	18,973 (14,699–24,345)	181.13 (140.71–231.32)	17,033 (12,767–22,658)	176.66 (131.8–235.67)	−0.19 (−0.5 to 0.13)
Belgium	26,901 (19,927–36,477)	247.03 (185.77–331.49)	34,976 (24,573–48,727)	233.36 (167.6–325.89)	−0.04 (−0.33 to 0.25)
Belize	131 (102–170)	67.12 (53.19–86.66)	329 (259–430)	74.79 (59.22–96.93)	0.14 (−0.05 to 0.33)
Benin	2,052 (1,647–2,562)	45.06 (36.36–56.17)	5,338 (4,217–6,754)	44.44 (35.61–55.99)	−0.12 (−0.17 to −0.07)
Bermuda	36 (28–46)	59.85 (47.11–76.51)	40 (31–52)	59.29 (45.53–77.88)	−0.05 (−0.16 to 0.05)
Bhutan	298 (232–384)	52.1 (40.98–66.66)	503 (380–659)	69.73 (52.48–91.64)	0.68 (0.27–1.08)
Bolivia (Plurinational State of)	4,827 (3,866–6,069)	78.7 (63.17–98.72)	8,142 (6,432–10,410)	70.27 (55.61–89.99)	−0.43 (−0.46 to −0.39)
Bosnia and Herzegovina	8,761 (6,513–12,031)	191.74 (142.62–263.4)	4,477 (3,349–6,044)	138.72 (103.05–187.36)	−2.07 (−2.91 to −1.22)
Botswana	596 (476–739)	48.64 (39.09–60.33)	1,261 (1,009–1,572)	52.86 (42.52–66)	0.11 (−0.08 to 0.3)
Brazil	202,690 (153,650–269,574)	136.71 (104.71–182.93)	243,009 (186,163–318,135)	106.82 (81.64–140.03)	−0.74 (−0.87 to −0.6)
Brunei Darussalam	504 (401–633)	203.55 (163.02–254.05)	728 (566–910)	159.78 (124.2–200.19)	−0.79 (−0.84 to −0.74)
Bulgaria	18,104 (14,103–23,862)	213.99 (166.28–282.5)	11,200 (8,327–15,237)	173.99 (129.48–234.04)	−0.73 (−0.76 to −0.71)
Burkina Faso	4,079 (3,260–5,153)	47.08 (38.01–58.74)	11,338 (8,820–14,537)	54.24 (42.93–67.92)	0.33 (0.14–0.52)
Burundi	2,341 (1,881–2,948)	46.18 (37.24–58.3)	4,977 (3,979–6,266)	41.78 (33.85–51.83)	−4.59 (−6.98 to −2.14)
Cabo Verde	159 (124–202)	46.65 (36.96–58.76)	269 (210–348)	49.31 (38.72–63.11)	0.24 (0.19–0.28)
Cambodia	6,931 (5,504–9,004)	71.42 (57.52–89.68)	11,490 (8,752–15,211)	71.6 (54.3–94.75)	−0.09 (−0.35 to 0.18)
Cameroon	4,011 (3,200–5,052)	43.24 (34.79–54.52)	13,746 (10,993–17,309)	48.21 (38.53–60.07)	0.46 (0.34–0.58)
Canada	48,562 (38,082–62,274)	166.63 (131.09–212.18)	77,853 (56,233–105,007)	147.13 (108.96–197.59)	−0.28 (−0.33 to −0.22)
Central African Republic	1,247 (1,005–1,548)	49.17 (40.03–60.12)	4,322 (3,056–6,367)	76.09 (55.81–106.98)	1.52 (0.81–2.24)
Chad	3,350 (2,518–4,672)	55.27 (42.09–73.8)	8,353 (6,598–10,842)	50.31 (40.33–63.19)	−0.31 (−0.79 to 0.17)
Chile	21,316 (16,534–27,303)	163.05 (126.6–207.99)	34,409 (25,557–45,299)	175.43 (130.14–231.92)	0.37 (0.26–0.48)
China	784,386 (598,122–1,027,222)	68.32 (52.49–88.79)	1,194,465 (888,994–1,594,120)	76.38 (57.33–102.58)	0.02 (−0.31 to 0.34)
Colombia	40,641 (31,807–52,472)	122.47 (96.41–157.34)	38,220 (30,214–48,571)	78.4 (61.85–100.44)	−1.62 (−1.72 to −1.52)
Comoros	189 (150–234)	44.59 (35.91–55.45)	284 (228–359)	40.99 (33.01–51.52)	−0.09 (−0.39 to 0.21)
Congo	1,026 (826–1,282)	47.71 (38.36–59.37)	2,016 (1,617–2,497)	41.45 (32.89–51.73)	−1.78 (−3.22 to −0.31)
Cook Islands	11 (8–14)	58.59 (45.56–74.92)	10 (7–13)	52.07 (39.36–68.84)	−0.31 (−1.02 to 0.41)
Costa Rica	2,693 (2,043–3,566)	91.42 (70.23–119.35)	3,957 (3,052–5,164)	83.15 (63.89–107.86)	−0.33 (−0.36 to −0.29)
Croatia	10,701 (8,278–13,904)	215.59 (166.96–280.03)	9,421 (6,902–12,879)	174.91 (130.29–237.57)	−1.13 (−1.44 to −0.83)
Cuba	9,577 (7,634–12,046)	86.84 (69.25–109.5)	13,396 (10,062–18,355)	97.22 (73.53–127.2)	0.28 (0.2–0.36)
Cyprus	1,765 (1,356–2,254)	229.3 (175.36–296.85)	2,919 (2,134–3,999)	196.09 (143.71–266.71)	−0.52 (−0.67 to −0.38)
Czechia	27,708 (20,905–36,556)	253.01 (191.61–337.19)	20,410 (15,255–27,524)	173.91 (130.08–233.31)	−1.24 (−1.4 to −1.07)
CÃ´te d'Ivoire	4,931 (3,911–6,238)	46.48 (37.5–58.53)	10,987 (8,684–13,934)	45.6 (36.35–57.64)	−0.14 (−0.33 to 0.06)
Democratic People's Republic of Korea	10,348 (8,138–12,942)	50.15 (39.34–62.45)	12,237 (9,711–15,304)	42.31 (33.66–52.38)	−0.45 (−0.53 to −0.38)
Democratic Republic of the Congo	17,029 (13,621–21,509)	46.58 (37.49–57.53)	39,888 (31,591–49,391)	48.03 (37.91–58.63)	−0.7 (−1.51 to 0.13)
Denmark	13,648 (9,845–18,583)	221.53 (164.71–300.99)	11,178 (7,910–15,634)	157.55 (113.32–217.04)	−1.25 (−1.33 to −1.16)
Djibouti	242 (177–347)	58.05 (43.63–78.96)	485 (386–617)	42.88 (34.46–54.11)	−0.99 (−1.36 to −0.62)
Dominica	39 (31–49)	52.83 (42.18–66.17)	38 (30–48)	55.45 (44.14–70)	0.54 (0.03–1.05)
Dominican Republic	3,977 (3,179–5,044)	55.09 (44.16–68.53)	7,528 (5,968–9,449)	67.47 (53.53–84.6)	0.68 (0.48–0.88)
Ecuador	8,136 (6,471–10,322)	83.49 (66.85–105.9)	15,546 (12,311–19,630)	85.09 (67.48–107.28)	0.01 (−0.14 to 0.16)
Egypt	48,860 (38,445–61,449)	86.81 (68.81–109.59)	71,557 (56,710–90,554)	69.78 (55.46–87.95)	−0.56 (−0.64 to −0.47)
El Salvador	7,756 (5,590–11,197)	135.3 (99.91–189.1)	5,831 (4,566–7,477)	89.56 (70.23–114.91)	−0.65 (−0.95 to −0.34)
Equatorial Guinea	187 (150–238)	47.06 (38.01–58.17)	528 (419–665)	40.32 (31.97–50.81)	−0.52 (−0.58 to −0.46)
Eritrea	11,072 (4,809–22,558)	284.97 (131.13–586.75)	2,628 (2,106–3,295)	45.57 (36.93–57.39)	−2.65 (−4.32 to −0.96)
Estonia	3,623 (2,797–4,738)	228.02 (176.14–295.33)	1,836 (1,386–2,448)	137.85 (104.03–182.2)	−1.98 (−2.1 to −1.86)
Eswatini	388 (307–490)	52.73 (42.19–66.3)	722 (568–918)	62.07 (49.57–77.6)	0.22 (0.06–0.38)
Ethiopia	88,263 (48,667–163,902)	164.03 (97.03–293.3)	56,650 (39,693–87,095)	54.86 (40.61–77.54)	−2.17 (−3.07 to −1.26)
Fiji	300 (233–388)	40.8 (31.97–52.37)	345 (270–441)	38.37 (30.18–49.27)	−0.34 (−0.51 to −0.18)
Finland	15,774 (11,659–21,354)	289.17 (214.44–388.6)	17,282 (12,148–23,876)	241.64 (173.83–335.94)	−0.7 (−1.15 to −0.24)
France	173,167 (128,913–232,849)	268.89 (201.64–357.76)	190,225 (134,355–262,188)	217.73 (157.79–301.01)	−0.64 (−0.72 to −0.57)
Gabon	460 (367–573)	50.97 (41.03–63.6)	761 (608–960)	46.42 (36.89–58.42)	−0.36 (−0.39 to −0.33)
Gambia	343 (271–445)	38.63 (30.92–49.44)	819 (648–1,056)	40.3 (32.07–51.24)	0.06 (−0.04 to 0.15)
Georgia	7,527 (5,801–9,796)	136.66 (105.48–178.28)	5,118 (3,920–6,611)	147.3 (113.08–189.8)	0.23 (−0.13 to 0.59)
Germany	184,709 (137,496–250,634)	208.81 (156.95–279.32)	196,709 (138,098–270,623)	174.5 (126.25–238.22)	−0.66 (−0.7 to −0.61)
Ghana	5,412 (4,278–6,845)	40.35 (32.19–51.08)	12,767 (10,163–16,158)	42.01 (33.48–53.42)	0.08 (0.01–0.16)
Greece	24,119 (18,481–31,898)	222.88 (171.26–294.43)	16,853 (12,649–22,346)	156.07 (117.92–204.89)	−1.19 (−1.3 to −1.08)
Greenland	121 (93–157)	239.43 (181.29–318.23)	123 (89–167)	197.86 (145.24–267.26)	−0.71 (−0.8 to −0.62)
Grenada	55 (43–72)	64.34 (50.51–84.13)	73 (55–98)	70.22 (53.28–93.42)	0.24 (0.07–0.41)
Guam	64 (50–84)	48.11 (37.5–62.36)	74 (57–97)	45.16 (34.59–59.24)	−0.2 (−0.31 to −0.08)
Guatemala	10,320 (7,839–13,782)	121.24 (93.28–158.8)	16,779 (13,047–22,095)	106.7 (82.9–139.65)	−0.13 (−0.24 to −0.03)
Guinea	2,578 (2,071–3,224)	43.48 (35.02–54.24)	5,385 (4,264–6,737)	44.21 (35.7–55.37)	0.04 (−0.24 to 0.33)
Guinea-Bissau	498 (398–610)	53.75 (43.45–66.13)	850 (674–1,054)	48 (38.81–59.72)	−0.69 (−1.21 to −0.16)
Guyana	533 (430–662)	71.21 (57.5–88.3)	627 (504–785)	82.21 (65.73–103.07)	0.29 (0.12–0.47)
Haiti	4,524 (3,617–5,798)	71.79 (57.11–92.13)	11,264 (8,695–15,050)	86.39 (67.41–114.86)	0.21 (−1.4 to 1.84)
Honduras	4,272 (3,329–5,492)	87.13 (68.44–109.73)	7,756 (6,037–10,034)	78.11 (61.3–100.23)	−0.7 (−1.34 to −0.06)
Hungary	27,515 (20,501–37,112)	240.97 (180.67–317.99)	17,856 (13,231–24,226)	161.14 (120.69–214.72)	−1.59 (−1.72 to −1.47)
Iceland	533 (401–715)	203.46 (152.94–272.59)	655 (465–894)	169.65 (121.24–231.54)	−0.64 (−0.78 to −0.49)
India	700,538 (545,141–924,709)	94.31 (73.46–125.36)	1,141,146 (856,592–1,533,280)	84.52 (62.91–113.48)	−0.46 (−0.52 to −0.4)
Indonesia	115,737 (91,030–147,268)	66.25 (52.26–84.09)	136,104 (106,538–176,454)	50.68 (39.71–65.37)	−0.99 (−1.19 to −0.79)
Iran (Islamic Republic of)	78,884 (61,705–103,585)	138.05 (108.7–179.1)	71,074 (55,691–89,540)	82.38 (64.63–103.8)	−1.31 (−1.47 to −1.16)
Iraq	23,952 (19,209–30,206)	132.67 (106.29–168.23)	40,196 (32,071–51,440)	96.73 (76.94–123.53)	0.49 (−0.55 to 1.55)
Ireland	6,793 (5,125–8,959)	185.28 (139.8–245.14)	8,658 (6,249–11,977)	163.75 (116.8–226)	−0.46 (−0.65 to −0.26)
Israel	7,960 (6,141–10,313)	159.46 (123.09–206.69)	14,213 (10,665–18,957)	144.08 (108.31–193.14)	−0.4 (−0.75 to −0.04)
Italy	170,364 (125,230–228,938)	266.42 (197.84–359.88)	140,877 (100,249–196,323)	191.63 (138.62–263.18)	−1.22 (−1.28 to −1.15)
Jamaica	1,522 (1,171–2,048)	63.42 (48.44–85.22)	1,846 (1,422–2,446)	64.5 (49.86–85.55)	−0.12 (−0.27 to 0.02)
Japan	215,399 (165,973–279,877)	162.14 (125.59–209.56)	182,527 (135,985–241,264)	111.83 (84.81–145.17)	−1.32 (−1.47 to −1.17)
Jordan	2,766 (2,158–3,494)	73.79 (58.22–93.05)	7,441 (5,751–9,609)	59.23 (46.45–76.7)	−0.69 (−0.75 to −0.64)
Kazakhstan	22,611 (17,737–28,760)	134.58 (105.95–171.39)	22,403 (17,397–29,086)	117.74 (91.17–152.77)	−0.35 (−0.46 to −0.23)
Kenya	8,896 (7,064–11,488)	45.26 (36.14–57.34)	18,633 (14,776–23,724)	44.52 (35.71–56.86)	−0.13 (−0.33 to 0.06)
Kiribati	26 (21–33)	36.2 (28.72–46.01)	36 (29–46)	30.87 (24.61–38.96)	−0.8 (−1.14 to −0.45)
Kuwait	3,231 (2,033–5,319)	179.71 (113.24–297.73)	3,808 (2,915–5,036)	76.77 (58.56–100.91)	−1.47 (−2.17 to −0.76)
Kyrgyzstan	5,415 (4,308–6,882)	119.16 (95.11–152.02)	5,569 (4,390–7,084)	80.04 (63.59–101.35)	−1.49 (−1.6 to −1.38)
Lao People's Democratic Republic	3,026 (2,248–4,358)	73.82 (56.22–102.02)	3,510 (2,832–4,393)	49.14 (39.44–61.76)	−0.68 (−0.85 to −0.51)
Latvia	6,965 (5,345–9,051)	255.73 (195.95–331.32)	2,912 (2,220–3,846)	149.86 (114.65–196.12)	−2.26 (−2.46 to −2.06)
Lebanon	3,998 (2,814–5,984)	138.03 (97.49–206.18)	3,632 (2,850–4,709)	63.39 (49.72–81.96)	−1.88 (−2.43 to −1.32)
Lesotho	672 (536–836)	46.37 (37.51–57.28)	1,126 (896–1,414)	61.1 (48.6–76.43)	0.97 (0.78–1.17)
Liberia	5,008 (2,464–9,833)	184.52 (93.48–358.48)	1,815 (1,438–2,316)	38.03 (30.3–48.26)	−4.09 (−5.59 to −2.57)
Libya	3,638 (2,858–4,633)	88.68 (69.98–111.81)	6,594 (5,223–8,280)	100.61 (77.94–129.02)	1.9 (1.04–2.78)
Lithuania	8,618 (6,590–11,328)	228.23 (175.06–299.95)	4,639 (3,484–6,214)	155.54 (117.06–205.13)	−1.47 (−1.68 to −1.26)
Luxembourg	1,095 (844–1,444)	265.32 (204.14–346.18)	1,514 (1,088–2,060)	205.53 (149.99–280.23)	−0.87 (−0.91 to −0.82)
Madagascar	4,654 (3,702–5,900)	40.29 (32.47–51.05)	9,169 (7,296–11,571)	34.92 (28.09–44.06)	−0.51 (−0.57 to −0.45)
Malawi	3,674 (2,947–4,677)	39.2 (31.46–49.28)	6,420 (5,054–8,166)	37.29 (29.56–47.42)	−0.25 (−0.32 to −0.18)
Malaysia	8,920 (7,120–11,256)	54.9 (43.98–69.05)	17,616 (13,976–22,357)	54.78 (43.49–69.8)	−0.07 (−0.1 to −0.04)
Maldives	119 (93–154)	60.05 (46.72–77.74)	299 (230–399)	56.63 (43.22–75.42)	−0.18 (−0.72 to 0.38)
Mali	4,736 (3,743–6,123)	56.32 (44.73–71.67)	11,399 (9,039–14,384)	51.42 (40.77–64.55)	−0.72 (−1.66 to 0.24)
Malta	733 (546–986)	196.1 (145.67–264.4)	888 (640–1,225)	177.94 (128.21–243.44)	−0.21 (−0.38 to −0.04)
Marshall Islands	20 (16–25)	51.2 (40.73–64.42)	27 (21–35)	50.37 (39.51–64.08)	−0.07 (−0.12 to −0.02)
Mauritania	953 (771–1,169)	50.82 (41.12–62.36)	1,672 (1,329–2,104)	42.88 (34.27–53.99)	−0.57 (−0.62 to −0.52)
Mauritius	515 (410–652)	46.1 (36.83–58.15)	617 (484–807)	45.83 (36.01–59.75)	0.25 (0.14–0.36)
Mexico	122,602 (92,791–162,541)	146.34 (111.08–192.26)	122,771 (93,807–161,076)	94.38 (71.98–123.8)	−0.46 (−0.84 to −0.07)
Micronesia (Federated States of)	49 (39–61)	53.19 (42.53–66.52)	55 (44–71)	55.9 (44.13–71.49)	0.13 (−0.33 to 0.58)
Monaco	53 (39–71)	157.87 (117.9–209.95)	66 (48–92)	153.05 (111.77–207.97)	−0.04 (−0.15 to 0.07)
Mongolia	2,375 (1,863–2,993)	109.12 (86.06–138.65)	4,244 (3,307–5,480)	125.16 (97.4–160.78)	0.56 (0.46–0.66)
Montenegro	1,202 (914–1,593)	189.86 (144.45–251.88)	1,005 (761–1,326)	160.28 (121.25–213.89)	−0.54 (−0.61 to −0.47)
Morocco	24,480 (19,567–30,890)	97.18 (78.03–123.09)	30,552 (24,135–38,977)	81.9 (64.48–104.43)	−0.65 (−0.73 to −0.57)
Mozambique	8,091 (5,635–12,304)	59.79 (43.24–86.61)	13,153 (10,484–16,638)	47.47 (38.31–58.88)	−0.11 (−0.44 to 0.23)
Myanmar	29,802 (23,983–36,959)	75.08 (61–93.48)	49,369 (37,079–64,558)	87.49 (65.97–114.05)	0.03 (−1.05 to 1.11)
Namibia	628 (500–787)	48.42 (39.08–60.27)	1,151 (922–1,447)	48.76 (39.06–61.16)	−0.17 (−0.35 to 0.02)
Nauru	6 (4–7)	59.22 (47.26–74.44)	7 (5–8)	64.89 (51.01–82.7)	0.2 (0.11–0.28)
Nepal	13,152 (9,803–17,427)	73.8 (55.46–99.1)	24,513 (17,641–33,809)	80.54 (58.27–110.61)	0.14 (−0.54 to 0.82)
Netherlands	24,764 (18,576–32,885)	153.41 (115.47–202.38)	39,899 (27,868–55,959)	163.13 (117.21–227.25)	0.48 (0.06–0.9)
New Zealand	10,620 (8,186–13,593)	302.94 (234.39–390.41)	13,995 (10,466–18,177)	252.3 (192.95–328.15)	−0.52 (−0.64 to −0.41)
Nicaragua	3,205 (2,469–4,166)	78.72 (61.93–100.52)	4,442 (3,422–5,732)	69.28 (53.64–89.42)	−0.45 (−0.84 to −0.05)
Niger	3,765 (3,035–4,689)	49.33 (40.05–61.4)	11,338 (8,944–14,394)	49.66 (39.2–62.07)	0.15 (0.01–0.29)
Nigeria	38,001 (29,804–48,941)	44.88 (35.78–57.18)	87,752 (69,228–112,060)	42.8 (33.98–54.77)	−0.14 (−0.26 to −0.02)
Niue	1 (1–1)	50.27 (39.53–64.24)	1 (1–1)	49.42 (38.2–64.71)	−0.22 (−0.72 to 0.28)
North Macedonia	3,092 (2,346–4,083)	153.78 (116.93–202.72)	3,059 (2,299–4,124)	140.91 (105.08–187.53)	−0.34 (−0.48 to −0.2)
Northern Mariana Islands	33 (26–42)	74.95 (58.96–95.78)	34 (27–45)	71.52 (55.49–93.87)	−0.2 (−0.3 to −0.1)
Norway	11,492 (8,553–15,499)	227.93 (171.29–305.95)	12,722 (8,968–17,949)	177.08 (127.49–243.44)	−0.82 (−0.94 to −0.69)
Oman	2,652 (2,046–3,355)	144.05 (112.07–182.29)	4,912 (3,857–6,260)	108.56 (85.25–138.6)	−1.06 (−1.21 to −0.92)
Pakistan	44,784 (35,134–57,148)	43.28 (34.31–54.94)	90,062 (71,906–113,583)	40.17 (32.1–50.78)	0.07 (−0.45 to 0.6)
Palau	12 (10–16)	84 (65.26–109.51)	17 (13–23)	92.38 (69.93–122.27)	0.31 (0.28–0.34)
Palestine	2,232 (1,584–3,238)	103.81 (74.86–146.79)	4,084 (3,133–5,297)	78.15 (60.16–101.52)	−0.91 (−2.5 to 0.71)
Panama	2,037 (1,582–2,628)	83.99 (65.98–107.23)	3,057 (2,360–3,925)	71.22 (54.94–91.58)	−0.62 (−0.67 to −0.58)
Papua New Guinea	1,937 (1,517–2,469)	53.23 (41.51–68.82)	5,945 (4,605–7,739)	64.48 (49.5–84.64)	0.11 (−0.49 to 0.71)
Paraguay	3,736 (2,830–4,961)	92.27 (70.13–120.96)	6,477 (5,037–8,389)	88.82 (69.22–115.34)	−0.15 (−0.23 to −0.07)
Peru	20,533 (15,463–27,904)	90.74 (69.53–120.11)	25,163 (19,774–32,348)	68.34 (53.68–87.77)	−0.56 (−0.78 to −0.34)
Philippines	44,917 (34,859–56,940)	72.63 (56.89–90.77)	54,770 (43,002–70,393)	49.27 (38.63–63.66)	−0.76 (−1.06 to −0.46)
Poland	79,230 (61,002–105,746)	204.06 (157.79–270.28)	66,322 (50,119–89,330)	159.02 (120.2–212.46)	−0.89 (−0.98 to −0.79)
Portugal	24,116 (18,980–30,588)	231.1 (181.36–293.71)	17,492 (12,875–23,577)	135.2 (102.12–177.33)	−2.01 (−2.1 to −1.91)
Puerto Rico	2,737 (2,168–3,453)	75.5 (59.86–95.42)	3,005 (2,286–3,979)	82.66 (64.67–106.74)	0.39 (0.2–0.58)
Qatar	572 (454–725)	115.75 (91.43–146.33)	3,453 (2,641–4,571)	101.82 (78.88–133.52)	−0.32 (−0.44 to −0.2)
Republic of Korea	122,439 (96,375–152,269)	274.37 (217.04–341.47)	114,116 (84,224–153,481)	178.85 (135.48–238.19)	−1.67 (−1.77 to −1.57)
Republic of Moldova	8,171 (6,494–10,364)	182.37 (145.13–230.78)	4,195 (3,277–5,492)	114.46 (89.73–148.36)	−1.76 (−1.85 to −1.66)
Romania	52,178 (39,753–69,222)	223.93 (171.14–296.4)	31,720 (23,788–42,580)	167.09 (125.05–223.47)	−1.1 (−1.17 to −1.04)
Russian Federation	321,727 (251,711–417,516)	211.06 (164.06–273.43)	248,916 (193,326–327,880)	168.07 (130.22–220.92)	−1.06 (−1.41 to −0.7)
Rwanda	6,980 (4,419–11,611)	92.61 (61.7–148.85)	4,694 (3,736–5,890)	39.51 (31.52–49.74)	−3.86 (−5.77 to −1.92)
Saint Kitts and Nevis	29 (23–39)	72.52 (56.65–94.65)	45 (34–60)	76.37 (57.77–101.95)	0.25 (0.11–0.39)
Saint Lucia	81 (64–106)	60.17 (47.58–77.38)	112 (87–147)	62.46 (48.43–81.07)	0.08 (−0.05 to 0.21)
Saint Vincent and the Grenadines	66 (52–85)	60.49 (47.72–77.65)	79 (62–103)	67.74 (53.13–88.63)	0.27 (0.12–0.43)
Samoa	87 (68–114)	54.64 (43.54–69.52)	99 (77–127)	50.43 (39.46–65.17)	0.1 (−0.76 to 0.96)
San Marino	43 (32–56)	169.89 (128.26–222.04)	62 (46–86)	161.4 (119.14–219.06)	−0.09 (−0.18 to 0)
Sao Tome and Principe	61 (48–79)	51.01 (40.27–65.97)	131 (100–171)	66.64 (50.85–86.59)	0.63 (0.56–0.71)
Saudi Arabia	26,521 (20,724–33,856)	180.73 (142.07–232.31)	91,161 (69,166–120,850)	211.6 (162.11–281.06)	0.68 (0.6–0.77)
Senegal	2,967 (2,377–3,776)	41 (33.04–51.34)	5,571 (4,374–7,094)	39.63 (31.45–50.48)	−0.22 (−0.36 to −0.08)
Serbia	15,469 (11,891–20,370)	162.1 (124.43–211.84)	12,889 (9,617–17,451)	140.26 (104.53–188.34)	−0.79 (−1.23 to −0.35)
Seychelles	41 (32–52)	57.06 (45.05–73.01)	55 (43–71)	50.48 (39.89–65)	−0.45 (−0.58 to −0.32)
Sierra Leone	1,714 (1,376–2,143)	41.3 (33.26–51.85)	3,195 (2,545–4,064)	39.69 (31.76–50.2)	−2.85 (−4.14 to −1.55)
Singapore	4,836 (3,774–6,193)	150.44 (117.65–190.78)	7,137 (5,335–9,423)	123.62 (93.61–164.59)	−0.78 (−0.92 to −0.64)
Slovakia	12,660 (9,691–16,815)	233.98 (179.39–311.13)	11,325 (8,540–15,156)	192.79 (143.53–259.63)	−0.6 (−0.64 to −0.56)
Slovenia	5,874 (4,495–7,816)	282.83 (216.41–377.42)	5,322 (3,997–7,294)	213.65 (159.02–288.71)	−0.51 (−0.77 to −0.26)
Solomon Islands	196 (153–251)	72.42 (56.59–93.47)	498 (378–658)	85.03 (64.28–113.69)	0.58 (0.44–0.71)
Somalia	7,127 (4,416–12,398)	87.94 (58.52–144.36)	11,880 (9,094–16,390)	58.37 (45.79–75.94)	0.2 (−0.74 to 1.14)
South Africa	29,672 (23,121–38,079)	80.95 (63.25–104.56)	31,928 (25,173–40,255)	54.12 (42.73–67.79)	−1.5 (−1.62 to −1.37)
South Sudan	2,326 (1,861–2,947)	42 (33.64–53.25)	4,750 (3,635–6,340)	50.33 (39.52–64.72)	0.72 (−0.48 to 1.93)
Spain	77,619 (61,063–97,987)	191.71 (151.59–243.18)	81,320 (58,847–110,345)	158.12 (114.89–215.16)	−0.7 (−0.81 to −0.58)
Sri Lanka	28,300 (17,395–46,926)	155.33 (98.28–252.74)	17,949 (13,663–23,727)	77.08 (59.01–101.98)	−1.93 (−3 to −0.85)
Sudan	23,523 (16,862–33,683)	113.63 (83.49–159.34)	29,997 (23,661–38,013)	70.13 (55.83–87.72)	−1.17 (−1.58 to −0.76)
Suriname	215 (170–269)	54.95 (43.78–68.84)	341 (270–432)	58.68 (46.53–74.27)	0.27 (0.19–0.35)
Sweden	19,940 (14,626–26,659)	198.62 (147.23–266)	22,334 (15,772–31,242)	160.25 (115.29–218.92)	−0.66 (−0.8 to −0.53)
Switzerland	24,916 (18,073–34,029)	315.48 (232.88–429.03)	25,335 (17,771–35,370)	219.89 (157.7–306.38)	−1.4 (−1.5 to −1.29)
Syrian Arab Republic	8,567 (6,660–10,968)	66.69 (51.93–84.94)	11,444 (8,546–15,486)	80.72 (61.05–108.28)	5.24 (3.34–7.19)
Taiwan (Province of China)	16,550 (12,764–21,155)	81.23 (62.66–103.95)	12,532 (9,539–16,693)	43.42 (33.66–56.82)	−2.54 (−2.81 to −2.28)
Tajikistan	5,531 (4,328–7,056)	100.64 (79.65–128.12)	7,540 (5,899–9,703)	72.61 (57.07–93.07)	−2.63 (−3.58 to −1.67)
Thailand	45,265 (36,503–55,894)	78.32 (63.65–97.06)	50,722 (39,757–65,701)	70.18 (54.86–89.82)	−0.58 (−0.74 to −0.41)
Timor-Leste	955 (560–1,647)	115.32 (69.86–198.13)	639 (506–808)	48.42 (38.38–61.49)	−4.61 (−6.07 to −3.12)
Togo	1,508 (1,212–1,884)	45.23 (36.29–56.56)	3,258 (2,586–4,072)	43.87 (35.18–55.07)	−0.2 (−0.37 to −0.03)
Tokelau	1 (1–1)	45 (35.5–57.26)	1 (0–1)	44.67 (34.62–58.8)	−0.06 (−0.11 to 0)
Tonga	38 (30–49)	42.23 (32.93–53.93)	36 (28–47)	35.75 (28.03–46.65)	−0.43 (−0.71 to −0.15)
Trinidad and Tobago	773 (620–978)	64.19 (51.62–81.04)	871 (699–1,101)	64.23 (51.34–81.94)	0.43 (0.25–0.61)
Tunisia	7,569 (5,962–9,705)	90.31 (71.14–116.7)	9,599 (7,557–12,331)	80.17 (62.78–102.72)	−0.37 (−0.44 to −0.31)
Turkey	41,806 (33,227–53,421)	71.89 (57.33–91.75)	56,230 (43,612–74,021)	66.62 (51.65–88.09)	−0.3 (−0.48 to −0.12)
Turkmenistan	3,586 (2,811–4,582)	90.76 (72.05–115.82)	3,916 (3,022–5,162)	74.2 (57.29–97.87)	−0.82 (−1.04 to −0.61)
Tuvalu	5 (4–6)	51.89 (41.34–64.94)	6 (5–8)	52.34 (41.24–66.54)	−0.38 (−0.55 to −0.21)
Uganda	7,750 (6,126–9,986)	46.28 (37.16–59.22)	15,605 (12,341–19,773)	40.69 (32.6–51.08)	−0.57 (−0.89 to −0.25)
Ukraine	112,931 (85,975–151,872)	214.66 (162.57–286.98)	76,365 (59,412–100,835)	176.98 (137.93–233.49)	−1.06 (−1.25 to −0.87)
United Arab Emirates	2,185 (1,718–2,772)	112.86 (89.14–142.77)	12,022 (9,332–15,431)	105.48 (82.25–135.27)	−0.2 (−0.23 to −0.17)
United Kingdom	101,309 (75,597–134,798)	164.64 (123.03–217.72)	120,354 (85,907–168,140)	147.09 (106.32–201.4)	−0.32 (−0.46 to −0.17)
United Republic of Tanzania	10,500 (8,349–13,348)	42.45 (33.96–53.7)	21,482 (16,760–27,595)	40.4 (31.84–51)	−0.18 (−0.21 to −0.14)
United States of America	479,158 (372,812–600,534)	178.52 (138.33–225.23)	679,567 (509,038–895,942)	160.16 (123.69–208.11)	−0.42 (−0.55 to −0.3)
United States Virgin Islands	70 (56–90)	67.63 (53.87–85.59)	61 (48–78)	63.81 (50.28–82.72)	−0.42 (−0.61 to −0.24)
Uruguay	5,600 (4,304–7,359)	176.07 (135.63–232.5)	5,950 (4,533–7,665)	162.38 (124.04–208.84)	−0.36 (−0.41 to −0.32)
Uzbekistan	21,057 (16,637–26,893)	96.88 (76.91–123.65)	28,113 (22,181–36,068)	81.18 (63.98–104.28)	−0.68 (−0.81 to −0.55)
Vanuatu	61 (48–76)	44.51 (35.36–55.76)	130 (103–164)	44.66 (35.35–56.67)	−0.13 (−0.48 to 0.22)
Venezuela (Bolivarian Republic of)	20,720 (16,032–27,004)	107.17 (83.69–136.74)	24,161 (18,824–31,112)	93.1 (72.4–120.05)	−0.65 (−1.05 to −0.24)
Viet Nam	39,578 (31,375–50,208)	64.91 (51.9–82.55)	75,020 (58,490–97,253)	75.33 (58.33–97.96)	0.6 (0.5–0.7)
Yemen	11,111 (8,716–13,927)	85.49 (67.89–107.66)	52,655 (33,338–90,183)	148.31 (97.5–244.33)	2.18 (1.44–2.92)
Zambia	3,203 (2,537–4,031)	43.56 (35.19–54.52)	7,320 (5,741–9,333)	44.67 (35.58–57.07)	0.07 (−0.01 to 0.14)
Zimbabwe	4,163 (3,324–5,183)	46.43 (37.71–57.07)	6,030 (4,857–7,462)	43.21 (35.01–53.57)	−0.31 (−0.43 to −0.2)

**Table 2:** The number of prevalence cases and the age-standardized prevalence rate attributable to fracture of vertebral column in 1990 and 2021, and its trends from 1990 to 2021 globally.

	Number of prevalence cases (95% UI) in 1990	The age-standardized prevalence rate/100,000 (95% UI) in 1990	Number of prevalence cases (95% UI) in 2021	The age-standardized prevalence rate/100,000 (95% UI) in 2021	EAPC (95% CI)
Global	3,400,460 (2,958,279–3,925,843)	81.55 (71.55–93.04)	5,371,438 (4,703,837–6,196,132)	65.19 (56.89–75.28)	−0.79 (−0.83 to −0.76)
Sex					
Female	1,564,020 (1,355,790–1,813,812)	72.6 (62.91–83.13)	2,677,008 (2,296,111–3,095,114)	59.57 (51.06–69.42)	−0.7 (−0.73 to −0.66)
Male	1,836,440 (1,589,976–2,128,888)	87.77 (77.26–99.64)	2,694,430 (2,337,400–3,085,527)	69.58 (61.29–79.73)	−0.83 (−0.87 to −0.79)
Age					
<5 years	88,350 (59,082–121,413)	14.25 (9.53–19.58)	53,349 (35,326–73,935)	8.11 (5.37–11.23)	−1.92 (−2.09 to −1.75)
5–9 years	93,748 (60,559–133,455)	16.07 (10.38–22.87)	74,895 (47,361–108,290)	10.9 (6.89–15.76)	−1.16 (−1.25 to −1.06)
10–14 years	101,403 (67,414–145,695)	18.93 (12.58–27.2)	90,631 (59,761–132,296)	13.6 (8.96–19.85)	−1.14 (−1.21 to −1.07)
15–19 years	150,016 (103,278–208,229)	28.88 (19.88–40.09)	127,020 (85,784–179,065)	20.36 (13.75–28.7)	−1.13 (−1.23 to −1.04)
20–24 years	179,297 (131,103–240,415)	36.44 (26.64–48.86)	153,223 (109,285–209,521)	25.66 (18.3–35.09)	−1.25 (−1.32 to −1.18)
25–29 years	182,049 (138,833–235,316)	41.13 (31.37–53.16)	167,418 (125,058–220,474)	28.46 (21.26–37.47)	−1.28 (−1.33 to −1.22)
30–34 years	182,678 (146,073–232,671)	47.4 (37.9–60.37)	197,229 (153,402–257,216)	32.63 (25.38–42.55)	−1.36 (−1.4 to −1.31)
35–39 years	189,343 (154,002–238,083)	53.75 (43.72–67.59)	215,469 (170,586–275,855)	38.42 (30.41–49.18)	−1.32 (−1.41 to −1.23)
40–44 years	185,250 (158,856–216,104)	64.66 (55.45–75.43)	224,335 (188,847–268,386)	44.84 (37.75–53.65)	−1.35 (−1.43 to −1.26)
45–49 years	175,622 (153,782–204,823)	75.64 (66.23–88.21)	255,086 (219,123–301,976)	53.87 (46.28–63.77)	−1.26 (−1.34 to −1.17)
50–54 years	198,815 (176,104–229,704)	93.53 (82.84–108.06)	304,241 (264,449–359,847)	68.38 (59.44–80.88)	−1.11 (−1.19 to −1.04)
55–59 years	216,405 (194,142–244,402)	116.85 (104.83–131.97)	360,662 (317,688–415,973)	91.14 (80.28–105.12)	−0.86 (−0.91 to −0.8)
60–64 years	241,938 (213,706–270,839)	150.64 (133.06–168.63)	397,848 (346,578–456,368)	124.31 (108.29–142.59)	−0.68 (−0.73 to −0.63)
65–69 years	250,142 (219,924–282,859)	202.36 (177.92–228.83)	456,735 (397,026–526,310)	165.58 (143.93–190.8)	−0.58 (−0.61 to −0.55)
70–74 years	225,737 (195,768–261,276)	266.64 (231.24–308.61)	492,187 (418,953–584,709)	239.11 (203.53–284.06)	−0.5 (−0.56 to −0.44)
75–79 years	257,169 (218,310–304,233)	417.78 (354.65–494.24)	469,524 (394,536–561,916)	356.01 (299.15–426.07)	−0.54 (−0.62 to −0.46)
80–84 years	234,183 (185,854–292,511)	661.98 (525.37–826.86)	501,405 (396,937–631,104)	572.49 (453.21–720.58)	−0.53 (−0.58 to −0.48)
85–89 years	157,043 (127,195–200,332)	1039.26 (841.74–1325.73)	431,696 (347,577–548,748)	944.18 (760.2–1200.19)	−0.34 (−0.43 to −0.24)
90–94 years	68,584 (55,958–86,049)	1600.49 (1305.85–2008.06)	272,163 (219,887–344,061)	1521.36 (1229.15–1923.27)	−0.12 (−0.22 to −0.02)
95 + years	22,686 (18,519–28,935)	2228.27 (1818.97–2842.12)	126,320 (101,813–159,376)	2317.67 (1868.01–2924.16)	0.04 (−0.08–0.17)
SDI region					
High-middle SDI	819,302 (710,171–945,556)	83.07 (72.53–95.12)	1,086,362 (953,352–1,244,962)	64.26 (55.8–74.54)	−1.01 (−1.09 to −0.92)
High SDI	1,602,083 (1,418,256–1,794,032)	156.1 (137.89–174.99)	2,469,882 (2,144,431–2,817,433)	131.65 (114.84–149.24)	−0.61 (−0.63 to −0.59)
Low-middle SDI	316,183 (253,324–389,439)	38.55 (32.22–45.39)	567,486 (471,693–669,476)	36.65 (31.13–42.92)	−0.26 (−0.32 to −0.19)
Low SDI	121,291 (90,884–166,601)	33.91 (27.73–43.43)	246,714 (190,571–325,486)	33.49 (27.67–41.42)	−0.04 (−0.15–0.06)
Middle SDI	538,131 (438,957–652,717)	39.7 (33.66–46.41)	996,724 (846,944–1,163,380)	39.57 (33.59–46.45)	−0.05 (−0.14–0.04)
GBD region					
Advanced Health System	2,190,144 (1,933,975–2,454,689)	145.83 (128.22–165.17)	3,082,311 (2,684,185–3,499,676)	122.84 (106.99–140.14)	−0.65 (−0.7 to −0.6)
Africa	147,968 (113,972–194,626)	31.1 (25.56–38.03)	254,007 (203,935–314,130)	26.6 (22.39–31.69)	−0.52 (−0.62 to −0.42)
African Region	110,069 (83,901–147,308)	29.05 (23.97–35.9)	190,207 (150,796–238,534)	24.7 (20.64–29.67)	−0.59 (−0.72 to −0.45)
America	742,794 (653,443–845,340)	115.49 (102.19–130.14)	1,303,854 (1,138,984–1,470,467)	102.35 (89.05–116.25)	−0.35 (−0.39 to −0.32)
Andean Latin America	11,189 (8,632–14,621)	35.24 (28.42–43.9)	20,589 (16,961–25,108)	32.29 (26.87–39.16)	−0.21 (−0.26 to −0.15)
Asia	1,197,126 (1,012,746–1,412,641)	51.01 (44.34–58.68)	2,248,522 (1,933,550–2,609,314)	47.5 (40.9–55.3)	−0.35 (−0.4 to −0.3)
Australasia	43,067 (38,208–49,016)	196.89 (174.25–224.91)	86,326 (74,702–98,843)	182.32 (158.11–208.92)	−0.1 (−0.17 to −0.02)
Basic Health System	767,969 (633,100–915,692)	41.19 (35.21–47.78)	1,380,919 (1,183,891–1,600,623)	40.61 (34.62–47.32)	−0.08 (−0.17–0.01)
Caribbean	10,229 (8,375–12,362)	34.05 (28.69–40.37)	21,949 (18,704–25,857)	42.64 (36.12–50.54)	0.81 (0.39–1.23)
Central Africa	10,056 (7,937–12,269)	22.5 (19.2–26.1)	29,125 (22,473–37,838)	26.41 (21.47–32.94)	−0.01 (−0.41–0.39)
Central Asia	27,995 (22,017–34,946)	45.45 (36.96–55.12)	34,439 (27,868–42,185)	37.62 (30.74–45.71)	−0.73 (−0.83 to −0.62)
Central Europe	123,290 (102,594–148,815)	93.37 (77.43–113.72)	116,959 (100,857–137,786)	71.09 (58.61–87.02)	−1.05 (−1.12 to −0.99)
Central Latin America	75,567 (59,109–94,294)	58.28 (48.51–69.95)	103,659 (87,349–124,125)	40.95 (34.49–49.06)	−0.77 (−0.94 to −0.61)
Central Sub-Saharan Africa	9,839 (7,674–12,606)	25.31 (21.06–30.39)	23,216 (18,095–29,626)	25.97 (21.33–32.14)	−0.34 (−0.7–0.02)
Commonwealth High Income	201,098 (177,701–225,003)	148.71 (131.13–167.98)	338,670 (292,332–390,336)	140.4 (122.12–160.88)	−0.09 (−0.18–0.01)
Commonwealth Low Income	30,567 (23,695–39,815)	19.51 (16.11–23.89)	58,875 (47,081–72,742)	19.32 (16.03–23.37)	−0.67 (−1.01 to −0.33)
Commonwealth Middle Income	323,626 (260,533–389,347)	41.39 (34.8–48.75)	672,842 (557,781–803,491)	41.85 (35.49–49.81)	−0.03 (−0.08–0.02)
East Asia	347,489 (287,374–411,101)	34.8 (29.75–40.39)	732,220 (632,696–852,696)	39.83 (33.85–46.73)	0.17 (−0.11–0.46)
East Asia & Pacific—WB	797,232 (690,674–915,528)	54.64 (48.02–62.2)	1,414,696 (1,243,086–1,628,908)	48.41 (42.06–56.13)	−0.57 (−0.66 to −0.48)
Eastern Africa	50,341 (34,202–77,711)	35.63 (27.21–49.16)	67,349 (51,048–91,919)	26.24 (21.3–33.71)	−0.76 (−0.97 to −0.54)
Eastern Europe	215,211 (180,063–258,946)	87.28 (71.85–106.35)	190,639 (162,961–224,778)	72.13 (59.51–87.22)	−0.95 (−1.26 to −0.65)
Eastern Mediterranean Region	122,727 (96,231–159,544)	41.65 (34.08–51.83)	257,920 (201,825–335,073)	40.7 (33.1–51.46)	0.16 (0.08–0.25)
Eastern Sub-Saharan Africa	49,895 (34,068–76,380)	33.02 (25.13–45.62)	69,295 (52,053–95,131)	25.57 (20.56–33.54)	−0.69 (−0.92 to −0.46)
Europe	1,307,800 (1,141,839–1,485,502)	141.01 (122.76–162.65)	1,558,605 (1,351,031–1,778,491)	113.74 (98.5–131.53)	−0.8 (−0.89 to −0.71)
Europe & Central Asia—WB	1,325,761 (1,156,261–1,507,751)	137.65 (119.8–158.77)	1,581,896 (1,370,681–1,804,932)	110.28 (95.44–127.44)	−0.82 (−0.91 to −0.73)
European Region	1,333,300 (1,162,878–1,516,593)	137.45 (119.63–158.54)	1,596,813 (1,384,230–1,821,679)	110.25 (95.42–127.39)	−0.82 (−0.91 to −0.73)
High-income Asia Pacific	293,045 (262,800–328,979)	153.14 (136.73–172.58)	402,262 (355,326–451,412)	107.14 (95.11–121.49)	−1.36 (−1.47 to −1.25)
High-income North America	518,876 (460,240–580,940)	157.26 (139.37–176.33)	948,836 (822,350–1,080,753)	157.6 (138.08–178.17)	−0.01 (−0.08–0.07)
Latin America & Caribbean—WB	225,256 (185,238–272,064)	64.34 (55.16–75.01)	357,235 (309,606–413,746)	51.87 (44.86–60.33)	−0.57 (−0.67 to −0.48)
Limited Health System	406,219 (322,003–504,318)	38.55 (32.25–45.71)	825,586 (682,604–987,097)	38.74 (32.8–45.88)	−0.06 (−0.12–0)
Middle East & North Africa—WB	105,693 (83,857–132,402)	53.59 (44.84–64.22)	208,500 (168,426–260,415)	49.37 (40.64–60.15)	−0.03 (−0.1–0.05)
Minimal Health System	32,658 (22,430–52,189)	31.88 (23.7–48.3)	78,351 (55,610–116,572)	33.84 (25.53–48.36)	−0.06 (−0.26–0.14)
North Africa and Middle East	127,975 (100,454–164,952)	46.75 (38.24–57.96)	258,874 (204,786–333,164)	46.08 (37.31–57.72)	0.17 (0.08–0.25)
North America	518,795 (460,168–580,844)	157.23 (139.34–176.3)	948,729 (822,257–1,080,633)	157.57 (138.05–178.13)	−0.01 (−0.08–0.07)
Northern Africa	37,897 (29,970–46,579)	39.68 (33.02–46.78)	62,632 (51,187–74,497)	34.59 (29.2–40.58)	−0.43 (−0.47 to −0.4)
Oceania	1,099 (879–1,332)	25.82 (22.07–30.06)	3,015 (2,451–3,632)	31.39 (26.72–36.65)	0.4 (0.16–0.65)
Region of the Americas	742,794 (653,443–845,340)	115.49 (102.19–130.14)	1,303,854 (1,138,984–1,470,467)	102.35 (89.05–116.25)	−0.35 (−0.39 to −0.32)
South-East Asia Region	366,444 (296,005–444,091)	41.46 (34.73–48.74)	720,550 (599,500–860,759)	40.29 (34.14–48.03)	−0.23 (−0.29 to −0.16)
South Asia	296,596 (238,796–359,584)	41.55 (34.95–49.08)	625,367 (518,858–748,472)	42.04 (35.53–50.11)	−0.08 (−0.15 to −0.02)
South Asia—WB	312,784 (250,705–387,367)	42.06 (35.2–49.99)	662,858 (548,708–789,921)	43.04 (36.2–51.25)	−0.05 (−0.11–0.01)
Southeast Asia	120,892 (96,360–151,618)	34.11 (28.72–40.69)	202,255 (170,360–239,427)	31.12 (26.53–36.58)	−0.32 (−0.44 to −0.21)
Southern Africa	23,438 (18,634–29,877)	33.15 (27.7–39.97)	33,633 (27,531–41,167)	25.09 (21.37–29.8)	−1.07 (−1.2 to −0.94)
Southern Latin America	53,876 (47,984–60,573)	115.51 (103.12–129.32)	91,462 (82,273–102,207)	114.53 (101.87–129.44)	−0.02 (−0.1–0.06)
Southern Sub-Saharan Africa	14,225 (11,574–17,083)	35.73 (30.27–41.44)	18,239 (15,229–21,548)	25.56 (21.8–29.61)	−1.27 (−1.4 to −1.13)
Sub-Saharan Africa—WB	110,346 (83,334–150,689)	28.83 (23.65–36.19)	191,819 (151,078–242,624)	24.7 (20.54–29.97)	−0.52 (−0.65 to −0.4)
Tropical Latin America	74,760 (59,826–92,363)	59.75 (49.65–71.5)	120,349 (101,926–142,082)	49.15 (41.4–58.44)	−0.62 (−0.71 to −0.53)
Western Africa	26,236 (20,524–32,496)	21.95 (18.48–25.78)	61,268 (49,007–75,734)	22.43 (19.07–26.13)	0.01 (−0.06–0.09)
Western Europe	956,120 (836,666–1,080,019)	188.54 (164.92–212.98)	1,232,001 (1,066,030–1,413,658)	151.43 (131.99–172.21)	−0.75 (−0.8 to −0.71)
Western Pacific Region	713,014 (620,742–816,430)	58.33 (51.42–66.22)	1,287,115 (1,127,353–1,475,386)	51.82 (45.03–59.87)	−0.57 (−0.68 to −0.47)
Western Sub-Saharan Africa	29,225 (22,896–36,309)	22.07 (18.6–26.13)	69,487 (55,451–85,899)	22.6 (19.2–26.36)	0.02 (−0.04–0.09)
World Bank High Income	1,959,642 (1,730,708–2,197,375)	161.83 (142.46–181.68)	2,862,545 (2,485,588–3,261,499)	134.56 (117.53–152.76)	−0.67 (−0.69 to −0.64)
World Bank Low Income	83,972 (59,710–124,806)	34.29 (26.25–47.97)	159,567 (116,184–228,318)	31.84 (24.59–43.69)	−0.1 (−0.27–0.08)
World Bank Lower Middle Income	570,067 (460,171–696,013)	40.24 (33.84–47.14)	1,037,229 (863,325–1,225,307)	37.89 (32.19–44.45)	−0.31 (−0.36 to −0.25)
World Bank Upper Middle Income	783,304 (648,665–930,709)	45.8 (39.07–53.3)	1,307,820 (1,123,186–1,516,296)	44.14 (37.47–51.73)	−0.25 (−0.33 to −0.17)
Country					
Afghanistan	7,517 (3,507–18,404)	94.11 (42.44–240.74)	25,317 (14,068–46,296)	106.68 (58.96–200.66)	−0.01 (−0.3–0.29)
Albania	1,973 (1,455–2,692)	66.49 (51.13–87.46)	1,868 (1,541–2,294)	59.86 (47.25–75.27)	−0.64 (−0.87 to −0.41)
Algeria	8,798 (6,829–10,904)	44.96 (37.5–53.45)	14,109 (11,597–17,016)	34.82 (29.12–41.33)	−1.06 (−1.16 to −0.96)
American Samoa	9 (7–11)	25.61 (21.53–30.26)	12 (10–14)	26.8 (22.75–31.24)	0.37 (−0.05–0.8)
Andorra	124 (108–141)	225.17 (195.09–256.63)	348 (301–399)	240.56 (207.28–276.91)	0.21 (0.16–0.25)
Angola	3,203 (2,120–4,934)	38.54 (28.1–55.1)	5,563 (4,177–7,525)	29.01 (22.58–39.12)	−1.29 (−1.73 to −0.85)
Antigua and Barbuda	16 (12–21)	27.28 (21.58–35.15)	28 (22–35)	29.22 (23.15–37.73)	0.08 (−0.17–0.32)
Argentina	34,875 (31,058–39,433)	109.7 (98.18–123.58)	53,827 (48,301–59,922)	103.85 (92.59–116.68)	−0.21 (−0.31 to −0.1)
Armenia	2,079 (1,596–2,725)	67.54 (52–90.37)	1,245 (1,022–1,516)	36.27 (29.24–44.47)	−1.95 (−2.57 to −1.33)
Australia	34,229 (30,339–38,980)	188.01 (166.4–214.48)	72,578 (62,633–83,223)	180.54 (156.14–206.91)	0.03 (−0.06–0.11)
Austria	23,596 (20,691–26,540)	228.05 (200.1–257.43)	26,896 (23,300–30,723)	168.66 (147.17–191.72)	−0.85 (−0.92 to −0.78)
Azerbaijan	2,394 (1,874–2,978)	35.61 (28.68–43.33)	3,230 (2,623–3,996)	30.32 (24.49–37.56)	−0.79 (−1.18 to −0.41)
Bahamas	57 (46–69)	26.74 (22.45–31.46)	114 (97–135)	28.83 (24.47–34.22)	0.38 (0.14–0.62)
Bahrain	120 (93–149)	30.59 (25.42–36.15)	394 (313–483)	27.87 (22.88–33.47)	−0.62 (−0.77 to −0.47)
Bangladesh	12,968 (10,112–16,439)	15.32 (12.65–18.3)	25,355 (20,429–30,716)	16.41 (13.44–19.64)	−0.57 (−1.18–0.04)
Barbados	59 (49–71)	21.8 (17.97–26.45)	91 (78–107)	23.46 (19.47–28.63)	0.09 (−0.04–0.23)
Belarus	8,383 (6,952–10,098)	73.74 (60.34–90.24)	9,050 (7,702–10,696)	74.48 (60.66–91.18)	−0.09 (−0.43–0.25)
Belgium	28,162 (24,768–31,803)	211.27 (185.86–239.72)	44,749 (38,396–51,244)	210.26 (183.37–241.23)	0.1 (−0.11–0.31)
Belize	42 (31–55)	26.99 (21.81–33.63)	125 (99–156)	32.35 (26.68–39.47)	0.35 (0.19–0.52)
Benin	684 (541–844)	21.24 (18–24.71)	1,800 (1,420–2,221)	21.43 (18.22–25.07)	−0.05 (−0.1–0)
Bermuda	16 (13–19)	25.89 (21.52–31.33)	25 (22–29)	25.62 (21.1–31.34)	−0.1 (−0.19–0)
Bhutan	95 (73–121)	23.57 (19.45–28.28)	227 (188–273)	35.36 (29.54–42.31)	1.22 (0.98–1.46)
Bolivia (Plurinational State of)	1,617 (1,261–2,000)	33.44 (27.52–39.63)	3,211 (2,604–3,956)	31.18 (25.99–37.36)	−0.27 (−0.3 to −0.25)
Bosnia and Herzegovina	3,130 (2,400–4,045)	71.26 (55.53–91.39)	2,704 (2,244–3,344)	62.79 (49.53–80.45)	−1.21 (−1.67 to −0.75)
Botswana	199 (154–246)	22.1 (18.45–26.06)	497 (404–589)	24.9 (21.06–28.85)	0.24 (0.04–0.44)
Brazil	73,513 (58,845–90,835)	60.33 (50.14–72.19)	117,791 (99,821–138,971)	49.44 (41.65–58.8)	−0.63 (−0.72 to −0.54)
Brunei Darussalam	293 (253–334)	175.88 (158.12–194.39)	530 (475–599)	135.5 (121.61–152.14)	−0.87 (−0.92 to −0.81)
Bulgaria	7,975 (6,663–9,612)	82.39 (66.88–101.87)	6,040 (5,126–7,206)	67.45 (53.58–84.64)	−0.72 (−0.75 to −0.68)
Burkina Faso	1,418 (1,132–1,718)	23.18 (19.84–26.74)	3,751 (2,899–4,937)	25.16 (21.18–30.33)	0.2 (0.1–0.31)
Burundi	775 (609–962)	21.43 (18.17–25.17)	3,885 (2,276–6,818)	44.48 (27.09–76.75)	0.41 (−1.1–1.94)
Cabo Verde	61 (50–74)	21.58 (18.04–25.14)	120 (101–143)	24.02 (20.31–28.25)	0.41 (0.38–0.44)
Cambodia	3,654 (2,238–7,034)	46.41 (31.97–83.38)	5,991 (4,495–9,012)	44.19 (34.53–64.79)	−0.21 (−0.3 to −0.11)
Cameroon	1,403 (1,124–1,718)	21.52 (18.48–24.99)	4,735 (3,711–5,890)	23.5 (20.02–27.57)	0.26 (0.21–0.32)
Canada	47,265 (41,976–52,854)	152.25 (135.19–170.33)	96,780 (82,403–111,900)	141.91 (121.85–162.42)	−0.07 (−0.14–0)
Central African Republic	424 (335–515)	23.48 (19.95–27.05)	1,417 (1,025–2,022)	32.49 (25.59–42.38)	1.15 (0.77–1.53)
Chad	1,220 (888–1,720)	26.19 (20.45–34.56)	2,806 (2,125–3,719)	25.6 (20.91–32.01)	−0.1 (−0.32–0.12)
Chile	14,469 (12,791–16,361)	129.08 (114.92–144.03)	31,679 (28,333–35,232)	136.35 (120.74–155.04)	0.32 (0.24–0.4)
China	335,614 (277,475–397,432)	34.89 (29.81–40.53)	717,078 (619,114–835,347)	40.51 (34.42–47.56)	0.22 (−0.06–0.51)
Colombia	14,259 (11,321–17,796)	53.05 (44.23–63.71)	18,374 (15,499–21,940)	35.24 (29.37–42.42)	−1.46 (−1.52 to −1.4)
Comoros	63 (50–79)	20.85 (17.65–24.37)	117 (97–141)	20.23 (17.2–23.66)	−0.07 (−0.22–0.08)
Congo	362 (289–436)	23.43 (19.93–27.11)	980 (757–1,323)	25.39 (20.62–32.44)	−0.43 (−1.35–0.5)
Cook Islands	4 (3–5)	27.05 (22.99–31.97)	6 (5–6)	25.8 (21.89–30.43)	−0.15 (−0.54–0.24)
Costa Rica	997 (798–1,249)	42.15 (35.39–50.91)	2,004 (1,707–2,376)	38.52 (32.3–46.34)	−0.31 (−0.33 to −0.29)
Croatia	5,024 (4,182–5,995)	95.86 (78.9–115.58)	6,469 (5,397–7,661)	88.15 (73.17–105.71)	−0.51 (−0.67 to −0.34)
Cuba	4,422 (3,688–5,287)	42.48 (35.81–50.55)	8,948 (7,559–10,646)	50.69 (42.41–60.53)	0.46 (0.39–0.53)
Cyprus	1,468 (1,302–1,646)	199.89 (176.09–224.17)	2,931 (2,566–3,340)	165.85 (144.28–190.27)	−0.62 (−0.76 to −0.49)
Czechia	14,184 (11,713–16,975)	118.73 (97.88–143.14)	12,393 (10,644–14,683)	76.83 (62.86–94.79)	−1.57 (−1.71 to −1.44)
CÃ’te d’Ivoire	1,586 (1,237–1,964)	22.28 (18.94–25.9)	3,903 (3,105–4,777)	22.71 (19.43–26.34)	−0.04 (−0.14–0.07)
Democratic People’s Republic of Korea	4,620 (3,892–5,385)	25.39 (21.77–29.1)	6,731 (5,840–7,688)	21.9 (18.86–25.07)	−0.39 (−0.44 to −0.35)
Democratic Republic of the Congo	5,610 (4,423–6,855)	21.99 (18.69–25.58)	14,776 (11,587–18,676)	24.73 (20.69–29.62)	−0.04 (−0.49–0.42)
Denmark	14,994 (12,889–17,352)	200.24 (172.56–228.79)	13,144 (11,333–15,129)	130.66 (112.68–149.53)	−1.61 (−1.7 to −1.53)
Djibouti	69 (50–98)	23.22 (18.73–29.75)	186 (152–228)	21.31 (18.08–24.98)	−0.43 (−0.59 to −0.27)
Dominica	15 (12–18)	22.54 (18.64–26.83)	18 (15–22)	24.98 (20.8–29.67)	0.51 (0.23–0.78)
Dominican Republic	1,350 (1,060–1,670)	23.6 (19.52–28.16)	3,157 (2,596–3,790)	29.44 (24.42–34.95)	0.76 (0.58–0.94)
Ecuador	2,870 (2,273–3,533)	36.7 (30.54–43.23)	6,544 (5,408–7,862)	37.63 (31.32–44.89)	0.06 (−0.08–0.21)
Egypt	16,425 (13,000–20,271)	37.17 (30.92–44.15)	27,347 (22,025–33,107)	32.16 (27.1–38.05)	−0.36 (−0.42 to −0.3)
El Salvador	3,597 (2,443–5,634)	74.69 (53.33–111.82)	3,367 (2,700–4,454)	52.78 (42.07–70.38)	−0.94 (−1.04 to −0.85)
Equatorial Guinea	64 (51–78)	22.07 (18.7–25.76)	180 (142–223)	20.45 (17.36–23.97)	−0.24 (−0.3 to −0.17)
Eritrea	3,911 (1,831–7,452)	117.88 (60.6–219.08)	2,015 (1,197–3,707)	49.34 (29.64–90.64)	−1.97 (−2.41 to −1.54)
Estonia	1,705 (1,425–2,032)	96.73 (79.59–117.3)	1,066 (907–1,250)	57.91 (47.42–70.8)	−2.05 (−2.18 to −1.91)
Eswatini	132 (104–162)	25.32 (21.4–29.24)	247 (197–308)	27.09 (22.74–32.29)	0.1 (−0.06–0.27)
Ethiopia	22,563 (13,160–39,921)	51.89 (35.51–81.97)	19,266 (13,816–29,417)	26.01 (20.61–35.37)	−1.5 (−1.95 to −1.05)
Fiji	106 (85–130)	18.94 (15.92–22.27)	146 (121–174)	18.17 (15.18–21.4)	−0.23 (−0.32 to −0.14)
Finland	15,650 (13,763–17,752)	244.19 (215.41–277.87)	22,391 (19,234–25,598)	206.82 (179.87–236.26)	−0.66 (−1.07 to −0.25)
France	175,491 (152,640–198,518)	232.5 (204.2–262.49)	241,869 (208,055–279,597)	189.63 (164.65–216.38)	−0.64 (−0.72 to −0.57)
Gabon	177 (143–210)	24.92 (21.31–28.94)	300 (248–361)	23.46 (20.16–27.41)	−0.27 (−0.3 to −0.24)
Gambia	120 (93–151)	19.53 (16.5–23.14)	297 (237–366)	20.93 (17.76–24.67)	0.16 (0.1–0.21)
Georgia	3,035 (2,455–3,731)	52.86 (42.35–65.34)	2,606 (2,206–3,088)	59.74 (48.54–73.29)	0.52 (0.25–0.79)
Germany	193,888 (168,918–219,019)	175.94 (154.64–199.55)	252,146 (215,841–290,403)	148.95 (129.52–169.87)	−0.62 (−0.68 to −0.56)
Ghana	1,848 (1,457–2,280)	19.66 (16.71–23.17)	4,711 (3,798–5,764)	21.03 (17.96–24.72)	0.14 (0.07–0.21)
Greece	23,780 (21,190–26,753)	182.75 (161.67–207.05)	21,902 (19,773–24,299)	123.98 (110.16–140.74)	−1.29 (−1.37 to −1.21)
Greenland	85 (75–98)	231.67 (204.21–262.44)	118 (103–135)	192.89 (168.82–220.26)	−0.69 (−0.77 to −0.61)
Grenada	24 (19–31)	29.95 (23.98–38.11)	33 (27–41)	30.96 (25.15–38.45)	0.05 (−0.04–0.15)
Guam	24 (20–30)	23 (19.46–27.1)	41 (35–48)	21.93 (18.44–25.98)	−0.12 (−0.2 to −0.05)
Guatemala	3,741 (2,675–5,427)	57.89 (45.16–79.03)	6,839 (5,434–8,611)	50.81 (41.4–62.68)	−0.36 (−0.4 to −0.33)
Guinea	908 (724–1,110)	20.1 (16.93–23.39)	1,902 (1,520–2,328)	21.63 (18.38–25.16)	0.25 (0.1–0.4)
Guinea-Bissau	165 (130–200)	25.48 (21.58–29.45)	299 (240–362)	24.45 (20.86–28.17)	−0.3 (−0.59 to −0.01)
Guyana	187 (148–228)	32.55 (27.38–38.09)	256 (211–308)	36.92 (31.15–43.73)	0.24 (0.12–0.36)
Haiti	1,492 (1,152–1,910)	30.07 (24.6–36.61)	4,840 (3,460–6,977)	46.11 (33.17–68.23)	2.06 (0.9–3.23)
Honduras	1,340 (1,021–1,709)	36.07 (29.57–43.23)	2,975 (2,419–3,629)	35.57 (29.91–42.29)	−0.28 (−0.68–0.13)
Hungary	14,989 (12,476–17,868)	119.8 (99.7–143.54)	11,370 (9,601–13,349)	74.14 (60.63–90.52)	−1.94 (−2.08 to −1.8)
Iceland	471 (415–536)	169.77 (149.37–193.61)	708 (623–810)	138.39 (120.68–159.06)	−0.69 (−0.74 to −0.64)
India	263,269 (212,011–320,262)	48.17 (40.48–57.23)	553,007 (457,896–665,198)	47.29 (39.73–56.78)	−0.16 (−0.2 to −0.12)
Indonesia	43,064 (34,322–53,538)	32.12 (27.17–38.03)	61,742 (51,253–73,553)	26.25 (22.25–30.89)	−0.79 (−0.91 to −0.68)
Iran (Islamic Republic of)	27,500 (21,170–35,065)	61.3 (50.23–74.78)	34,056 (28,479–40,797)	40.32 (33.83–47.8)	−1.3 (−1.4 to −1.2)
Iraq	12,189 (7,910–19,250)	80.11 (54.82–121.94)	23,539 (15,754–36,439)	68.1 (45.43–106.24)	0.14 (−0.28–0.57)
Ireland	5,834 (5,122–6,600)	154.37 (136.17–174.34)	9,044 (7,929–10,336)	132.73 (115.33–152.71)	−0.57 (−0.76 to −0.38)
Israel	5,868 (5,190–6,700)	123.91 (109.95–140.69)	12,530 (11,134–14,165)	111.86 (98.91–127.56)	−0.36 (−0.56 to −0.15)
Italy	177,900 (153,701–203,806)	231.49 (200.8–263.98)	188,226 (161,748–217,466)	157.76 (136.93–179.89)	−1.41 (−1.47 to −1.35)
Jamaica	529 (412–680)	24.51 (19.56–31.03)	784 (637–973)	25.86 (20.73–32.37)	0.02 (−0.12–0.15)
Japan	206,904 (185,589–230,722)	135.09 (120.44–152.15)	265,088 (233,387–297,820)	91.76 (81.22–104.03)	−1.4 (−1.54 to −1.26)
Jordan	857 (654–1,074)	31.53 (25.83–37.27)	2,697 (2,133–3,339)	25.55 (21.25–30.55)	−0.68 (−0.72 to −0.63)
Kazakhstan	8,217 (6,511–10,234)	53.46 (43.3–65.35)	9,043 (7,339–11,161)	48.33 (39.42–59.54)	−0.23 (−0.35 to −0.1)
Kenya	2,964 (2,317–3,717)	21.93 (18.62–25.51)	7,012 (5,672–8,510)	22.7 (19.41–26.43)	0.02 (−0.11–0.16)
Kiribati	10 (8–12)	17.73 (15.08–20.51)	15 (12–17)	15.28 (13.04–17.73)	−0.54 (−0.72 to −0.36)
Kuwait	871 (578–1,362)	56.97 (40.77–84.62)	1,581 (1,278–1,983)	34.21 (28.18–41.87)	−1.14 (−1.51 to −0.76)
Kyrgyzstan	1,883 (1,485–2,329)	47.93 (39.22–57.83)	2,030 (1,617–2,506)	32.68 (26.7–39.51)	−1.39 (−1.47 to −1.32)
Lao People’s Democratic Republic	969 (731–1,375)	30.06 (24.39–39.12)	1,383 (1,128–1,651)	23.61 (19.99–27.31)	−0.51 (−0.6 to −0.42)
Latvia	3,374 (2,815–3,967)	110.25 (90.78–131.91)	1,749 (1,509–2,051)	64.09 (52.97–77.94)	−2.35 (−2.57 to −2.13)
Lebanon	2,068 (1,252–3,870)	77.85 (49.31–142.47)	2,309 (1,652–3,738)	39.21 (27.77–63.79)	−2.06 (−2.32 to −1.8)
Lesotho	249 (201–297)	21.62 (18.21–24.98)	439 (361–523)	29.46 (25.17–33.93)	1.11 (0.92–1.29)
Liberia	1,197 (631–2,298)	50.08 (29.59–90.2)	953 (672–1,384)	26.8 (20.04–37.11)	−1.39 (−2.14 to −0.64)
Libya	1,275 (1,001–1,582)	40.04 (33.35–47.69)	3,175 (2,562–3,932)	50.49 (41.57–61.71)	1.63 (1.11–2.15)
Lithuania	3,994 (3,301–4,779)	98.05 (79.88–118.52)	2,922 (2,513–3,403)	69.71 (57.33–84.37)	−1.35 (−1.57 to −1.14)
Luxembourg	1,079 (953–1,208)	222.72 (196.31–250.92)	1,673 (1,445–1,908)	173.25 (149.86–198.84)	−0.84 (−0.87 to −0.8)
Madagascar	1,488 (1,153–1,886)	17.69 (14.82–20.89)	2,991 (2,326–3,766)	16.07 (13.47–19.15)	−0.4 (−0.45 to −0.35)
Malawi	1,166 (896–1,466)	18.09 (15.23–21.35)	2,144 (1,689–2,652)	18.23 (15.49–21.45)	−0.08 (−0.14 to −0.02)
Malaysia	3,361 (2,703–4,103)	26.2 (22.13–31.06)	7,939 (6,599–9,454)	27.07 (22.88–31.98)	0.09 (0.07–0.1)
Maldives	39 (30–49)	26.44 (22–31.99)	121 (98–150)	27.18 (22.58–32.43)	0.16 (−0.15–0.47)
Mali	1,510 (1,150–1,990)	24.9 (20.75–30.54)	4,074 (3,147–5,262)	27.05 (22.57–32.93)	0.05 (−0.47–0.57)
Malta	639 (559–730)	160.95 (140.4–184.39)	1,076 (951–1,236)	140.62 (123.34–161.85)	−0.37 (−0.5 to −0.23)
Marshall Islands	7 (5–8)	24.85 (21.02–28.72)	11 (9–13)	24.85 (21.19–28.95)	−0.01 (−0.06–0.04)
Mauritania	344 (276–412)	24.59 (20.85–28.45)	631 (510–768)	21.63 (18.49–25.26)	−0.49 (−0.53 to −0.45)
Mauritius	192 (153–237)	20.15 (16.69–24)	319 (273–378)	20.6 (17.14–24.78)	0.31 (0.21–0.41)
Mexico	42,109 (33,100–53,404)	65.08 (54.09–78.8)	55,081 (45,960–66,330)	43.16 (36.01–51.8)	−0.6 (−0.9 to −0.3)
Micronesia (Federated States of)	18 (15–22)	26.07 (22.26–30.01)	24 (19–28)	28.35 (24.18–32.8)	0.29 (0.06–0.51)
Monaco	68 (60–76)	126.97 (112.04–144.22)	89 (79–102)	121.38 (107.06–139.58)	−0.08 (−0.19–0.02)
Mongolia	770 (593–965)	44.66 (36.41–53.93)	1,561 (1,233–1,961)	50.9 (41.31–62.62)	0.52 (0.44–0.6)
Montenegro	491 (404–609)	78.45 (64.76–97.27)	499 (422–600)	66.86 (54.95–82.5)	−0.47 (−0.54 to −0.41)
Morocco	8,446 (6,668–10,317)	41.15 (34.13–48.74)	13,047 (10,794–15,462)	37.04 (30.93–43.72)	−0.43 (−0.5 to −0.37)
Mozambique	3,817 (2,370–6,633)	35.9 (24.23–59.34)	5,173 (3,871–7,185)	28.83 (22.54–39.85)	−0.56 (−0.65 to −0.46)
Myanmar	11,209 (8,710–14,724)	34.6 (28.42–42.86)	20,495 (16,192–26,799)	39.81 (31.96–50.88)	0.42 (−0.21–1.07)
Namibia	291 (222–388)	28.08 (22.84–35.47)	481 (393–584)	25.56 (21.52–30.34)	−0.46 (−0.59 to −0.34)
Nauru	2 (2–2)	29.01 (24.64–33.76)	3 (2–3)	32.05 (27.24–37.56)	0.23 (0.14–0.32)
Nepal	4,518 (3,511–5,707)	33.87 (28–40.34)	10,682 (8,587–13,070)	41.29 (34.34–49.35)	0.63 (0.29–0.96)
Netherlands	24,430 (21,559–27,724)	132.54 (116.87–149.93)	48,633 (41,266–56,684)	149.75 (128.42–174.03)	0.77 (0.27–1.28)
New Zealand	8,838 (7,784–10,089)	241.13 (211.58–276.39)	13,749 (12,100–15,647)	190.38 (166.19–218.42)	−0.62 (−0.69 to −0.56)
Nicaragua	1,849 (1,139–3,229)	56.53 (37.5–94.07)	2,432 (1,815–3,494)	43.52 (32.89–62.41)	−0.87 (−1.02 to −0.72)
Niger	1,170 (899–1,476)	22.99 (19.34–27.3)	3,573 (2,740–4,635)	23.68 (19.82–28.28)	0.14 (0.07–0.22)
Nigeria	13,536 (10,776–16,558)	21.65 (18.33–25.37)	31,218 (24,959–38,753)	21.87 (18.57–25.64)	0.02 (−0.05–0.1)
Niue	1 (0–1)	23.9 (20.3–28.07)	0 (0–1)	24.31 (20.54–28.41)	−0.05 (−0.33–0.23)
North Macedonia	1,118 (870–1,411)	57.14 (44.8–71.76)	1,429 (1,194–1,743)	57.65 (46.79–72.32)	0.06 (−0.11–0.22)
Northern Mariana Islands	12 (9–14)	36.01 (30.52–42.21)	17 (14–20)	36.02 (30.65–41.94)	−0.06 (−0.11 to −0.01)
Norway	12,900 (10,979–14,905)	207.7 (179.86–236.99)	15,236 (12,745–18,053)	158.83 (135.32–186.02)	−0.88 (−0.98 to −0.78)
Oman	899 (712–1,125)	67.31 (56.69–80)	1,822 (1,463–2,220)	51.75 (43.68–61.47)	−1.04 (−1.21 to −0.87)
Pakistan	15,746 (12,469–19,492)	19.6 (16.35–23.26)	36,097 (29,183–44,437)	20.16 (16.98–23.63)	0.28 (−0.04–0.59)
Palau	5 (4–6)	41.58 (35.06–49.3)	9 (7–10)	44.56 (37.37–52.93)	0.21 (0.18–0.24)
Palestine	942 (631–1,462)	56.27 (39.36–84.85)	2,201 (1,463–3,419)	54.67 (37.12–84.49)	0.28 (−0.43–0.99)
Panama	747 (592–948)	36.44 (30.08–44.59)	1,326 (1,083–1,611)	30.29 (24.64–36.92)	−0.69 (−0.73 to −0.65)
Papua New Guinea	690 (551–839)	27.27 (23.32–31.75)	2,288 (1,842–2,758)	34.26 (29.34–39.97)	0.44 (0.14–0.75)
Paraguay	1,247 (964–1,577)	37.66 (30.48–45.76)	2,558 (2,082–3,099)	38.15 (31.79–45.44)	0.05 (−0.02–0.13)
Peru	6,702 (5,008–9,216)	35.17 (27.87–45.56)	10,834 (8,825–13,159)	30.23 (24.77–36.57)	−0.33 (−0.42 to −0.24)
Philippines	16,103 (12,524–20,962)	33.22 (27.58–40.75)	24,452 (20,101–29,866)	25.35 (21.42–30.38)	−0.7 (−0.85 to −0.56)
Poland	35,483 (29,575–43,416)	88.41 (73.09–108.41)	38,201 (32,797–44,873)	69.79 (57.53–85.55)	−0.88 (−0.98 to −0.78)
Portugal	22,819 (20,537–25,495)	189.89 (169.98–214.18)	23,167 (20,755–26,060)	115.24 (102.37–131.37)	−1.85 (−1.93 to −1.77)
Puerto Rico	1,213 (1,017–1,444)	33.92 (28.5–40.34)	2,047 (1,789–2,382)	37.96 (31.75–45.48)	0.42 (0.32–0.53)
Qatar	183 (139–231)	48.93 (40.28–58.63)	1,186 (923–1,509)	44.01 (36.56–53.31)	−0.31 (−0.44 to −0.18)
Republic of Korea	82,660 (73,507–93,839)	229.07 (205.53–256.3)	129,472 (115,238–145,582)	158.05 (140.23–179.07)	−1.48 (−1.59 to −1.37)
Republic of Moldova	3,377 (2,788–4,073)	76.41 (63.33–91.68)	2,214 (1,880–2,606)	48.28 (39.7–58.79)	−1.68 (−1.75 to −1.6)
Romania	21,893 (17,955–26,725)	88.11 (71.3–108.7)	17,347 (14,838–20,219)	67.77 (54.88–83.6)	−0.99 (−1.05 to −0.93)
Russian Federation	141,406 (117,814–170,823)	86.99 (71.47–105.88)	132,879 (113,666–156,570)	72.49 (59.93–87.28)	−0.9 (−1.27 to −0.52)
Rwanda	1,868 (1,237–3,034)	32.51 (24.5–46.38)	4,091 (2,325–7,549)	46.37 (26.72–85.46)	−0.51 (−2.07–1.09)
Saint Kitts and Nevis	12 (9–14)	30.57 (25.01–37.51)	20 (16–24)	32.32 (26.56–39.97)	0.25 (0.14–0.37)
Saint Lucia	29 (23–36)	25.63 (21.04–31.12)	54 (45–64)	26.37 (21.67–32.2)	0.03 (−0.06–0.13)
Saint Vincent and the Grenadines	23 (19–29)	25.39 (20.96–30.71)	36 (31–44)	29.08 (24.02–35.25)	0.36 (0.26–0.47)
Samoa	30 (24–37)	24.8 (20.9–29.11)	44 (36–51)	26.47 (22.64–30.82)	0.5 (0–0.99)
San Marino	42 (38–47)	138.37 (122.9–156.44)	81 (71–91)	130.19 (114.71–148.23)	−0.12 (−0.2 to −0.03)
Sao Tome and Principe	21 (17–26)	23 (19.05–27.44)	47 (37–58)	31.16 (26.15–37.5)	0.86 (0.82–0.89)
Saudi Arabia	8,603 (6,580–10,855)	79.77 (66.38–95.17)	32,964 (26,007–41,146)	97.95 (81.98–117.87)	0.82 (0.75–0.88)
Senegal	964 (747–1,208)	18.94 (15.93–22.45)	2,065 (1,654–2,534)	19.88 (16.87–23.33)	0.07 (0–0.15)
Serbia	6,487 (5,324–7,919)	64.91 (52.81–80.18)	7,171 (6,116–8,485)	59.95 (48.93–74.13)	−0.43 (−0.69 to −0.16)
Seychelles	16 (13–20)	25.56 (21.25–30.64)	26 (22–31)	23.26 (19.59–27.88)	−0.35 (−0.42 to −0.28)
Sierra Leone	583 (458–715)	19.02 (15.94–22.47)	1,413 (1,087–1,845)	24.23 (19.5–31.24)	−0.78 (−1.51 to −0.04)
Singapore	3,188 (2,812–3,626)	116.28 (103.52–129.91)	7,172 (6,444–8,022)	95.71 (84.97–109.46)	−0.75 (−0.91 to −0.58)
Slovakia	5,710 (4,721–6,910)	102.51 (84.41–124.46)	6,220 (5,299–7,408)	84.18 (68.94–102.98)	−0.57 (−0.65 to −0.49)
Slovenia	2,862 (2,396–3,466)	128.08 (106.09–156.71)	3,546 (2,997–4,182)	98.39 (81.48–119.99)	−0.47 (−0.69 to −0.25)
Solomon Islands	73 (58–88)	38.1 (32.35–44.28)	196 (160–238)	45.12 (38.08–53.48)	0.55 (0.47–0.63)
Somalia	2,016 (1,221–3,509)	32.58 (23.03–51.47)	4,237 (3,017–6,187)	29.68 (22.89–40.73)	0.18 (−0.27–0.64)
South Africa	11,903 (9,680–14,305)	40.1 (33.84–46.72)	14,397 (12,046–17,007)	26.52 (22.54–30.88)	−1.54 (−1.68 to −1.4)
South Sudan	930 (718–1,212)	21.82 (18.05–26.76)	1,915 (1,381–2,732)	27.6 (21.02–37.99)	0.81 (0.17–1.45)
Spain	72,710 (65,044–80,851)	154.3 (137.3–173.8)	102,306 (90,603–116,483)	127.72 (112.39–146.61)	−0.7 (−0.81 to −0.58)
Sri Lanka	8,633 (5,846–13,251)	54.5 (39.27–78.32)	12,052 (9,565–15,746)	50.02 (39.58–65.07)	−0.07 (−0.61–0.48)
Sudan	7,059 (5,152–10,152)	42.63 (33.2–56.71)	11,485 (8,994–14,665)	34.92 (28.37–43.09)	−0.42 (−0.66 to −0.19)
Suriname	87 (68–116)	25.68 (21.03–32.58)	161 (134–197)	26.74 (22.22–32.79)	0.18 (0.11–0.25)
Sweden	22,760 (19,340–26,295)	173.36 (150.54–198.03)	27,589 (23,116–32,589)	139.29 (119.17–160.55)	−0.67 (−0.8 to −0.55)
Switzerland	25,926 (22,349–29,792)	269.89 (233.28–308.29)	31,038 (26,598–35,677)	185.95 (160.13–215.46)	−1.35 (−1.4 to −1.3)
Syrian Arab Republic	2,899 (2,223–3,866)	29.97 (24.36–37.24)	8,595 (5,153–14,700)	66.8 (39.29–115.7)	4.79 (3.53–6.06)
Taiwan (Province of China)	7,255 (6,034–8,636)	40.84 (34.75–47.91)	8,411 (7,300–9,818)	23.32 (19.94–27.46)	−2.24 (−2.42 to −2.06)
Tajikistan	1,754 (1,342–2,206)	39.57 (32.09–47.92)	2,907 (2,237–3,776)	33.42 (26.47–41.95)	−1.83 (−2.43 to −1.23)
Thailand	17,644 (14,424–21,081)	36.95 (31.4–43.14)	29,705 (25,708–34,360)	33.44 (28.24–39.25)	−0.56 (−0.67 to −0.46)
Timor-Leste	387 (221–665)	54.73 (34.47–89.56)	433 (287–697)	43.28 (28.38–70.77)	−1.45 (−2.06 to −0.84)
Togo	486 (378–602)	21.68 (18.46–25.29)	1,189 (959–1,434)	21.89 (18.65–25.41)	−0.05 (−0.14–0.03)
Tokelau	0 (0–0)	21.63 (18.29–25.39)	0 (0–0)	22.01 (18.66–25.84)	0.02 (−0.03–0.07)
Tonga	14 (11–17)	19.9 (16.89–23.47)	15 (13–18)	17.42 (14.72–20.42)	−0.37 (−0.52 to −0.23)
Trinidad and Tobago	283 (226–350)	27.14 (22.58–32.78)	433 (366–511)	27.54 (22.73–33.48)	0.34 (0.22–0.47)
Tunisia	2,610 (2,038–3,229)	37.64 (30.95–45.34)	4,323 (3,606–5,165)	34.64 (28.69–41.5)	−0.27 (−0.33 to −0.22)
Turkey	14,212 (11,076–17,678)	29.22 (23.81–35.23)	27,124 (22,650–32,204)	31.32 (26.08–37.27)	0.3 (0.19–0.42)
Turkmenistan	1,132 (861–1,438)	35.35 (28.34–43.44)	1,408 (1,113–1,774)	28.65 (22.93–35.64)	−0.82 (−0.95 to −0.69)
Tuvalu	2 (2–2)	25.19 (21.44–29.09)	3 (2–3)	25.88 (22.01–30.12)	−0.35 (−0.53 to −0.17)
Uganda	3,769 (2,501–6,007)	29.8 (21.52–44.42)	6,141 (4,724–8,100)	25.1 (20.02–33.32)	−0.66 (−0.81 to −0.52)
Ukraine	52,970 (44,043–63,616)	89.19 (72.76–109.57)	40,759 (34,763–47,756)	73.52 (60.54–88.92)	−1.12 (−1.33 to −0.92)
United Arab Emirates	699 (536–886)	49.56 (41.38–59.07)	4,740 (3,803–5,855)	46.53 (38.6–55.61)	−0.25 (−0.29 to −0.21)
United Kingdom	104,736 (91,824–118,036)	136.43 (120.1–154.75)	143,144 (123,728–164,541)	126.34 (109.59–144.26)	−0.19 (−0.32 to −0.06)
United Republic of Tanzania	3,431 (2,675–4,257)	19.74 (16.64–23.18)	7,548 (6,006–9,272)	19.74 (16.86–23.07)	−0.03 (−0.06–0)
United States of America	471,514 (417,848–527,280)	158.01 (139.91–177.31)	851,924 (740,753–968,614)	159.34 (140.01–180.18)	0 (−0.09–0.08)
United States Virgin Islands	29 (24–35)	30.27 (25.31–35.95)	38 (33–44)	29.35 (24.67–35.25)	−0.29 (−0.43 to −0.14)
Uruguay	4,529 (4,034–5,126)	129.36 (114.41–147.67)	5,951 (5,370–6,593)	125.33 (111.46–141.6)	−0.17 (−0.2 to −0.14)
Uzbekistan	6,733 (5,182–8,497)	37.49 (30.17–45.41)	10,407 (8,298–12,882)	32.59 (26.46–39.72)	−0.53 (−0.66 to −0.4)
Vanuatu	22 (17–26)	21.61 (18.34–25.19)	50 (41–60)	21.91 (18.55–25.41)	−0.04 (−0.22–0.13)
Venezuela (Bolivarian Republic of)	6,926 (5,397–8,791)	45.03 (36.99–54.58)	11,260 (9,451–13,394)	40.64 (33.65–48.85)	−0.45 (−0.69 to −0.21)
Viet Nam	15,446 (12,614–18,965)	31.57 (26.96–37.57)	37,312 (31,866–44,103)	39.96 (34.27–47.35)	0.91 (0.81–1.02)
Yemen	3,735 (2,888–4,646)	39.74 (33.22–46.81)	16,622 (11,115–26,330)	57.23 (41.8–83.17)	1.27 (0.81–1.73)
Zambia	1,027 (795–1,274)	20.7 (17.59–24.17)	2,514 (1,989–3,096)	22.39 (19.03–26.04)	0.2 (0.14–0.26)
Zimbabwe	1,451 (1,153–1,759)	22.95 (19.64–26.61)	2,177 (1,752–2,616)	21.33 (18.22–24.5)	−0.35 (−0.44 to −0.26)

**Table 3:** The number of YLDs cases and the age-standardized YLDs rate attributable to fracture of vertebral column in 1990 and 2021, and its trends from 1990 to 2021 globally.

	Number of YLDs cases (95% UI) in 1990	The age-standardized YLDs rate/100,000 (95% UI) in 1990	Number of YLDs cases (95% UI) in 2021	The age-standardized YLDs rate/100,000 (95% UI) in 2021	EAPC (95% CI)
Global	352,960 (235,711–491,606)	8.33 (5.61–11.49)	545,923 (366,571–757,099)	6.62 (4.43–9.2)	−0.81 (−0.85 to −0.78)
Sex					
Female	159,836 (105,668–222,017)	7.33 (4.89–10.07)	267,663 (177,477–370,783)	5.98 (3.95–8.31)	−0.71 (−0.75 to −0.68)
Male	193,123 (129,020–266,630)	9.07 (6.17–12.4)	278,260 (188,992–385,121)	7.14 (4.83–9.87)	−0.85 (−0.89 to −0.81)
Age					
<5 years	9,769 (5,489–15,931)	1.58 (0.89–2.57)	5,896 (3,232–9,498)	0.9 (0.49–1.44)	−1.93 (−2.1 to −1.75)
5–9 years	10,347 (5,911–16,854)	1.77 (1.01–2.89)	8,263 (4,768–13,638)	1.2 (0.69–1.99)	−1.16 (−1.25 to −1.07)
10–14 years	11,192 (6,414–17,819)	2.09 (1.2–3.33)	10,002 (5,654–16,014)	1.5 (0.85–2.4)	−1.14 (−1.21 to −1.07)
15–19 years	16,521 (9,736–25,600)	3.18 (1.87–4.93)	14,010 (7,951–21,724)	2.25 (1.27–3.48)	−1.13 (−1.23 to −1.04)
20–24 years	19,661 (11,833–30,511)	4 (2.4–6.2)	16,822 (9,972–26,278)	2.82 (1.67–4.4)	−1.24 (−1.31 to −1.18)
25–29 years	19,853 (12,380–29,577)	4.49 (2.8–6.68)	18,317 (11,466–27,364)	3.11 (1.95–4.65)	−1.26 (−1.32 to −1.21)
30–34 years	19,851 (12,702–29,184)	5.15 (3.3–7.57)	21,509 (13,674–32,075)	3.56 (2.26–5.31)	−1.34 (−1.38 to −1.29)
35–39 years	20,444 (13,364–30,279)	5.8 (3.79–8.6)	23,322 (15,386–34,743)	4.16 (2.74–6.19)	−1.31 (−1.4 to −1.22)
40–44 years	19,811 (13,276–28,143)	6.92 (4.63–9.82)	24,041 (16,381–33,996)	4.81 (3.27–6.8)	−1.34 (−1.42 to −1.26)
45–49 years	18,615 (12,714–25,994)	8.02 (5.48–11.19)	27,085 (18,029–37,521)	5.72 (3.81–7.92)	−1.25 (−1.33 to −1.17)
50–54 years	20,899 (13,954–29,102)	9.83 (6.56–13.69)	32,010 (21,858–44,262)	7.19 (4.91–9.95)	−1.11 (−1.18 to −1.03)
55–59 years	22,509 (15,294–31,008)	12.15 (8.26–16.74)	37,536 (25,714–51,310)	9.49 (6.5–12.97)	−0.85 (−0.91 to −0.79)
60–64 years	24,835 (16,993–34,099)	15.46 (10.58–21.23)	40,863 (28,056–57,004)	12.77 (8.77–17.81)	−0.68 (−0.73 to −0.63)
65–69 years	25,307 (17,235–34,619)	20.47 (13.94–28.01)	46,206 (31,176–64,452)	16.75 (11.3–23.37)	−0.58 (−0.61 to −0.54)
70–74 years	22,525 (14,941–30,797)	26.61 (17.65–36.38)	49,016 (32,528–67,854)	23.81 (15.8–32.96)	−0.51 (−0.57 to −0.45)
75–79 years	25,227 (16,599–35,624)	40.98 (26.97–57.87)	45,959 (30,227–65,707)	34.85 (22.92–49.82)	−0.55 (−0.63 to −0.46)
80–84 years	22,527 (14,408–32,111)	63.68 (40.73–90.77)	48,230 (30,609–69,795)	55.07 (34.95–79.69)	−0.53 (−0.58 to −0.48)
85–89 years	14,757 (9,467–21,019)	97.66 (62.65–139.1)	40,590 (25,871–57,988)	88.78 (56.58–126.83)	−0.34 (−0.43 to −0.24)
90–94 years	6,283 (3,997–8,830)	146.63 (93.28–206.06)	24,956 (15,755–35,243)	139.5 (88.07–197)	−0.12 (−0.22 to −0.02)
95 + years	2,027 (1,277–2,831)	199.1 (125.39–278.06)	11,290 (6,963–16,115)	207.14 (127.75–295.67)	0.05 (−0.08–0.17)
SDI region					
High-middle SDI	85,219 (56,768–118,388)	8.56 (5.75–11.85)	111,095 (75,248–154,768)	6.63 (4.46–9.32)	−1 (−1.08 to −0.92)
High SDI	163,472 (110,634–224,553)	16 (10.84–22.04)	245,574 (163,359–339,966)	13.36 (8.95–18.51)	−0.64 (−0.66 to −0.62)
Low-middle SDI	33,576 (21,908–48,367)	3.98 (2.72–5.63)	59,226 (40,462–84,059)	3.76 (2.56–5.27)	−0.28 (−0.35 to −0.21)
Low SDI	12,950 (8,228–19,543)	3.52 (2.38–5.08)	26,118 (17,629–38,250)	3.44 (2.39–4.87)	−0.06 (−0.17–0.05)
Middle SDI	57,381 (38,094–82,998)	4.14 (2.78–5.85)	103,474 (69,771–145,983)	4.09 (2.76–5.74)	−0.08 (−0.17–0.01)
GBD region					
Advanced Health System	224,192 (151,792–307,414)	14.98 (10.14–20.65)	307,694 (204,754–424,612)	12.51 (8.41–17.31)	−0.68 (−0.73 to −0.63)
Africa	15,915 (10,176–23,519)	3.27 (2.22–4.58)	27,036 (18,058–38,436)	2.76 (1.89–3.8)	−0.55 (−0.65 to −0.45)
African Region	11,828 (7,532–17,716)	3.04 (2.06–4.3)	20,268 (13,409–29,060)	2.56 (1.76–3.52)	−0.62 (−0.75 to −0.48)
America	76,466 (51,688–106,595)	11.82 (8.08–16.48)	130,410 (87,374–177,679)	10.31 (6.89–14.09)	−0.4 (−0.44 to −0.37)
Andean Latin America	1,204 (779–1,787)	3.74 (2.49–5.44)	2,181 (1,450–3,098)	3.41 (2.28–4.81)	−0.23 (−0.28 to −0.17)
Asia	126,263 (84,944–178,127)	5.26 (3.53–7.31)	231,565 (155,889–322,539)	4.87 (3.26–6.79)	−0.37 (−0.42 to −0.32)
Australasia	4,411 (3,010–6,105)	20.19 (13.77–27.95)	8,616 (5,703–11,844)	18.56 (12.45–25.78)	−0.13 (−0.21 to −0.06)
Basic Health System	81,840 (54,533–117,057)	4.31 (2.89–6.06)	143,506 (96,867–202,641)	4.21 (2.83–5.94)	−0.1 (−0.19 to −0.01)
Caribbean	1,087 (716–1,568)	3.58 (2.37–5.08)	2,262 (1,567–3,155)	4.42 (3.04–6.15)	0.76 (0.32–1.21)
Central Africa	1,081 (707–1,581)	2.34 (1.59–3.31)	3,105 (2,081–4,515)	2.74 (1.91–3.87)	−0.06 (−0.48–0.36)
Central Asia	3,007 (1,934–4,374)	4.84 (3.19–6.87)	3,667 (2,411–5,236)	3.98 (2.64–5.66)	−0.74 (−0.85 to −0.63)
Central Europe	12,855 (8,390–18,273)	9.73 (6.33–13.86)	11,981 (8,062–16,770)	7.44 (4.81–10.66)	−1.04 (−1.1 to −0.97)
Central Latin America	8,072 (5,265–11,970)	6.1 (4.09–8.64)	10,874 (7,365–15,275)	4.29 (2.9–6.02)	−0.76 (−0.93 to −0.6)
Central Sub-Saharan Africa	1,056 (695–1,555)	2.63 (1.8–3.73)	2,477 (1,667–3,610)	2.69 (1.87–3.82)	−0.38 (−0.75–0)
Commonwealth High Income	20,518 (13,943–28,534)	15.28 (10.41–21.17)	33,773 (22,282–46,767)	14.27 (9.51–19.68)	−0.12 (−0.22 to −0.03)
Commonwealth Low Income	3,283 (2,112–4,830)	2.05 (1.39–2.9)	6,255 (4,324–8,833)	2.01 (1.44–2.77)	−0.69 (−1.05 to −0.33)
Commonwealth Middle Income	34,207 (22,583–49,259)	4.24 (2.88–5.98)	69,678 (46,924–99,198)	4.24 (2.84–5.97)	−0.06 (−0.11 to −0.01)
East Asia	37,017 (24,397–53,145)	3.64 (2.42–5.12)	75,654 (50,869–106,460)	4.13 (2.73–5.86)	0.14 (−0.14–0.42)
East Asia & Pacific—WB	83,926 (56,212–117,094)	5.64 (3.82–7.84)	144,966 (98,120–200,907)	4.98 (3.34–6.94)	−0.59 (−0.68 to −0.5)
Eastern Africa	5,440 (3,162–9,177)	3.76 (2.44–5.92)	7,169 (4,691–10,573)	2.71 (1.87–3.81)	−0.79 (−1.02 to −0.57)
Eastern Europe	22,600 (14,963–32,230)	9.2 (6.02–13.16)	19,785 (13,361–27,689)	7.6 (4.98–10.83)	−0.96 (−1.25 to −0.66)
Eastern Mediterranean Region	13,110 (8,620–19,245)	4.37 (2.99–6.16)	27,300 (18,441–39,371)	4.24 (2.9–6.03)	0.16 (0.07–0.25)
Eastern Sub-Saharan Africa	5,380 (3,158–8,962)	3.47 (2.27–5.43)	7,361 (4,815–10,978)	2.64 (1.82–3.73)	−0.73 (−0.97 to −0.49)
Europe	133,817 (90,211–183,394)	14.48 (9.76–20)	156,254 (104,086–216,800)	11.65 (7.84–16.17)	−0.81 (−0.9 to −0.72)
Europe & Central Asia—WB	135,757 (91,502–186,293)	14.14 (9.53–19.54)	158,751 (105,674–220,145)	11.29 (7.59–15.69)	−0.84 (−0.93 to −0.75)
European Region	136,542 (92,016–187,390)	14.12 (9.52–19.51)	160,277 (106,714–222,204)	11.28 (7.59–15.68)	−0.83 (−0.92 to −0.74)
High-income Asia Pacific	30,362 (20,667–41,651)	15.85 (10.79–21.83)	40,341 (26,946–55,606)	11.06 (7.51–15.2)	−1.37 (−1.48 to −1.25)
High-income North America	52,725 (35,750–73,137)	16.08 (10.96–22.25)	93,496 (62,165–128,945)	15.76 (10.54–21.65)	−0.08 (−0.15–0)
Latin America & Caribbean—WB	23,883 (15,785–34,172)	6.71 (4.49–9.4)	37,140 (24,942–51,757)	5.39 (3.63–7.54)	−0.58 (−0.68 to −0.48)
Limited Health System	43,114 (28,220–62,462)	3.96 (2.69–5.62)	85,960 (58,813–122,448)	3.94 (2.71–5.54)	−0.09 (−0.15 to −0.03)
Middle East & North Africa—WB	11,305 (7,530–16,536)	5.62 (3.89–7.94)	21,973 (15,110–31,459)	5.13 (3.55–7.26)	−0.03 (−0.12–0.05)
Minimal Health System	3,452 (2,174–5,570)	3.3 (2.17–5.2)	8,327 (5,377–12,827)	3.5 (2.37–5.12)	−0.07 (−0.29–0.14)
North Africa and Middle East	13,683 (9,017–20,074)	4.91 (3.37–6.95)	27,310 (18,500–39,366)	4.8 (3.3–6.87)	0.15 (0.06–0.24)
North America	52,717 (35,744–73,126)	16.07 (10.96–22.24)	93,485 (62,157–128,930)	15.76 (10.54–21.65)	−0.08 (−0.15–0)
Northern Africa	4,082 (2,646–6,047)	4.19 (2.79–6.06)	6,629 (4,372–9,436)	3.61 (2.42–5.1)	−0.47 (−0.5 to −0.43)
Oceania	118 (77–170)	2.68 (1.77–3.79)	321 (212–459)	3.24 (2.16–4.53)	0.38 (0.12–0.63)
Region of the Americas	76,466 (51,688–106,595)	11.82 (8.08–16.48)	130,410 (87,374–177,679)	10.31 (6.89–14.09)	−0.4 (−0.44 to −0.37)
South-East Asia Region	38,809 (25,334–55,929)	4.25 (2.89–6.06)	74,545 (50,244–106,068)	4.1 (2.76–5.79)	−0.25 (−0.32 to −0.18)
South Asia	31,330 (20,482–45,253)	4.24 (2.88–6.01)	64,644 (43,185–92,519)	4.26 (2.85–6)	−0.11 (−0.17 to −0.04)
South Asia—WB	33,029 (21,711–47,562)	4.3 (2.93–6.13)	68,567 (46,817–98,058)	4.37 (2.98–6.14)	−0.08 (−0.14 to −0.01)
Southeast Asia	12,920 (8,419–18,829)	3.57 (2.42–5.05)	21,193 (14,577–29,974)	3.22 (2.22–4.53)	−0.35 (−0.47 to −0.23)
Southern Africa	2,497 (1,654–3,591)	3.46 (2.39–4.82)	3,561 (2,415–4,997)	2.6 (1.81–3.54)	−1.09 (−1.22 to −0.97)
Southern Latin America	5,597 (3,787–7,747)	11.95 (8.09–16.52)	9,371 (6,339–12,904)	11.82 (7.96–16.34)	−0.03 (−0.11–0.05)
Southern Sub-Saharan Africa	1,518 (998–2,148)	3.75 (2.55–5.2)	1,925 (1,290–2,677)	2.66 (1.83–3.68)	−1.28 (−1.41 to −1.14)
Sub-Saharan Africa—WB	11,862 (7,569–18,153)	3.02 (2.04–4.34)	20,454 (13,524–29,444)	2.56 (1.76–3.53)	−0.55 (−0.68 to −0.42)
Tropical Latin America	7,961 (5,054–11,558)	6.25 (4.09–9)	12,532 (8,310–17,771)	5.12 (3.39–7.31)	−0.63 (−0.72 to −0.53)
Western Africa	2,816 (1,816–4,089)	2.29 (1.54–3.2)	6,572 (4,328–9,388)	2.33 (1.59–3.26)	0.01 (−0.07–0.08)
Western Europe	96,921 (65,078–133,350)	19.29 (13.06–26.44)	122,477 (81,287–171,373)	15.45 (10.37–21.43)	−0.76 (−0.81 to −0.72)
Western Pacific Region	74,923 (50,181–104,400)	6.02 (4.08–8.35)	131,588 (88,934–182,699)	5.33 (3.57–7.45)	−0.58 (−0.66 to −0.51)
Western Sub-Saharan Africa	3,136 (2,037–4,540)	2.3 (1.55–3.21)	7,456 (4,895–10,680)	2.35 (1.6–3.3)	0.02 (−0.05–0.08)
World Bank High Income	199,929 (135,090–274,705)	16.59 (11.23–22.87)	284,876 (189,482–394,079)	13.69 (9.19–18.96)	−0.69 (−0.71 to −0.67)
World Bank Low Income	9,002 (5,558–14,295)	3.6 (2.37–5.41)	16,952 (11,172–25,754)	3.3 (2.26–4.77)	−0.11 (−0.29–0.07)
World Bank Lower Middle Income	60,484 (39,815–87,720)	4.16 (2.84–5.89)	108,093 (73,502–153,970)	3.87 (2.64–5.43)	−0.34 (−0.4 to −0.28)
World Bank Upper Middle Income	83,183 (55,141–118,826)	4.8 (3.22–6.76)	135,565 (90,923–190,266)	4.59 (3.07–6.48)	−0.27 (−0.35 to −0.19)
Country					
Afghanistan	763 (358–1,773)	9.45 (4.36–22.14)	2,677 (1,410–4,827)	10.9 (5.73–19.1)	0.04 (−0.28–0.35)
Albania	213 (129–335)	7.08 (4.43–10.84)	197 (127–285)	6.38 (4.02–9.44)	−0.65 (−0.88 to −0.42)
Algeria	947 (599–1,396)	4.74 (3.12–6.88)	1,494 (975–2,123)	3.65 (2.42–5.16)	−1.09 (−1.19 to −0.99)
American Samoa	1 (1–1)	2.69 (1.76–3.88)	1 (1–2)	2.77 (1.88–3.89)	0.32 (−0.13–0.76)
Andorra	13 (8–18)	23 (15.28–31.97)	35 (22–48)	24.33 (16.05–33.65)	0.17 (0.13–0.22)
Angola	342 (212–563)	4.02 (2.61–6.18)	589 (402–849)	2.98 (2.07–4.19)	−1.36 (−1.82 to −0.89)
Antigua and Barbuda	2 (1–3)	2.9 (1.84–4.34)	3 (2–4)	3.09 (2–4.68)	0.06 (−0.2–0.31)
Argentina	3,624 (2,457–5,080)	11.36 (7.71–15.98)	5,546 (3,708–7,738)	10.76 (7.18–15.1)	−0.21 (−0.32 to −0.11)
Armenia	218 (148–314)	7.01 (4.73–10.11)	130 (91–182)	3.81 (2.64–5.44)	−1.9 (−2.53 to −1.27)
Australia	3,511 (2,402–4,886)	19.31 (13.14–26.85)	7,233 (4,782–9,999)	18.36 (12.32–25.4)	−0.02 (−0.1–0.06)
Austria	2,396 (1,599–3,307)	23.39 (15.72–32.17)	2,684 (1,797–3,734)	17.23 (11.59–24.24)	−0.87 (−0.94 to −0.8)
Azerbaijan	258 (166–376)	3.8 (2.49–5.49)	344 (224–504)	3.22 (2.12–4.74)	−0.81 (−1.2 to −0.41)
Bahamas	6 (4–9)	2.83 (1.84–4.06)	12 (8–17)	3.03 (2.06–4.31)	0.37 (0.12–0.62)
Bahrain	13 (8–19)	3.22 (2.09–4.61)	42 (28–60)	2.91 (1.93–4.15)	−0.62 (−0.77 to −0.47)
Bangladesh	1,404 (897–2,027)	1.62 (1.07–2.32)	2,699 (1,806–3,818)	1.73 (1.17–2.43)	−0.58 (−1.21–0.06)
Barbados	6 (4–9)	2.31 (1.49–3.34)	9 (6–13)	2.47 (1.61–3.56)	0.07 (−0.07–0.21)
Belarus	884 (579–1,242)	7.81 (5.1–11.1)	942 (621–1,328)	7.86 (5.07–11.41)	−0.1 (−0.44–0.24)
Belgium	2,858 (1,950–3,958)	21.66 (14.75–29.88)	4,434 (2,910–6,142)	21.34 (14.16–29.61)	0.07 (−0.14–0.28)
Belize	5 (3–7)	2.88 (1.89–4.23)	13 (9–20)	3.43 (2.26–4.96)	0.33 (0.16–0.49)
Benin	74 (48–108)	2.22 (1.48–3.15)	194 (125–287)	2.24 (1.5–3.18)	−0.05 (−0.1–0)
Bermuda	2 (1–2)	2.74 (1.8–4.02)	3 (2–4)	2.7 (1.75–3.92)	−0.11 (−0.2 to −0.01)
Bhutan	10 (6–15)	2.46 (1.62–3.55)	24 (16–34)	3.65 (2.41–5.17)	1.17 (0.92–1.41)
Bolivia (Plurinational State of)	174 (111–255)	3.53 (2.31–5.06)	340 (223–489)	3.26 (2.18–4.65)	−0.29 (−0.32 to −0.27)
Bosnia and Herzegovina	334 (213–499)	7.55 (4.9–11.21)	277 (190–392)	6.55 (4.39–9.55)	−1.27 (−1.75 to −0.79)
Botswana	21 (14–31)	2.32 (1.52–3.32)	53 (34–77)	2.6 (1.72–3.71)	0.22 (0.02–0.42)
Brazil	7,827 (4,967–11,360)	6.31 (4.13–9.08)	12,260 (8,141–17,376)	5.15 (3.42–7.35)	−0.64 (−0.73 to −0.54)
Brunei Darussalam	31 (20–43)	18.08 (12.2–24.35)	55 (37–77)	13.87 (9.4–19.04)	−0.89 (−0.94 to −0.83)
Bulgaria	840 (564–1,197)	8.74 (5.74–12.66)	626 (411–891)	7.15 (4.55–10.54)	−0.72 (−0.75 to −0.68)
Burkina Faso	152 (98–220)	2.41 (1.6–3.42)	404 (259–591)	2.63 (1.8–3.72)	0.22 (0.11–0.34)
Burundi	84 (53–122)	2.24 (1.46–3.19)	401 (230–695)	4.53 (2.63–7.72)	0.21 (−1.34–1.78)
Cabo Verde	7 (4–9)	2.27 (1.52–3.18)	13 (8–18)	2.5 (1.65–3.53)	0.38 (0.35–0.42)
Cambodia	382 (219–682)	4.77 (2.92–8.25)	623 (407–927)	4.51 (3.01–6.61)	−0.22 (−0.32 to −0.13)
Cameroon	150 (98–219)	2.23 (1.52–3.15)	510 (331–739)	2.45 (1.64–3.48)	0.28 (0.22–0.34)
Canada	4,828 (3,292–6,742)	15.58 (10.68–21.8)	9,591 (6,354–13,130)	14.29 (9.43–19.46)	−0.12 (−0.19 to −0.05)
Central African Republic	46 (29–66)	2.44 (1.63–3.44)	153 (95–247)	3.41 (2.24–5.09)	1.19 (0.8–1.59)
Chad	131 (84–201)	2.74 (1.82–4.02)	301 (194–449)	2.66 (1.79–3.76)	−0.13 (−0.36–0.1)
Chile	1,506 (1,013–2,092)	13.3 (8.96–18.11)	3,221 (2,200–4,400)	13.98 (9.58–19.24)	0.3 (0.22–0.38)
China	35,750 (23,555–51,348)	3.65 (2.42–5.12)	74,079 (49,798–104,323)	4.19 (2.78–5.96)	0.19 (−0.1–0.47)
Colombia	1,527 (997–2,208)	5.58 (3.72–7.82)	1,929 (1,313–2,744)	3.71 (2.51–5.31)	−1.46 (−1.52 to −1.4)
Comoros	7 (4–10)	2.18 (1.48–3.15)	12 (8–18)	2.11 (1.4–3.03)	−0.07 (−0.22–0.09)
Congo	39 (25–57)	2.44 (1.62–3.45)	103 (70–150)	2.61 (1.82–3.71)	−0.5 (−1.45–0.46)
Cook Islands	0 (0–1)	2.85 (1.88–4.12)	1 (0–1)	2.68 (1.79–3.83)	−0.18 (−0.59–0.23)
Costa Rica	106 (68–154)	4.39 (2.86–6.25)	208 (137–294)	4.02 (2.65–5.69)	−0.31 (−0.33 to −0.29)
Croatia	524 (343–737)	9.99 (6.5–14.18)	648 (425–904)	9.04 (6.01–12.73)	−0.57 (−0.74 to −0.4)
Cuba	465 (308–670)	4.43 (2.93–6.33)	902 (579–1,262)	5.21 (3.37–7.45)	0.42 (0.35–0.48)
Cyprus	152 (104–212)	20.41 (13.88–28.3)	298 (198–413)	16.92 (11.21–23.52)	−0.62 (−0.76 to −0.49)
Czechia	1,450 (948–2,074)	12.19 (7.9–17.44)	1,256 (812–1,762)	7.98 (5.14–11.4)	−1.52 (−1.66 to −1.39)
CÃ’te d’Ivoire	171 (106–253)	2.31 (1.52–3.25)	419 (273–605)	2.36 (1.6–3.34)	−0.03 (−0.13–0.08)
Democratic People’s Republic of Korea	494 (314–711)	2.68 (1.73–3.79)	709 (472–993)	2.31 (1.53–3.24)	−0.39 (−0.43 to −0.34)
Democratic Republic of the Congo	604 (387–892)	2.28 (1.52–3.23)	1,581 (1,047–2,328)	2.57 (1.75–3.69)	−0.07 (−0.54–0.41)
Denmark	1,503 (989–2,082)	20.3 (13.42–27.85)	1,319 (865–1,858)	13.38 (8.92–18.91)	−1.57 (−1.65 to −1.48)
Djibouti	8 (5–12)	2.45 (1.58–3.58)	20 (13–28)	2.22 (1.5–3.1)	−0.47 (−0.64 to −0.3)
Dominica	2 (1–2)	2.4 (1.56–3.49)	2 (1–3)	2.63 (1.74–3.67)	0.49 (0.21–0.78)
Dominican Republic	146 (93–217)	2.51 (1.66–3.6)	333 (219–488)	3.1 (2.04–4.49)	0.74 (0.56–0.92)
Ecuador	307 (202–447)	3.87 (2.55–5.57)	693 (448–1,000)	3.96 (2.57–5.69)	0.05 (−0.1–0.2)
Egypt	1,774 (1,099–2,656)	3.94 (2.59–5.74)	2,911 (1,925–4,205)	3.36 (2.26–4.77)	−0.39 (−0.45 to −0.32)
El Salvador	378 (245–597)	7.77 (5.16–11.83)	346 (235–481)	5.43 (3.7–7.54)	−0.96 (−1.06 to −0.86)
Equatorial Guinea	7 (4–10)	2.3 (1.5–3.23)	19 (13–29)	2.12 (1.4–3.02)	−0.26 (−0.32 to −0.2)
Eritrea	415 (201–802)	12.35 (6.52–23.48)	205 (122–350)	4.92 (2.9–8.55)	−2.05 (−2.52 to −1.58)
Estonia	179 (118–249)	10.2 (6.68–14.42)	110 (72–157)	6.11 (3.94–8.84)	−2.03 (−2.17 to −1.9)
Eswatini	14 (9–21)	2.67 (1.78–3.74)	27 (17–39)	2.85 (1.86–4.07)	0.08 (−0.08–0.24)
Ethiopia	2,458 (1,223–4,824)	5.52 (3.17–9.73)	2,057 (1,277–3,320)	2.7 (1.79–4)	−1.53 (−2.01 to −1.06)
Fiji	11 (8–17)	1.99 (1.32–2.84)	16 (10–22)	1.9 (1.29–2.73)	−0.25 (−0.34 to −0.15)
Finland	1,583 (1,059–2,185)	24.88 (16.68–34.27)	2,219 (1,464–3,033)	21.06 (14.06–28.63)	−0.67 (−1.07 to −0.26)
France	17,701 (11,893–24,243)	23.7 (16.07–32.74)	23,947 (15,965–33,593)	19.32 (12.95–26.9)	−0.65 (−0.72 to −0.58)
Gabon	19 (12–27)	2.6 (1.74–3.67)	32 (21–45)	2.43 (1.6–3.39)	−0.28 (−0.31 to −0.26)
Gambia	13 (9–19)	2.04 (1.38–2.85)	32 (21–46)	2.16 (1.45–3.1)	0.13 (0.07–0.19)
Georgia	323 (208–463)	5.64 (3.6–8.12)	270 (180–386)	6.3 (4.15–9.08)	0.48 (0.21–0.75)
Germany	19,689 (13,121–27,204)	18.06 (12.03–24.79)	24,987 (16,345–35,063)	15.16 (10.03–21.22)	−0.65 (−0.71 to −0.6)
Ghana	199 (128–292)	2.05 (1.37–2.91)	505 (329–721)	2.19 (1.45–3.11)	0.15 (0.08–0.21)
Greece	2,440 (1,635–3,371)	18.88 (12.77–26.15)	2,209 (1,510–3,020)	12.85 (8.77–17.8)	−1.28 (−1.36 to −1.19)
Greenland	9 (6–12)	23.4 (15.62–32.15)	12 (8–17)	19.48 (12.94–27.24)	−0.69 (−0.76 to −0.61)
Grenada	3 (2–4)	3.17 (2.18–4.71)	3 (2–5)	3.25 (2.16–4.69)	0.03 (−0.06–0.13)
Guam	3 (2–4)	2.42 (1.63–3.5)	4 (3–6)	2.3 (1.5–3.31)	−0.13 (−0.21 to −0.05)
Guatemala	399 (255–629)	6.05 (4.09–9.01)	719 (477–1,038)	5.27 (3.53–7.43)	−0.37 (−0.4 to −0.34)
Guinea	97 (63–139)	2.1 (1.39–2.92)	204 (131–295)	2.26 (1.5–3.21)	0.24 (0.09–0.4)
Guinea-Bissau	18 (11–26)	2.66 (1.73–3.74)	32 (20–46)	2.54 (1.7–3.6)	−0.31 (−0.61 to −0.01)
Guyana	20 (13–29)	3.41 (2.26–4.9)	27 (18–39)	3.85 (2.52–5.5)	0.23 (0.11–0.36)
Haiti	161 (102–239)	3.18 (2.1–4.53)	509 (331–770)	4.75 (3.11–7.23)	1.93 (0.75–3.12)
Honduras	145 (91–213)	3.82 (2.53–5.38)	316 (208–450)	3.72 (2.46–5.23)	−0.31 (−0.73–0.11)
Hungary	1,530 (1,001–2,142)	12.27 (7.99–17.25)	1,152 (759–1,609)	7.69 (4.99–10.94)	−1.88 (−2.02 to −1.74)
Iceland	48 (33–68)	17.51 (11.89–24.49)	72 (48–99)	14.28 (9.53–19.75)	−0.69 (−0.74 to −0.64)
India	27,749 (18,074–40,102)	4.9 (3.3–6.96)	56,954 (37,728–81,877)	4.78 (3.16–6.73)	−0.18 (−0.22 to −0.13)
Indonesia	4,603 (3,027–6,711)	3.34 (2.26–4.8)	6,515 (4,453–9,279)	2.71 (1.86–3.86)	−0.81 (−0.93 to −0.69)
Iran (Islamic Republic of)	2,952 (1,928–4,463)	6.45 (4.43–9.32)	3,570 (2,461–5,054)	4.2 (2.89–5.92)	−1.32 (−1.42 to −1.21)
Iraq	1,278 (798–2,040)	8.32 (5.25–12.95)	2,432 (1,560–3,795)	6.93 (4.44–10.77)	0.12 (−0.33–0.57)
Ireland	599 (401–826)	15.86 (10.66–21.83)	917 (607–1,303)	13.64 (9.11–19.34)	−0.56 (−0.75 to −0.38)
Israel	609 (402–849)	12.82 (8.47–17.84)	1,280 (869–1,749)	11.55 (7.9–15.98)	−0.35 (−0.56 to −0.15)
Italy	17,940 (11,854–24,682)	23.54 (15.73–32.42)	18,691 (12,271–25,926)	16.17 (10.78–22.22)	−1.38 (−1.44 to −1.32)
Jamaica	57 (37–84)	2.62 (1.71–3.79)	83 (55–121)	2.74 (1.8–4.02)	0 (−0.13–0.13)
Japan	21,315 (14,540–29,303)	13.96 (9.54–19.33)	26,446 (17,547–36,386)	9.49 (6.44–13.03)	−1.4 (−1.54 to −1.26)
Jordan	93 (58–137)	3.33 (2.19–4.82)	289 (184–423)	2.68 (1.75–3.87)	−0.68 (−0.73 to −0.64)
Kazakhstan	880 (561–1,302)	5.69 (3.68–8.28)	957 (636–1,379)	5.1 (3.38–7.33)	−0.25 (−0.37 to −0.12)
Kenya	319 (204–462)	2.28 (1.53–3.18)	749 (499–1,065)	2.35 (1.58–3.27)	0.02 (−0.12–0.16)
Kiribati	1 (1–2)	1.86 (1.26–2.62)	2 (1–2)	1.6 (1.07–2.25)	−0.56 (−0.74 to −0.38)
Kuwait	95 (53–165)	6.12 (3.61–10.08)	169 (109–245)	3.6 (2.32–5.13)	−1.18 (−1.57 to −0.79)
Kyrgyzstan	203 (130–298)	5.11 (3.33–7.32)	218 (140–317)	3.47 (2.27–4.95)	−1.4 (−1.47 to −1.32)
Lao People’s Democratic Republic	105 (65–165)	3.19 (2.06–4.78)	148 (97–212)	2.47 (1.64–3.51)	−0.54 (−0.63 to −0.45)
Latvia	352 (231–497)	11.58 (7.53–16.56)	180 (121–251)	6.75 (4.36–9.66)	−2.33 (−2.55 to −2.12)
Lebanon	215 (124–384)	8.02 (4.78–14.08)	235 (154–366)	4 (2.59–6.28)	−2.06 (−2.33 to −1.8)
Lesotho	27 (17–38)	2.28 (1.52–3.16)	47 (30–67)	3.07 (2.02–4.34)	1.07 (0.88–1.25)
Liberia	131 (59–274)	5.41 (2.71–10.65)	99 (65–151)	2.73 (1.84–4.04)	−1.54 (−2.32 to −0.75)
Libya	137 (89–200)	4.23 (2.85–6.13)	334 (229–468)	5.25 (3.61–7.23)	1.6 (1.07–2.13)
Lithuania	419 (277–593)	10.33 (6.84–14.7)	299 (197–421)	7.3 (4.8–10.54)	−1.37 (−1.58 to −1.15)
Luxembourg	110 (74–154)	22.86 (15.23–31.76)	168 (113–233)	17.72 (11.75–24.38)	−0.85 (−0.89 to −0.82)
Madagascar	161 (102–237)	1.86 (1.23–2.66)	324 (207–482)	1.69 (1.12–2.37)	−0.39 (−0.44 to −0.33)
Malawi	126 (80–188)	1.89 (1.23–2.72)	231 (151–342)	1.9 (1.27–2.72)	−0.08 (−0.14 to −0.02)
Malaysia	357 (229–522)	2.73 (1.78–3.95)	834 (543–1,194)	2.81 (1.85–4.03)	0.07 (0.05–0.09)
Maldives	4 (3–6)	2.78 (1.85–4)	13 (8–19)	2.83 (1.89–4.1)	0.13 (−0.19–0.45)
Mali	163 (101–241)	2.6 (1.69–3.68)	437 (288–643)	2.81 (1.9–3.9)	0.02 (−0.52–0.57)
Malta	66 (44–91)	16.58 (11.01–22.83)	108 (72–150)	14.5 (9.69–20.1)	−0.35 (−0.49 to −0.22)
Marshall Islands	1 (0–1)	2.6 (1.75–3.74)	1 (1–2)	2.59 (1.7–3.79)	−0.03 (−0.08–0.02)
Mauritania	37 (24–53)	2.57 (1.75–3.63)	67 (44–96)	2.25 (1.49–3.17)	−0.49 (−0.53 to −0.45)
Mauritius	21 (13–30)	2.13 (1.38–3.08)	33 (22–47)	2.16 (1.43–3.09)	0.29 (0.19–0.39)
Mexico	4,500 (2,875–6,590)	6.8 (4.46–9.7)	5,779 (3,794–8,181)	4.51 (2.97–6.38)	−0.58 (−0.89 to −0.27)
Micronesia (Federated States of)	2 (1–3)	2.72 (1.81–3.84)	2 (2–4)	2.94 (1.95–4.11)	0.26 (0.02–0.5)
Monaco	7 (5–9)	13.18 (8.8–18.4)	9 (6–12)	12.56 (8.39–17.39)	−0.09 (−0.2–0.01)
Mongolia	83 (53–123)	4.75 (3.12–6.86)	167 (108–245)	5.4 (3.51–7.84)	0.52 (0.44–0.6)
Montenegro	52 (33–74)	8.27 (5.33–11.78)	52 (34–74)	7.03 (4.56–10.18)	−0.48 (−0.55 to −0.42)
Morocco	906 (585–1,354)	4.34 (2.87–6.35)	1,370 (895–1,990)	3.86 (2.54–5.57)	−0.48 (−0.55 to −0.41)
Mozambique	400 (233–696)	3.69 (2.27–6.22)	545 (362–793)	2.93 (2.03–4.21)	−0.55 (−0.66 to −0.45)
Myanmar	1,198 (781–1,796)	3.63 (2.41–5.28)	2,164 (1,419–3,160)	4.15 (2.8–5.98)	0.38 (−0.28–1.05)
Namibia	31 (21–44)	2.93 (2.07–4.13)	51 (35–75)	2.66 (1.83–3.77)	−0.47 (−0.59 to −0.34)
Nauru	0 (0–0)	3.04 (2–4.32)	0 (0–0)	3.33 (2.14–4.77)	0.21 (0.13–0.3)
Nepal	483 (305–708)	3.52 (2.33–5.08)	1,115 (743–1,613)	4.24 (2.85–6.03)	0.59 (0.24–0.95)
Netherlands	2,484 (1,669–3,408)	13.56 (9.13–18.69)	4,812 (3,175–6,826)	15.1 (9.98–21.26)	0.71 (0.22–1.21)
New Zealand	900 (609–1,243)	24.57 (16.63–34.01)	1,383 (920–1,893)	19.48 (13.1–26.77)	−0.63 (−0.7 to −0.56)
Nicaragua	193 (117–330)	5.83 (3.67–9.48)	251 (166–373)	4.44 (2.96–6.52)	−0.9 (−1.06 to −0.74)
Niger	126 (79–188)	2.4 (1.62–3.4)	386 (247–573)	2.47 (1.67–3.52)	0.15 (0.07–0.23)
Nigeria	1,446 (945–2,091)	2.25 (1.5–3.17)	3,350 (2,203–4,772)	2.27 (1.55–3.17)	0.02 (−0.05–0.09)
Niue	0 (0–0)	2.5 (1.66–3.6)	0 (0–0)	2.52 (1.67–3.6)	−0.08 (−0.37–0.21)
North Macedonia	119 (77–175)	6.07 (3.94–8.89)	149 (97–216)	6.05 (3.92–8.93)	0.01 (−0.15–0.17)
Northern Mariana Islands	1 (1–2)	3.77 (2.52–5.46)	2 (1–3)	3.74 (2.46–5.35)	−0.08 (−0.14 to −0.03)
Norway	1,286 (843–1,776)	21.02 (13.89–28.66)	1,502 (972–2,106)	16.02 (10.53–22.4)	−0.89 (−0.99 to −0.79)
Oman	97 (61–144)	7.05 (4.62–10.09)	195 (124–288)	5.37 (3.56–7.67)	−1.06 (−1.23 to −0.9)
Pakistan	1,683 (1,101–2,392)	2.05 (1.38–2.88)	3,852 (2,643–5,454)	2.1 (1.45–2.92)	0.27 (−0.05–0.6)
Palau	1 (0–1)	4.32 (2.82–6.18)	1 (1–1)	4.62 (3.06–6.68)	0.2 (0.17–0.23)
Palestine	100 (62–156)	5.87 (3.78–8.74)	229 (144–346)	5.58 (3.58–8.57)	0.21 (−0.54–0.96)
Panama	80 (50–118)	3.85 (2.48–5.53)	140 (92–200)	3.2 (2.1–4.59)	−0.69 (−0.72 to −0.65)
Papua New Guinea	74 (48–108)	2.82 (1.85–3.98)	244 (160–347)	3.52 (2.31–4.92)	0.42 (0.09–0.74)
Paraguay	134 (85–199)	3.98 (2.58–5.73)	271 (176–395)	4.02 (2.65–5.78)	0.03 (−0.05–0.11)
Peru	723 (459–1,112)	3.75 (2.47–5.55)	1,148 (758–1,630)	3.2 (2.12–4.53)	−0.35 (−0.45 to −0.25)
Philippines	1,728 (1,131–2,561)	3.49 (2.38–5.02)	2,587 (1,782–3,631)	2.64 (1.87–3.67)	−0.71 (−0.86 to −0.56)
Poland	3,699 (2,406–5,255)	9.22 (6–13.18)	3,912 (2,649–5,450)	7.29 (4.71–10.44)	−0.86 (−0.96 to −0.77)
Portugal	2,340 (1,581–3,219)	19.58 (13.18–27)	2,323 (1,579–3,222)	11.83 (7.94–16.27)	−1.87 (−1.95 to −1.79)
Puerto Rico	128 (84–183)	3.58 (2.36–5.1)	209 (139–294)	3.99 (2.65–5.69)	0.41 (0.31–0.52)
Qatar	20 (12–30)	5.17 (3.38–7.41)	128 (79–191)	4.61 (3–6.71)	−0.33 (−0.46 to −0.21)
Republic of Korea	8,680 (5,747–12,042)	23.62 (15.98–32.43)	13,097 (8,881–17,905)	16.14 (10.95–22.12)	−1.51 (−1.62 to −1.4)
Republic of Moldova	357 (232–510)	8.06 (5.25–11.44)	232 (155–333)	5.12 (3.38–7.38)	−1.67 (−1.75 to −1.6)
Romania	2,312 (1,502–3,355)	9.33 (6.03–13.61)	1,802 (1,198–2,586)	7.18 (4.72–10.54)	−0.99 (−1.05 to −0.93)
Russian Federation	14,876 (9,809–21,123)	9.18 (6.06–13.06)	13,774 (9,314–19,251)	7.62 (5–10.86)	−0.9 (−1.27 to −0.53)
Rwanda	203 (112–357)	3.45 (2.12–5.61)	418 (240–747)	4.66 (2.65–8.31)	−0.66 (−2.22–0.93)
Saint Kitts and Nevis	1 (1–2)	3.23 (2.1–4.75)	2 (1–3)	3.39 (2.23–4.92)	0.23 (0.12–0.35)
Saint Lucia	3 (2–5)	2.71 (1.77–3.96)	6 (4–8)	2.78 (1.82–4.09)	0.03 (−0.06–0.13)
Saint Vincent and the Grenadines	3 (2–4)	2.69 (1.81–3.88)	4 (3–5)	3.06 (1.99–4.33)	0.34 (0.22–0.45)
Samoa	3 (2–5)	2.61 (1.73–3.77)	5 (3–6)	2.73 (1.86–3.81)	0.45 (−0.07–0.97)
San Marino	4 (3–6)	14.29 (9.45–19.68)	8 (5–11)	13.41 (8.99–18.62)	−0.13 (−0.21 to −0.04)
Sao Tome and Principe	2 (1–3)	2.42 (1.6–3.47)	5 (3–7)	3.25 (2.14–4.7)	0.83 (0.79–0.87)
Saudi Arabia	925 (577–1,374)	8.33 (5.45–11.94)	3,530 (2,192–5,276)	10.18 (6.75–14.64)	0.8 (0.74–0.87)
Senegal	104 (67–153)	1.98 (1.32–2.82)	221 (146–320)	2.07 (1.4–2.94)	0.05 (−0.03–0.12)
Serbia	686 (447–982)	6.87 (4.48–9.92)	740 (492–1,045)	6.3 (4.17–9.13)	−0.46 (−0.73 to −0.18)
Seychelles	2 (1–2)	2.7 (1.76–3.83)	3 (2–4)	2.44 (1.63–3.43)	−0.37 (−0.44 to −0.3)
Sierra Leone	63 (41–91)	1.99 (1.33–2.83)	149 (100–214)	2.5 (1.75–3.59)	−0.89 (−1.65 to −0.13)
Singapore	336 (225–467)	12.12 (8.24–16.76)	743 (497–1,031)	9.97 (6.68–13.89)	−0.74 (−0.91 to −0.58)
Slovakia	596 (389–851)	10.7 (7.02–15.33)	640 (420–919)	8.79 (5.69–12.79)	−0.57 (−0.64 to −0.5)
Slovenia	295 (197–416)	13.28 (8.82–18.93)	356 (237–497)	10.18 (6.69–14.65)	−0.49 (−0.7 to −0.27)
Solomon Islands	8 (5–11)	3.95 (2.6–5.52)	21 (14–30)	4.64 (3.06–6.6)	0.53 (0.44–0.61)
Somalia	218 (115–417)	3.45 (2.07–5.84)	455 (285–698)	3.09 (2–4.54)	0.18 (−0.3–0.66)
South Africa	1,268 (830–1,803)	4.21 (2.85–5.88)	1,515 (1,020–2,109)	2.76 (1.89–3.82)	−1.56 (−1.69 to −1.42)
South Sudan	99 (65–148)	2.27 (1.56–3.22)	204 (132–309)	2.86 (1.92–4.23)	0.8 (0.13–1.46)
Spain	7,473 (5,075–10,207)	15.98 (10.85–21.87)	10,258 (6,923–14,152)	13.14 (8.78–18.26)	−0.72 (−0.84 to −0.6)
Sri Lanka	932 (536–1,629)	5.8 (3.55–9.62)	1,233 (847–1,714)	5.11 (3.51–7.11)	−0.19 (−0.76–0.38)
Sudan	762 (468–1,196)	4.52 (2.93–6.74)	1,225 (806–1,798)	3.65 (2.49–5.2)	−0.46 (−0.71 to −0.22)
Suriname	9 (6–14)	2.71 (1.83–3.92)	17 (11–24)	2.81 (1.89–3.98)	0.16 (0.1–0.23)
Sweden	2,292 (1,525–3,205)	17.73 (11.89–24.56)	2,731 (1,771–3,799)	14.16 (9.29–19.42)	−0.7 (−0.82 to −0.57)
Switzerland	2,602 (1,724–3,612)	27.4 (18.28–37.96)	3,071 (2,008–4,241)	18.9 (12.5–26.36)	−1.36 (−1.41 to −1.3)
Syrian Arab Republic	312 (203–470)	3.16 (2.13–4.66)	885 (536–1,478)	6.84 (4.05–11.64)	4.81 (3.53–6.12)
Taiwan (Province of China)	773 (501–1,111)	4.3 (2.82–6.15)	865 (575–1,218)	2.43 (1.61–3.46)	−2.27 (−2.45 to −2.08)
Tajikistan	190 (118–286)	4.23 (2.77–6.12)	311 (203–464)	3.53 (2.35–5.19)	−1.89 (−2.5 to −1.27)
Thailand	1,889 (1,234–2,802)	3.89 (2.58–5.73)	3,074 (2,054–4,307)	3.5 (2.35–4.96)	−0.56 (−0.66 to −0.46)
Timor-Leste	41 (23–71)	5.74 (3.39–9.38)	45 (28–72)	4.38 (2.71–7.21)	−1.58 (−2.22 to −0.94)
Togo	52 (34–77)	2.26 (1.49–3.23)	128 (82–183)	2.28 (1.5–3.23)	−0.06 (−0.15–0.03)
Tokelau	0 (0–0)	2.27 (1.5–3.25)	0 (0–0)	2.29 (1.52–3.28)	−0.01 (−0.05–0.04)
Tonga	2 (1–2)	2.09 (1.38–3.03)	2 (1–2)	1.82 (1.2–2.58)	−0.39 (−0.54 to −0.24)
Trinidad and Tobago	30 (20–45)	2.87 (1.89–4.15)	45 (31–64)	2.91 (1.96–4.14)	0.34 (0.21–0.47)
Tunisia	280 (181–414)	3.98 (2.61–5.77)	453 (305–657)	3.63 (2.43–5.27)	−0.3 (−0.35 to −0.24)
Turkey	1,527 (995–2,210)	3.09 (2.06–4.39)	2,817 (1,847–4,039)	3.25 (2.13–4.66)	0.23 (0.11–0.35)
Turkmenistan	123 (78–183)	3.77 (2.47–5.47)	152 (98–220)	3.06 (2–4.41)	−0.82 (−0.95 to −0.69)
Tuvalu	0 (0–0)	2.64 (1.74–3.77)	0 (0–0)	2.71 (1.79–3.87)	−0.36 (−0.53 to −0.18)
Uganda	397 (246–636)	3.07 (2.01–4.65)	651 (433–936)	2.57 (1.76–3.67)	−0.67 (−0.81 to −0.53)
Ukraine	5,534 (3,636–7,955)	9.39 (6.13–13.69)	4,249 (2,846–6,044)	7.79 (5.03–11.16)	−1.11 (−1.31 to −0.91)
United Arab Emirates	76 (47–113)	5.22 (3.45–7.5)	512 (325–748)	4.91 (3.18–7.02)	−0.24 (−0.27 to −0.2)
United Kingdom	10,646 (7,164–14,656)	14.03 (9.48–19.4)	14,287 (9,503–20,026)	12.87 (8.63–17.78)	−0.22 (−0.35 to −0.09)
United Republic of Tanzania	370 (237–545)	2.06 (1.38–2.91)	812 (523–1,165)	2.06 (1.35–2.86)	−0.04 (−0.07–0)
United States of America	47,887 (32,422–66,382)	16.15 (11.01–22.33)	83,891 (55,788–116,186)	15.92 (10.63–21.96)	−0.08 (−0.17–0.01)
United States Virgin Islands	3 (2–4)	3.19 (2.09–4.6)	4 (3–6)	3.07 (2.05–4.42)	−0.33 (−0.48 to −0.18)
Uruguay	467 (322–649)	13.4 (9.23–18.83)	603 (406–832)	12.92 (8.55–17.89)	−0.18 (−0.22 to −0.15)
Uzbekistan	729 (467–1,074)	4.02 (2.63–5.76)	1,118 (723–1,624)	3.47 (2.27–4.98)	−0.54 (−0.67 to −0.41)
Vanuatu	2 (2–3)	2.27 (1.52–3.14)	5 (3–8)	2.3 (1.52–3.28)	−0.05 (−0.23–0.14)
Venezuela (Bolivarian Republic of)	744 (462–1,103)	4.74 (3.08–6.8)	1,187 (801–1,672)	4.29 (2.87–6.08)	−0.46 (−0.71 to −0.21)
Viet Nam	1,639 (1,067–2,384)	3.29 (2.22–4.72)	3,893 (2,577–5,586)	4.12 (2.73–5.91)	0.88 (0.77–0.98)
Yemen	402 (259–594)	4.18 (2.77–5.97)	1,787 (1,046–2,997)	6.04 (3.77–9.53)	1.31 (0.84–1.78)
Zambia	111 (71–162)	2.16 (1.45–3.05)	271 (174–391)	2.32 (1.56–3.28)	0.19 (0.13–0.25)
Zimbabwe	156 (101–224)	2.4 (1.59–3.36)	233 (151–334)	2.22 (1.46–3.12)	−0.36 (−0.45 to −0.26)

In 2021, the number of incidence, prevalence, and YLDs cases were 1.33, 1.01, and 1.04 times higher in males than females. And the corresponding ASRs were 1.40, 1.17, and 1.19 times, respectively (Supplementary Figure S1, Tables 1–3).

Incidence, prevalence, and YLDs across age groups separately in 2021 were available in Supplementary Figure S2. The ASIR, ASPR, and age-standardized YLDs rate was consistently increasing with age. However, the number of incidence, prevalence, and YLDs cases first increased with age and then decreased after reaching the peak (Supplementary Figure S2, Tables 1–3).

At the SDI region level, the high SDI region had the most number of incidence cases at 2,141,941 (95% UI: 1,580,376–2,884,480), prevalence cases at 2,469,882 (95% UI: 2,144,431–2,817,433), and YLDs cases at 245,574 (95% UI: 163,359–339,966). And the highest ASRs also occurred in the high SDI region, which was 157.17 (95% UI: 119.04–207.76) for ASIR, 131.65 (95% UI: 114.84–149.24) for ASPR, and 13.36 (95% UI: 8.95–18.51) for the age-standardized YLDs rate, respectively (Supplementary Figure S3, Tables 1–3).

Across the 54 GBD regions, Asia ranked the top one in fracture of vertebral column-related incidence, followed by Advanced Health System and Basic Health System. Advanced Health System ranked the top one for number of prevalence and YLDs cases, followed by World Bank High Income and Asia. However, Oceania rank the bottom one for number of cases. For the corresponding ASRs, the top GBD region was Australasia, the bottom was Commonwealth Low Income, followed by Western Africa and Western Sub-Saharan Africa (Supplementary Figure S4, Tables 1–3).

**Figure 1:**
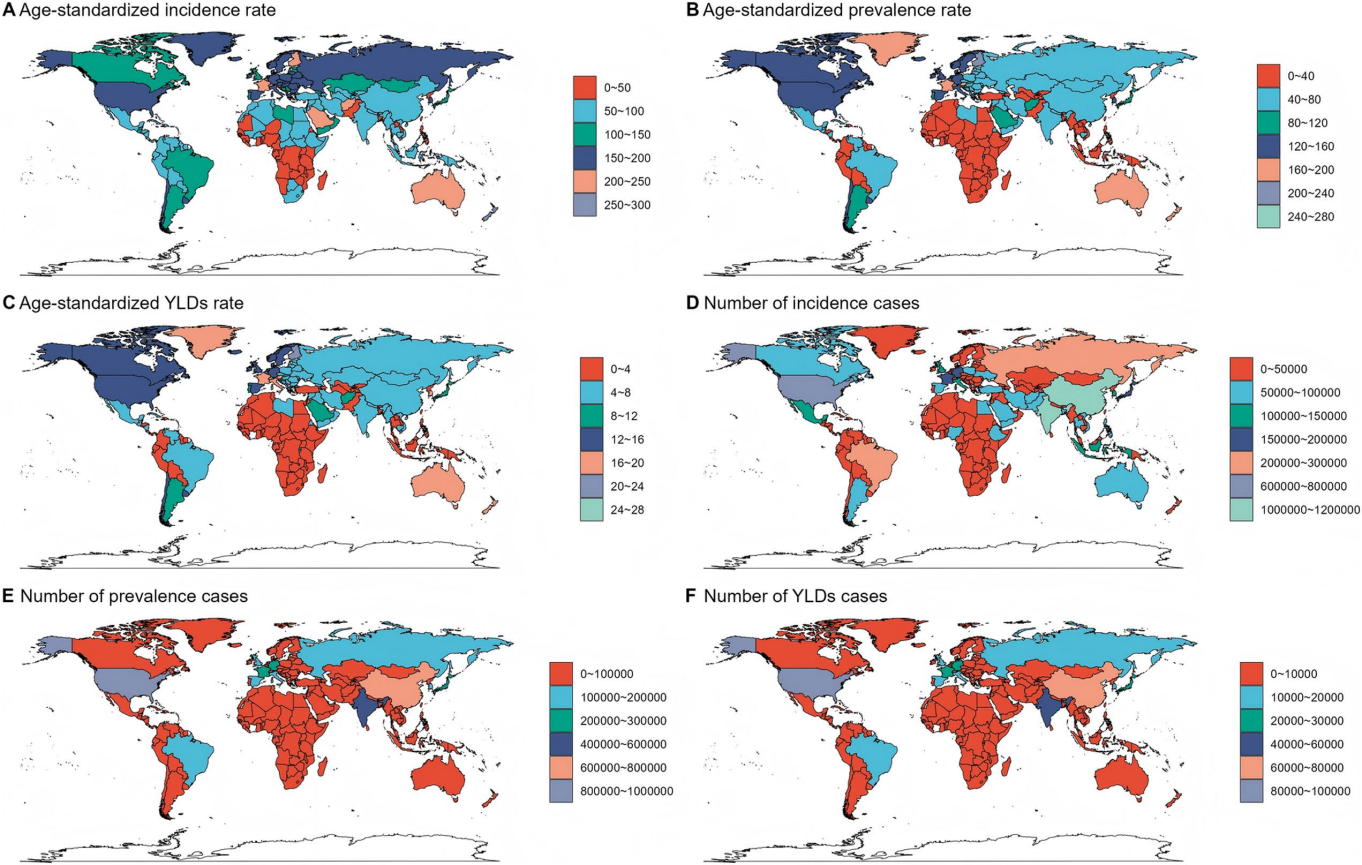
Numbers and age-standardized rates of fracture of vertebral column-related incidence, prevalence, and YLDs across countries and territories in 2021.

The disease burden of fracture of vertebral column varied considerably across the world, with the top ASRs observed in Andorra in 2021. The lowest ASRs for incidence, prevalence, and YLDs were in Kiribati, followed by Madagascar. As for the absolute number, the highest number of incidence cases was observed in China, followed by India and United States of America. And the highest number of prevalence and YLDs cases was also observed in United States of America in 2021, followed by China and India. The lowest number of cases were all observed in Tokelau, followed by Niue (Figure 1, Tables 1–3).

### Temporal trend for fracture of vertebral column-related disease burden from 1990 to 2021

**Figure 2:**
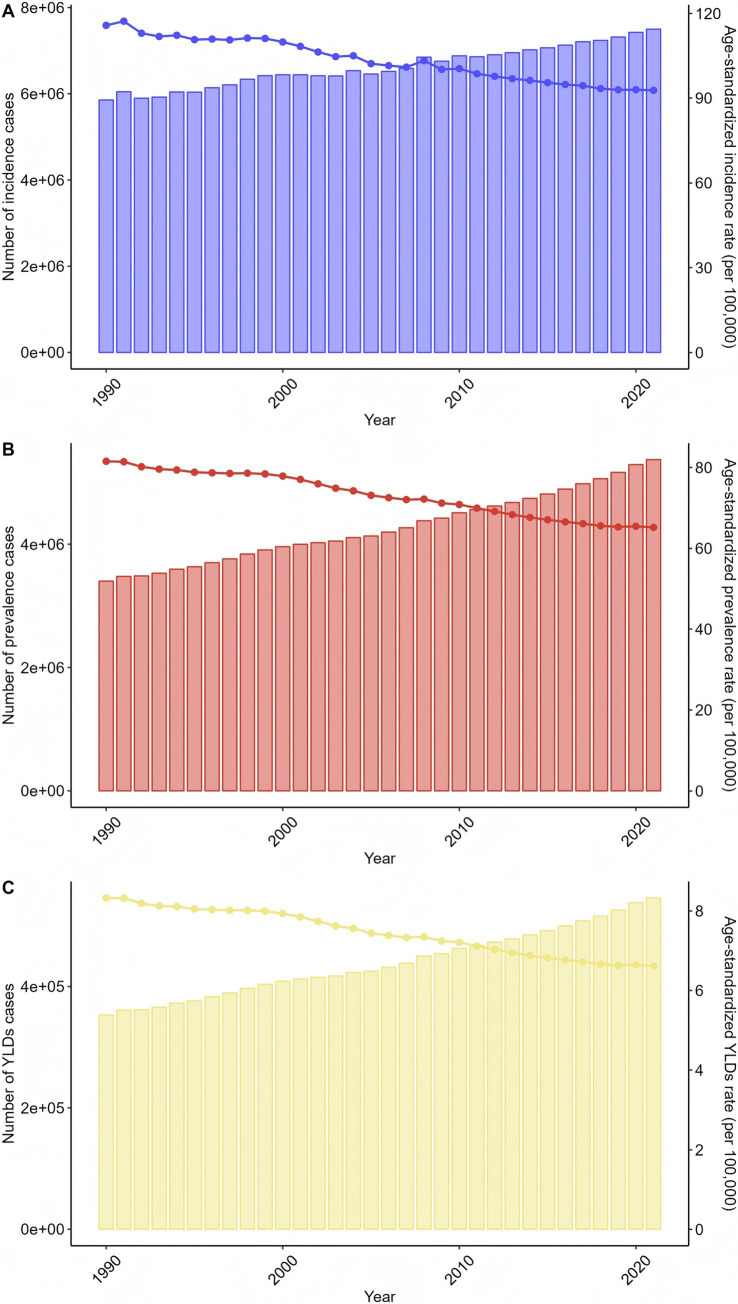
Trends in the numbers and age-standardized rates of fracture of vertebral column-related incidence, prevalence, and YLDs globally from 1990 to 2021.

The number of incidence cases of fracture of vertebral column increased from 5,856,226 (95% UI: 4,615,149–7,402,697) in 1990 to 7,497,446 (95% UI: 5,834,963–9,737,255) in 2021 globally. However, the corresponding ASIR changed in the opposite direction, with the decrease from to 115.75 (95% UI: 91.28–145.92) to 92.75 (95% UI: 72.12–119.99). Prevalence and YLDs estimate followed the same pattern, with the number of prevalence cases increased from 3,400,460 (95% UI: 2,958,279–3,925,843) to 5,371,438 (95% UI: 4,703,837–6,196,132), the number of YLDs cases increased from 352,960 (95% UI: 235,711–491,606) to 545,923 (95% UI: 366,571–757,099), the ASPR decreased from 81.55 (95% UI: 71.55–93.04) to 65.19 (95% UI: 56.89–75.28), and the age-standardized YLDs cases decreased from 8.33 (95% UI: 5.61–11.49) to 6.62 (95% UI: 4.43–9.2) per 100,000 population (Figure 2, Tables 1–3).

The trends in males and females alone were consistent with the whole population (Supplementary Figure S5, Tables 1–3). Moreover, the trends were also consistent for most age groups except for older adults (Supplementary Figure S6, Tables 1–3). At the SDI region level, all SDI regions showed the same trend as the whole population (Supplementary Figure S7, Tables 1–3).

**Figure 3:**
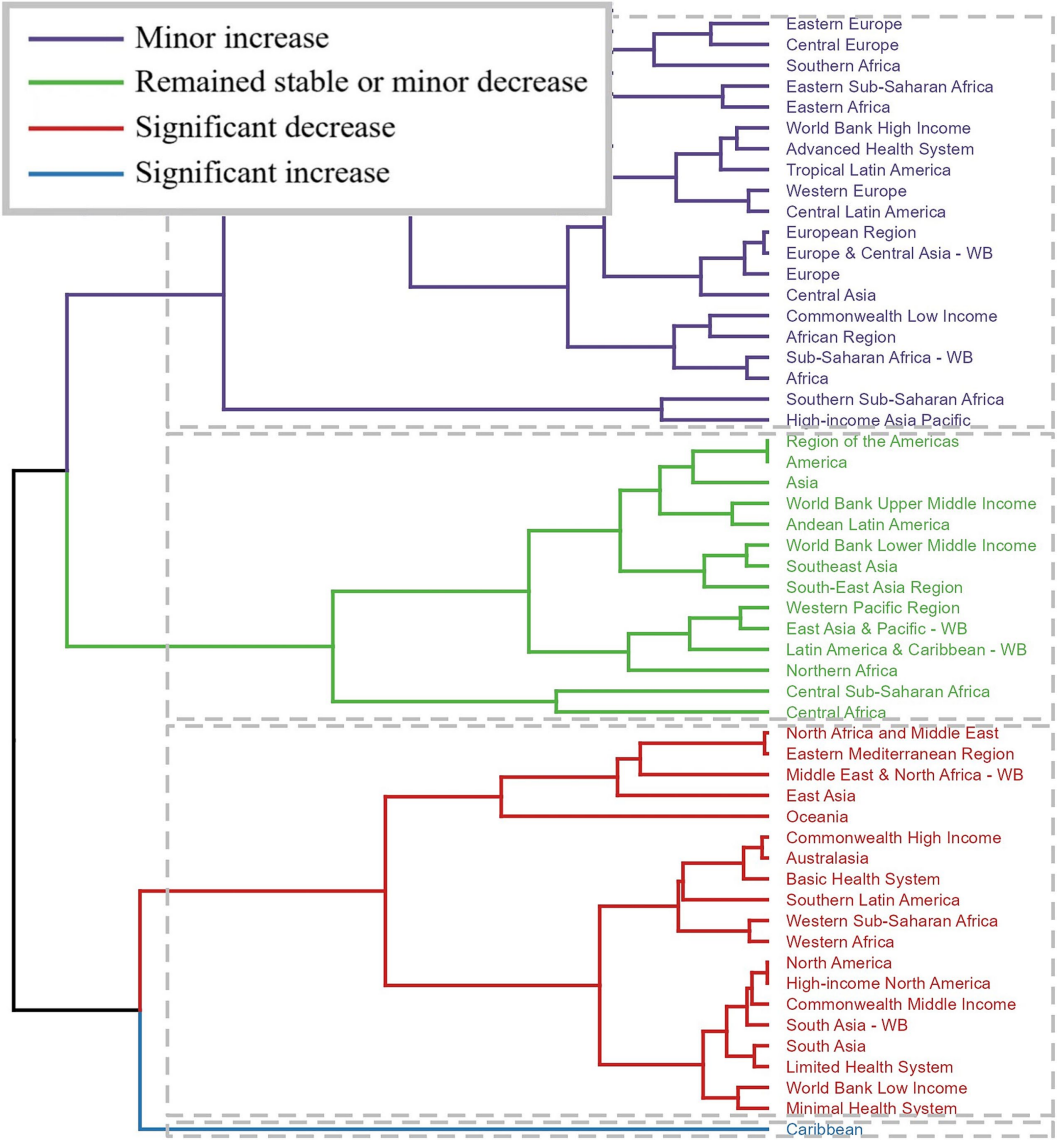
Results of cluster analysis based on the EAPC values of the fracture of vertebral column-related age-standardized rates for incidence, prevalence, and YLDs from 1990 to 2021.

Across GBD regions, the trend of the fracture of vertebral column-related disease burden varied. The results of cluster analysis were shown in Figure 3. The significant incidence, prevalence, and YLDs rate increase occurred in Caribbean, while the significant decrease was in North Africa and Middle East, Eastern Mediterranean Region, Middle East & North Africa-WB, East Asia, Oceania, Commonwealth High Income, Australasia, Basic Health System, Southern Latin America, Western Sub-Saharan Africa, Western Africa, North America, High-income North America, Commonwealth Middle Income, South Asia-WB, South Asia, Limited Health System, World Bank Low Income, and Minimal Health System (Figure 3).

**Figure 4:**
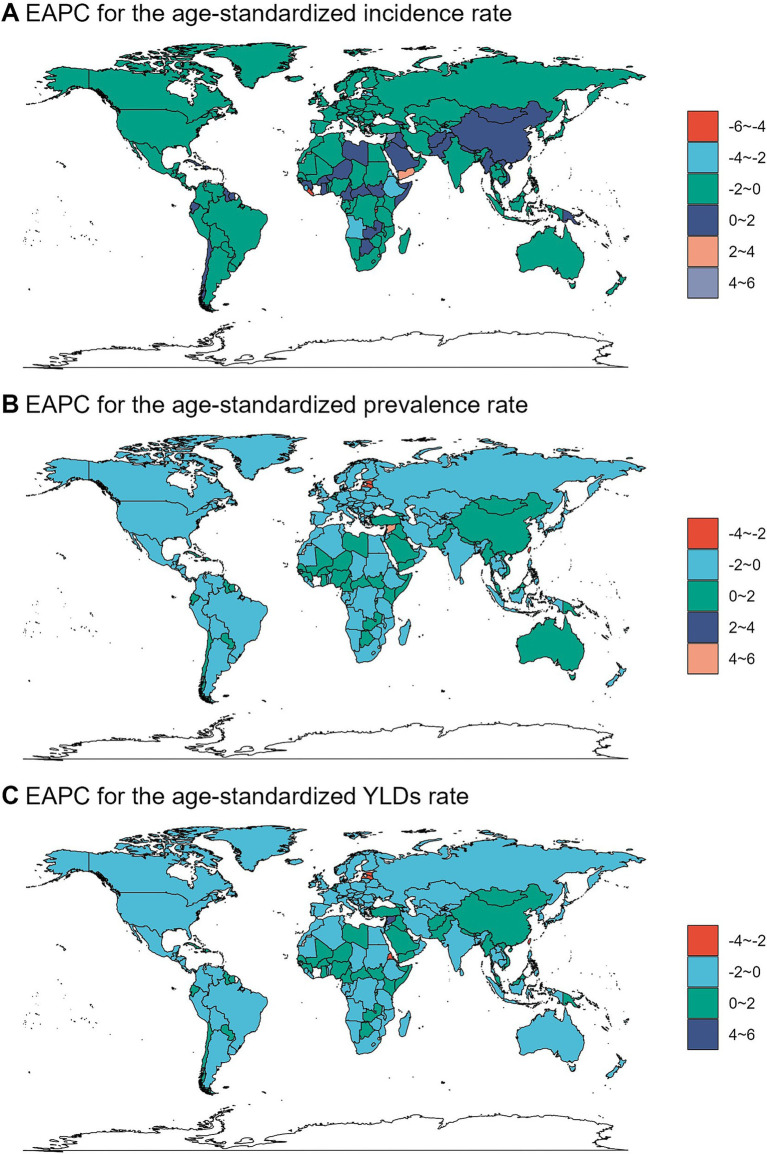
The EAPC of fracture of vertebral column-related ASRs from 1990 to 2021.

Across countries and territories, the changing trend was also different. The most pronounced increase in ASIR [EAPC = 5.24, 95% confidence interval (CI): 3.34–7.19], ASPR (EAPC = 4.79, 95% CI: 3.53–6.06), and the age-standardized YLDs cases (EAPC = 4.81, 95% CI: 3.53–6.12) from 1990 to 2021 was observed in Syrian Arab Republic. The most pronounced decrease in ASIR (EAPC = −4.61, 95% CI: −6.07 to −3.12) was Timor-Leste. And the most pronounced decrease in ASPR (EAPC = −2.35, 95% CI: −2.57 to −2.13) and the age-standardized YLDs cases (EAPC = −2.33, 95% CI: −2.55 to −2.12) was Latvia (Figure 4, Tables 1–3).

### The predicted results from 2022 to 2046

**Figure 5:**
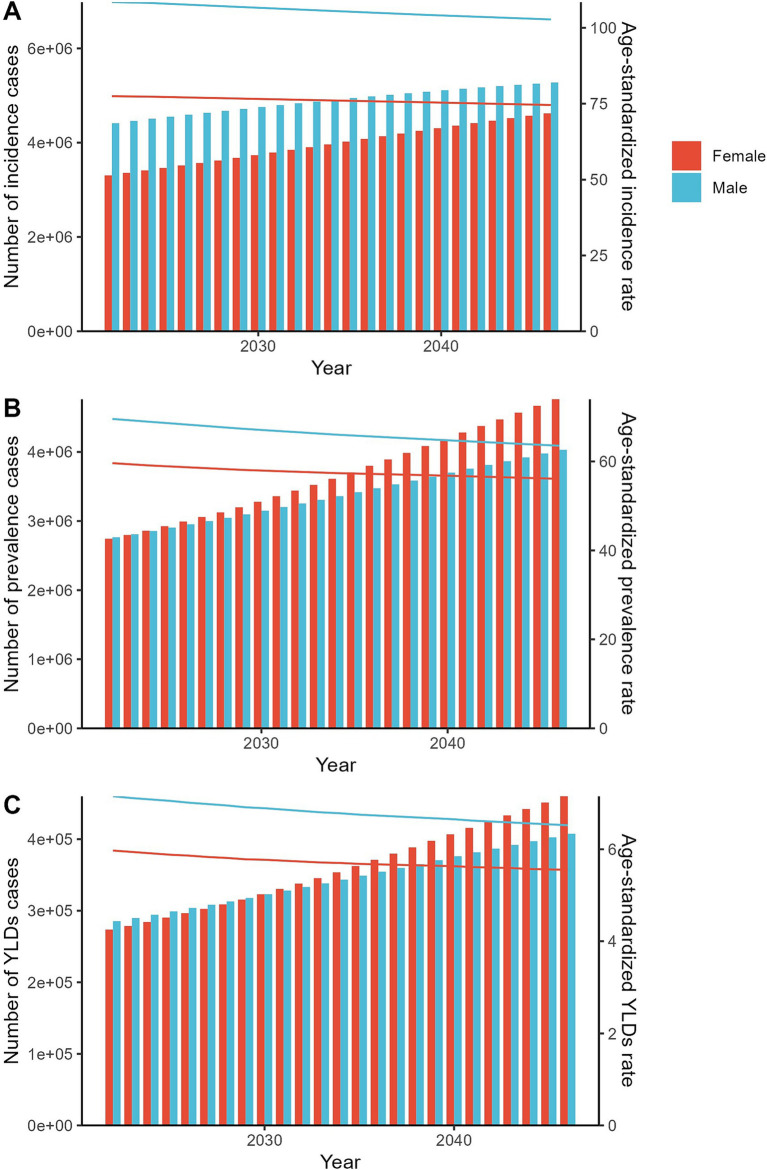
The predicted results in the fracture of vertebral column-related numbers and age-standardized rates of incidence, prevalence, and YLDs by sex globally from 2022 to 2046 of the APC model.

The predicted results of the APC model showed that the number of incidence, prevalence, and YLDs cases for both genders would increase from 2022 to 2046. The number of incidence cases for males was 4,412,932 in 2021, and was 276,314 in 2046. The number of prevalence cases was 2,766,006 in 2022, and was 4,031,334 in 2046. And the number of YLDs was 285,497 in 2022, and was 407,631 in 2046. For females, the number of incidence cases increased from 3,306,504 in 2022 to 4,620,813 in 2046. The number of prevalence cases increased from 2,743,602 to 4,764,050 during this period. And the number of YLDs cases increased from 273,619 to 460,041. Although the predicted results show the number of cases will continue to increase, but the ASRs showed the decreasing trend over the next 25 years for both genders. In 2021, the ASIR for males was 108.50 per 100,000 population, and was 102.79 in 2046. The ASPR for males was 69.60 in 2022, and was 63.57 in 2046. During the same period, the age-standardized YLDs cases decreased from 7.15 to 6.52. For females, the ASIR decreased from 77.48 to 74.59, the ASPR decreased from 59.65 to 56.13, and the age-standardized YLDs cases decreased from 5.97 to 5.55 (Figure 5, Table 4).

**Table 4:** The predicted results in the fracture of vertebral column-related numbers and age-standardized rates of incidence, prevalence, and YLDs by sex globally from 2022 to 2046 of the APC model.

Year	Sex	Age-standardized incidence rate	Number of incidence cases	Age-standardized prevalence rate	Number of prevalence cases	Age-standardized YLDs rate	Number of YLDs cases
2022	Female	77.48	3,306,504	59.65	2,743,602	5.97	273618.7
2023	Female	77.41	3,357,859	59.39	2,798,456	5.94	278640.6
2024	Female	77.35	3,411,460	59.13	2,859,325	5.91	284194.7
2025	Female	77.22	3,463,814	58.93	2,925,935	5.88	290357.5
2026	Female	77.09	3,516,449	58.73	2,992,627	5.86	296519.5
2027	Female	76.96	3,568,723	58.53	3,057,811	5.83	302534.5
2028	Female	76.84	3,622,123	58.33	3,125,704	5.81	308,784
2029	Female	76.71	3,677,743	58.13	3,199,247	5.78	315535.2
2030	Female	76.58	3,734,455	57.99	3,279,061	5.77	322,960
2031	Female	76.46	3,791,352	57.84	3,359,928	5.75	330467.6
2032	Female	76.34	3,847,637	57.7	3,440,148	5.73	337900.6
2033	Female	76.21	3,904,475	57.56	3,523,020	5.71	345557.9
2034	Female	76.09	3,963,031	57.41	3,611,201	5.7	353682.5
2035	Female	75.97	4,021,753	57.32	3,704,869	5.68	362416.8
2036	Female	75.85	4,079,965	57.22	3,799,058	5.67	371182.7
2037	Female	75.73	4,136,757	57.13	3,891,798	5.66	379798.6
2038	Female	75.62	4,193,345	57.03	3,986,322	5.65	388558.2
2039	Female	75.5	4,250,882	56.94	4,085,267	5.64	397702.6
2040	Female	75.37	4,306,755	56.82	4,183,824	5.63	406802.1
2041	Female	75.24	4,361,462	56.71	4,281,482	5.61	415803.2
2042	Female	75.11	4,414,167	56.59	4,376,146	5.6	424515.9
2043	Female	74.98	4,466,162	56.48	4,471,074	5.59	433234.6
2044	Female	74.85	4,518,507	56.36	4,568,939	5.57	442202.8
2045	Female	74.72	4,570,329	56.25	4,667,302	5.56	451201.8
2046	Female	74.59	4,620,813	56.13	4,764,050	5.55	460040.7
2022	Male	108.5	4,412,933	69.6	2,766,006	7.15	285496.7
2023	Male	108.37	4,460,392	69.28	2,809,484	7.11	289786.2
2024	Male	108.24	4,508,567	68.96	2,856,387	7.08	294386.7
2025	Male	107.97	4,551,324	68.65	2,905,220	7.05	299166.6
2026	Male	107.7	4,593,352	68.33	2,953,328	7.01	303872.9
2027	Male	107.44	4,634,248	68.02	2,999,549	6.98	308396.1
2028	Male	107.17	4,674,883	67.7	3,046,705	6.95	312997.2
2029	Male	106.9	4,715,906	67.39	3,096,754	6.91	317858.4
2030	Male	106.64	4,756,650	67.12	3,150,260	6.89	323064.4
2031	Male	106.38	4,796,482	66.86	3,203,281	6.86	328215.5
2032	Male	106.12	4,835,175	66.6	3,254,686	6.83	333206.3
2033	Male	105.86	4,872,640	66.33	3,306,551	6.8	338225.5
2034	Male	105.6	4,910,143	66.07	3,360,842	6.78	343459.7
2035	Male	105.35	4,946,890	65.85	3,418,537	6.75	349035.5
2036	Male	105.09	4,982,338	65.64	3,475,485	6.73	354530.3
2037	Male	104.84	5,016,101	65.42	3,530,514	6.71	359834.9
2038	Male	104.59	5,048,593	65.21	3,585,726	6.69	365142.7
2039	Male	104.33	5,080,603	65	3,642,906	6.67	370621.4
2040	Male	104.11	5,112,930	64.79	3,700,974	6.65	376,185
2041	Male	103.89	5,143,667	64.59	3,757,853	6.62	381,626
2042	Male	103.67	5,172,427	64.38	3,812,380	6.6	386836.6
2043	Male	103.45	5,199,974	64.18	3,866,518	6.58	391997.7
2044	Male	103.23	5,226,746	63.97	3,921,954	6.56	397266.7
2045	Male	103.01	5,252,317	63.77	3,977,299	6.54	402515.3
2046	Male	102.79	5,276,315	63.57	4,031,335	6.52	407630.7

## Discussion

As far as we know, this was the latest study to comprehensively assess and quantify fracture of vertebral column-related disease burden globally and then predict the future tendency of the disease burden. Globally, fracture of vertebral column caused a severe disease burden in 2021, and significant differences in the disease burden existed between sexes and across ages, SDI regions, GBD regions, and countries. From 1990 to 2021, there was an decreasing trend for the ASRs globally. However, the number of cases were still very severe which showed an increasing trend. Furthermore, our predicted results showed that the number of cases would still increase in the next 25 years.

The incidence and prevalence of vertebral column fractures reported in this study align with a growing body of literature highlighting the significant burden of spinal fractures globally. Our findings indicate a vertebral column fracture incidence of 92.75 per 100,000 population, which is comparable to previous estimates by Johnell and Kanis (17) and Zheng et al. (3), who reported similar incidence rates. The prevalence of 65.19 per 100,000 echoes the trends observed in recent systematic reviews (5, 19). Notably, the number of YLDs attributed to vertebral column fractures, at 545,923, underscores the considerable impact on health outcomes, corroborating the findings of previous study which emphasized the substantial morbidity associated with these fractures (2). The ASR of YLDs of 6.62 per 100,000 further emphasizes the need for preventive measures and improved management strategies. These results emphasize the urgent need for targeted interventions to mitigate the rising incidence and prevalence of vertebral column fractures.

The global incidence of vertebral column fractures has increased from 5,856,226 in 1990 to 7,497,446 in 2021, despite a decrease in the ASIR from 115.75 to 92.75 per 100,000 population over the same period. This trend is mirrored by the prevalence and YLDs estimates, with an increase in the number of cases but a decline in the ASPR and age-standardized YLDs rate per 100,000 population. Our findings contrast with previous studies (3, 17) that reported stable or declining incidence rates of vertebral fractures, possibly due to variations in data sources, study populations, or improvements in fracture prevention and management strategies in recent years. The observed decrease in ASPR and age-standardized YLDs may reflect advancements in healthcare, including early detection, better treatment options, and increased awareness of fracture risk factors (20, 21). However, the overall increase in incidence and prevalence cases highlights the continued need for effective interventions to reduce the burden of vertebral column fractures globally.

Our study reveals significant gender disparities in the incidence, prevalence, and YLDs due to a specific health condition in 2021, with males experiencing 1.33, 1.01, and 1.04 times higher case counts, respectively, compared to females. Correspondingly, the ASRs for males were 1.40, 1.17, and 1.19 times those of females. These findings align with previous research (22, 23) indicating higher vulnerability to this condition among males, potentially attributed to biological, behavioral, and environmental factors. Notably, the observed trends in both males and females followed a similar pattern to the overall population, suggesting that gender-specific risk factors may be influencing the disease burden similarly across different demographics. However, the magnitude of gender disparities in ASRs underscores the need for tailored prevention and management strategies to address the unique needs of males and females, ultimately aiming to reduce the overall disease burden.

Our findings in 2021 indicate a consistent increase in ASIR, ASPR, and age-standardized YLDs rate with advancing age, suggesting an age-related vulnerability to the health condition studied. However, the absolute number of incidence, prevalence, and YLDs cases followed a biphasic pattern, peaking in middle age before declining in older adults, which contrasts with some previous studies reporting a monotonic increase with age (24). This discrepancy might be attributed to differential survival rates among age groups or varying severity of the condition. The trends observed were generally consistent across most age groups, except for older adults, where deviations could be influenced by comorbidities and competing risks of mortality. Our results emphasize the importance of age-specific interventions tailored to address the peak incidence and prevalence periods while considering the decline in cases among the oldest adults, aligning with recommendations from recent literature (25) that advocate for life-course approaches to chronic disease management.

Our results demonstrate that the high SDI region reported the highest number of incidence, prevalence, and YLDs cases for the health condition studied, with 2,141,941 incidence cases, 2,469,882 prevalence cases, and 245,574 YLDs cases, respectively. Consistent with these findings, the highest ASIR, ASPR, and age-standardized YLDs rate were also observed in the high SDI region. These values were 157.17 for ASIR, 131.65 for ASPR, and 13.36 for the age-standardized YLDs rate, respectively. This trend aligns with previous studies (24, 26) that have reported a positive correlation between SDI and the burden of non-communicable diseases. However, it is noteworthy that despite the varying SDI levels, all regions exhibited similar trends to the overall population, indicating a ubiquitous nature of the risk factors associated with this health condition. These findings underscore the need for comprehensive and equitable healthcare strategies across different SDI regions to address the rising burden of this health condition.

In our study, the geographical distribution of vertebral column fracture-related incidence and burden varied significantly across the 54 GBD regions. Asia emerged as the leading region in terms of incidence, followed by regions with Advanced and Basic Health Systems, aligning with previous studies highlighting the high prevalence of spinal injuries in developing countries due to road traffic accidents and occupational hazards (2, 15). However, in contrast to incidence, the Advanced Health System region topped the charts for prevalence and YLDs, followed by World Bank High Income and Asia, suggesting better diagnostic capabilities and longer survival with disability in developed healthcare systems (27, 28). Notably, Oceania reported the lowest number of cases, potentially due to its smaller population and better safety standards. The ASRs revealed Australasia at the peak and Commonwealth Low Income regions at the bottom, indicating a socio-economic gradient in disease burden, consistent with findings from global health disparities research (29). The cluster analysis further elucidated contrasting trends, with Caribbean regions experiencing significant increases in incidence, prevalence, and YLDs, whereas multiple regions, including North Africa, Middle East, and high-income areas like North America, exhibited decreases. These trends might be attributed to improvements in trauma care and public health interventions in some regions, while others still grapple with rising risk factors such as aging populations and osteoporosis (11, 30). Overall, our findings underscore the need for tailored intervention strategies considering regional variations in vertebral column fracture burden.

Our findings reveal substantial variations in the disease burden of fracture of vertebral column globally, with Andorra exhibiting the highest ASRs in 2021, contrasting with the lowest ASRs observed in Kiribati and Madagascar. The absolute numbers indicate China, India, and the USA as the countries with the highest incidence, prevalence, and YLDs cases, while Tokelau and Niue reported the lowest. These observations align partially with prior studies highlighting regional disparities in fracture incidence and outcomes (2, 24). Notably, the Syrian Arab Republic showed the most significant increase in ASIR, ASPR, and YLDs cases from 1990 to 2021, potentially linked to the ongoing conflict and subsequent healthcare disruptions (31). Conversely, Timor-Leste and Latvia experienced notable decreases, suggesting improvements in healthcare infrastructure and injury prevention measures (32). The diverse trends across countries underscore the need for tailored interventions to address the burden of vertebral column fractures, considering socio-economic, political, and healthcare factors.

The APC model’s predictions indicate a rise in incidence, prevalence, and YLDs cases for both genders from 2022 to 2046, aligning with previous forecasts in global health literature (33). However, our study uniquely reveals a concurrent decline in ASRs for these outcomes over the next 25 years. This finding contrasts with studies that projected sustained increases in ASRs for similar conditions (2, 34), suggesting potential improvements in disease prevention, early detection, or management strategies. The observed decrease in ASRs for both males and females, despite the overall increase in case numbers, implies a favorable shift in population health dynamics. These trends highlight the need for continued investment in healthcare infrastructure and interventions to maintain and potentially accelerate the decline in ASRs, while also addressing the growing absolute number of cases. Continuous monitoring and policy adaptations are essential to mitigate the increasing disease burden.

This study is subject to several limitations. The GBD 2021 data rely extensively on statistical modeling, particularly in regions with sparse original data (such as sub-Saharan Africa and parts of Southeast Asia), where input sources may be limited to surveys, hospital registries, or indirect estimates (35). While the GBD consortium employs rigorous Bayesian meta-regression tools (DisMod-MR) to minimize bias and cross-validate estimates, residual uncertainty persists in settings with incomplete vital registration systems (36). Importantly, excluding these modeled data would disproportionately erase the health burden representation of populations in low-resource settings, where fragility fractures are often underdiagnosed and underreported. Retaining these estimates, despite their limitations, allows for a more equitable global perspective, albeit with the caveat that trends in such regions may reflect modeled approximations rather than empirical observations. Furthermore, sensitivity analyses conducted by the GBD collaborators suggest that modeled estimates for spinal fractures in data-scarce regions exhibit wider UIs compared to high-quality data regions. This implies that while absolute values (incidence rates) in these areas should be interpreted cautiously, relative trends (age-standardized rate changes over time) remain robust due to internal consistency in modeling assumptions. To mitigate these limitations, we emphasize that our conclusions prioritize identifying geographic and socioeconomic disparities rather than absolute burden quantification. Future studies incorporating primary data from emerging national registries (the WHO Fracture Risk Audit in LMICs) are urgently needed to validate these models.

## Conclusion

In conclusion, this systematic analysis of the global, regional, and national burden of fracture of vertebral column, with projections to 2046, represents a significant step forward in addressing the challenges posed by this disease. By providing comprehensive and timely data, our study aims to inform evidence-based policies and interventions aimed at reducing the incidence, prevalence, and YLDs lost due to fracture of vertebral column worldwide.

## Data Availability

The original contributions presented in the study are included in the article/supplementary material, further inquiries can be directed to the corresponding author.
