# Supplementary material for: Global, regional, and national burden of fracture of vertebral column, 1990–2021: analysis of data from the global burden of disease study 2021
Source: Front Public Health. 2025 Apr 30;13:1573888. doi: 10.3389/fpubh.2025.1573888 (PMC12075374; doi:10.3389/fpubh.2025.1573888)

**Supplementary Information and Data**

**Global, regional, and national burden of fracture of vertebral column, 1990-2021: analysis of data from the global burden of disease study 2021**

**Supplementary Figures**

**Figure S1.** Numbers and age-standardized rates of fracture of vertebral column related incidence, prevalence, and YLDs for both sex in 2021.

**Figure S2.** Numbers and age-standardized rates of fracture of vertebral column related incidence, prevalence, and YLDs for different age groups in 2021.

**Figure S3.** Numbers and age-standardized rates of fracture of vertebral column related incidence, prevalence, and YLDs for different SDI region in 2021.

**Figure S4.** Numbers and age-standardized rates of fracture of vertebral column related incidence, prevalence, and YLDs for different GBD region in 2021.

**Figure S5.** Trends in the numbers and age-standardized rates of fracture of vertebral column-related incidence, prevalence, and YLDs globally by sex from 1990 to 2021.

**Figure S6.** Trends in the numbers and age-standardized rates of fracture of vertebral column-related incidence, prevalence, and YLDs globally by age groups from 1990 to 2021.

**Figure S7.** Trends in the numbers and age-standardized rates of fracture of vertebral column-related incidence, prevalence, and YLDs globally by SDI region from 1990 to 2021.

**Figure S1.** Numbers and age-standardized rates of fracture of vertebral column related incidence, prevalence, and YLDs for both sex in 2021.


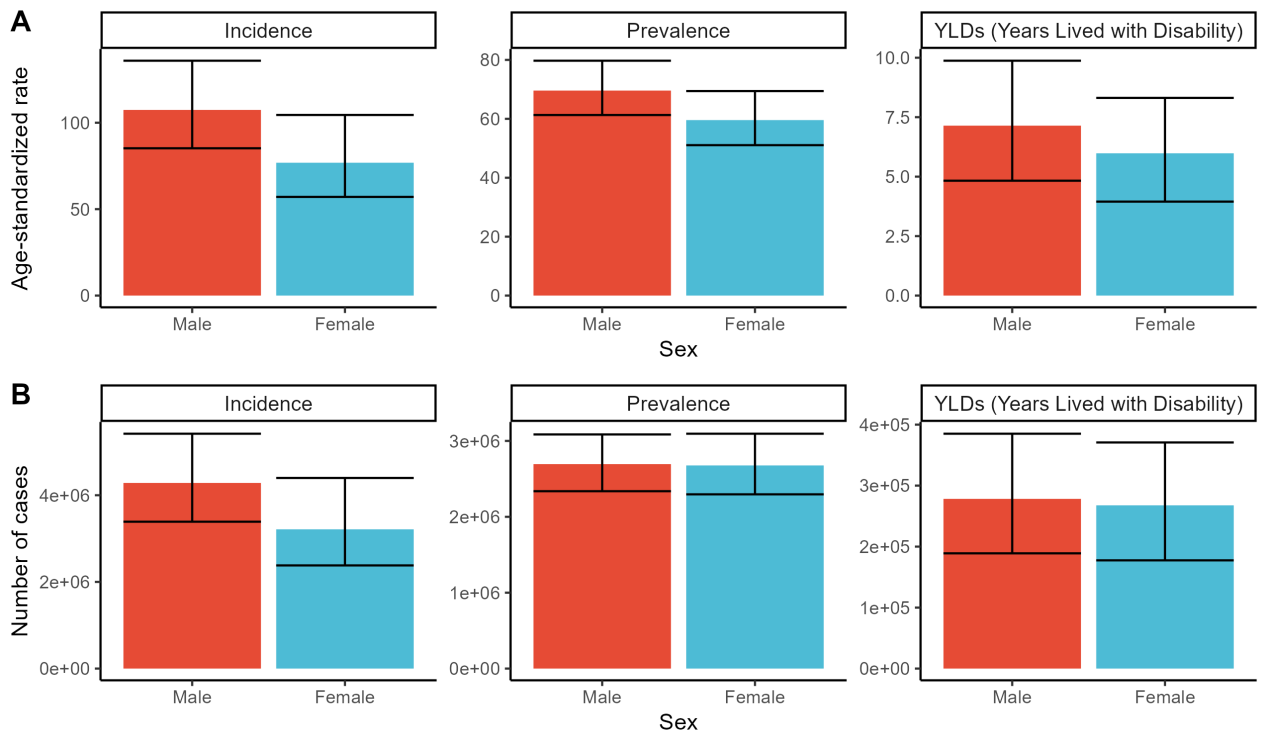


**Figure S2.** Numbers and age-standardized rates of fracture of vertebral column related incidence, prevalence, and YLDs for different age groups in 2021.


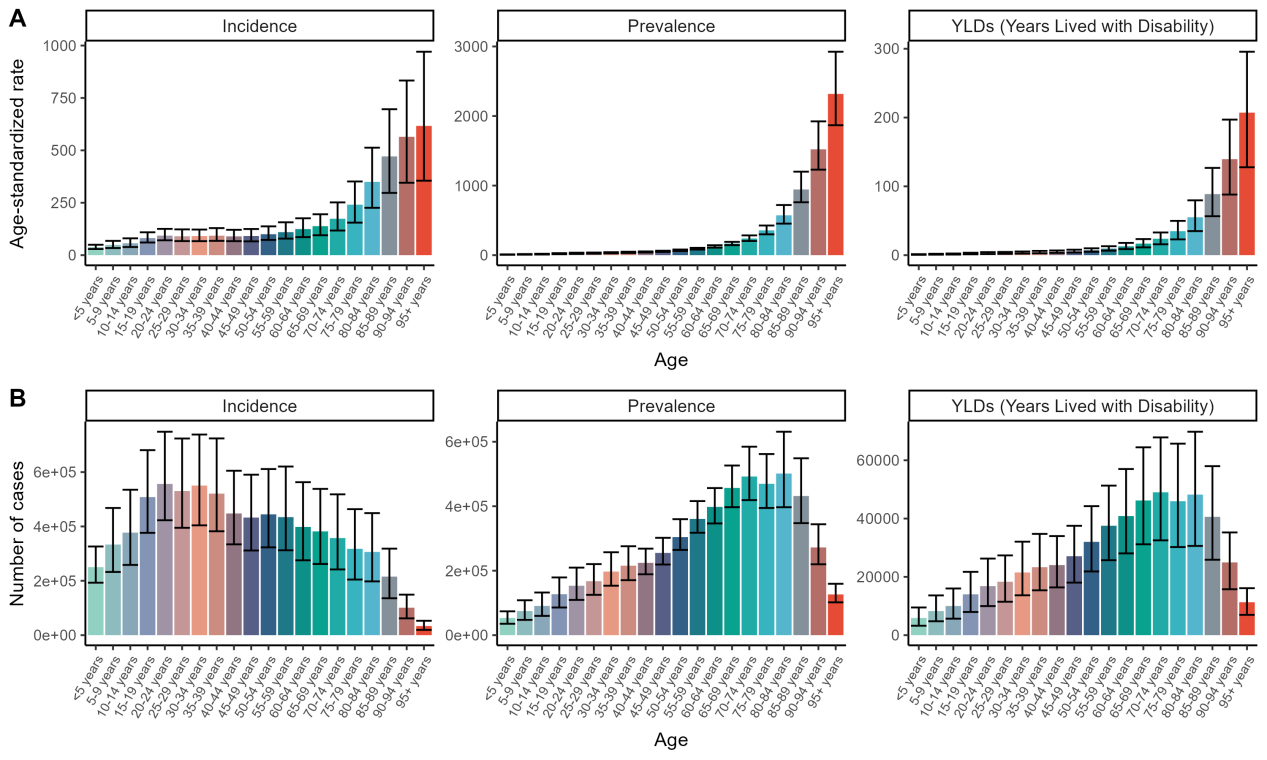


**Figure S3.** Numbers and age-standardized rates of fracture of vertebral column related incidence, prevalence, and YLDs for different SDI region in 2021.

**
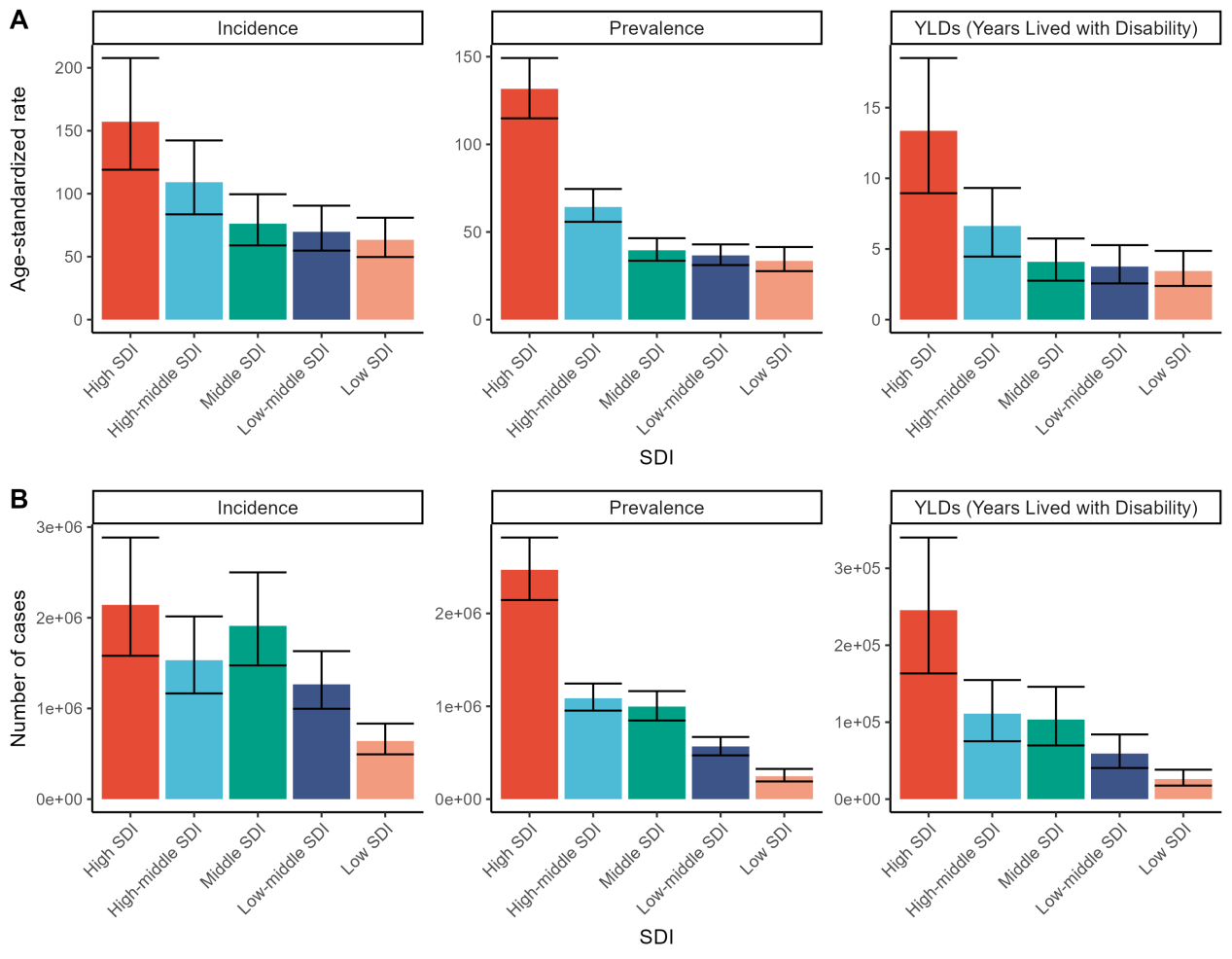
**

**Figure S4.** Numbers and age-standardized rates of fracture of vertebral column related incidence, prevalence, and YLDs for different GBD region in 2021.

**
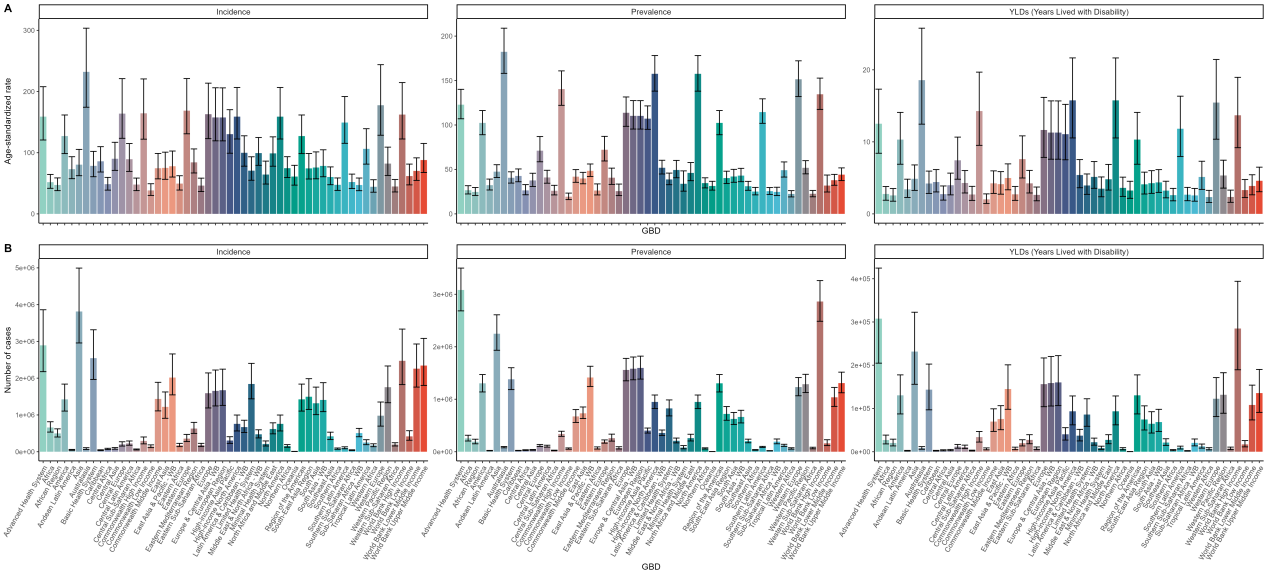
**

**Figure S5.** Trends in the numbers and age-standardized rates of fracture of vertebral column-related incidence, prevalence, and YLDs globally by sex from 1990 to 2021.

**
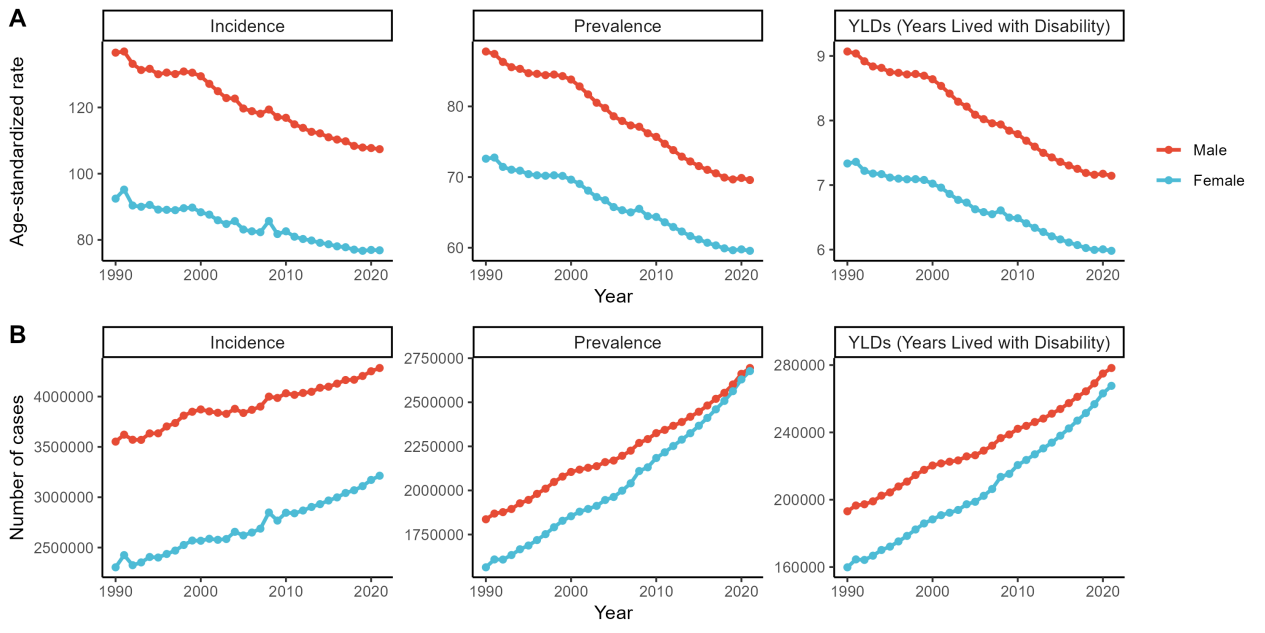
**

**Figure S6.** Trends in the numbers and age-standardized rates of fracture of vertebral column-related incidence, prevalence, and YLDs globally by age groups from 1990 to 2021.

**
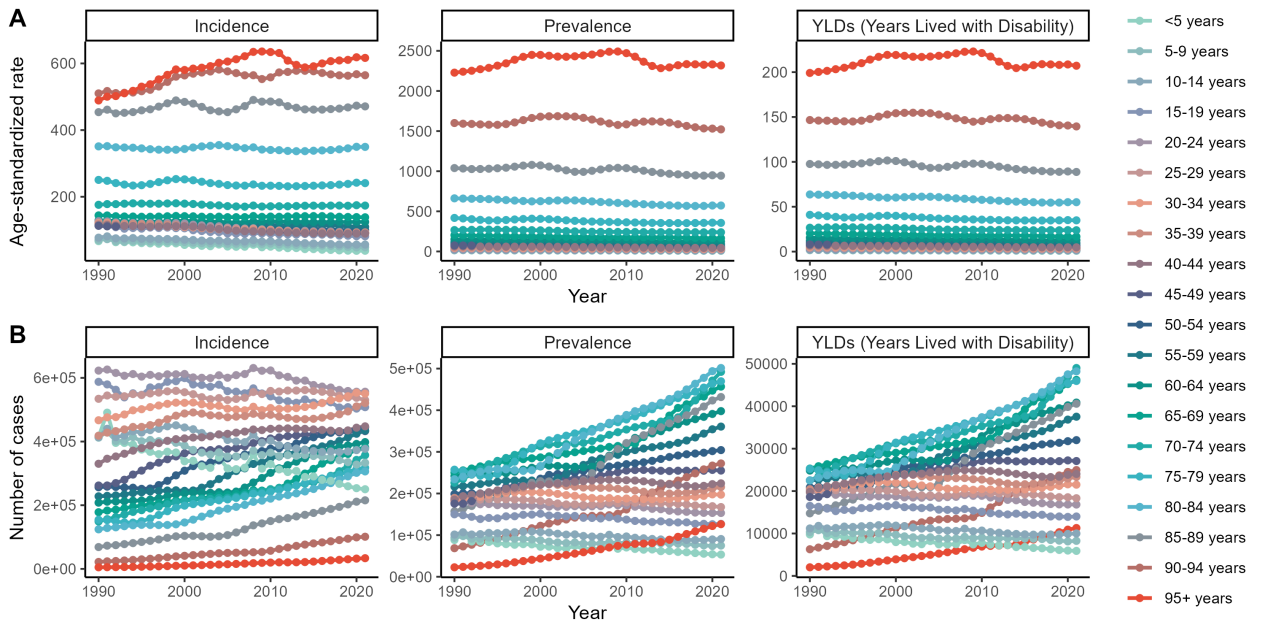
**

**Figure S7.** Trends in the numbers and age-standardized rates of fracture of vertebral column-related incidence, prevalence, and YLDs globally by SDI region from 1990 to 2021.


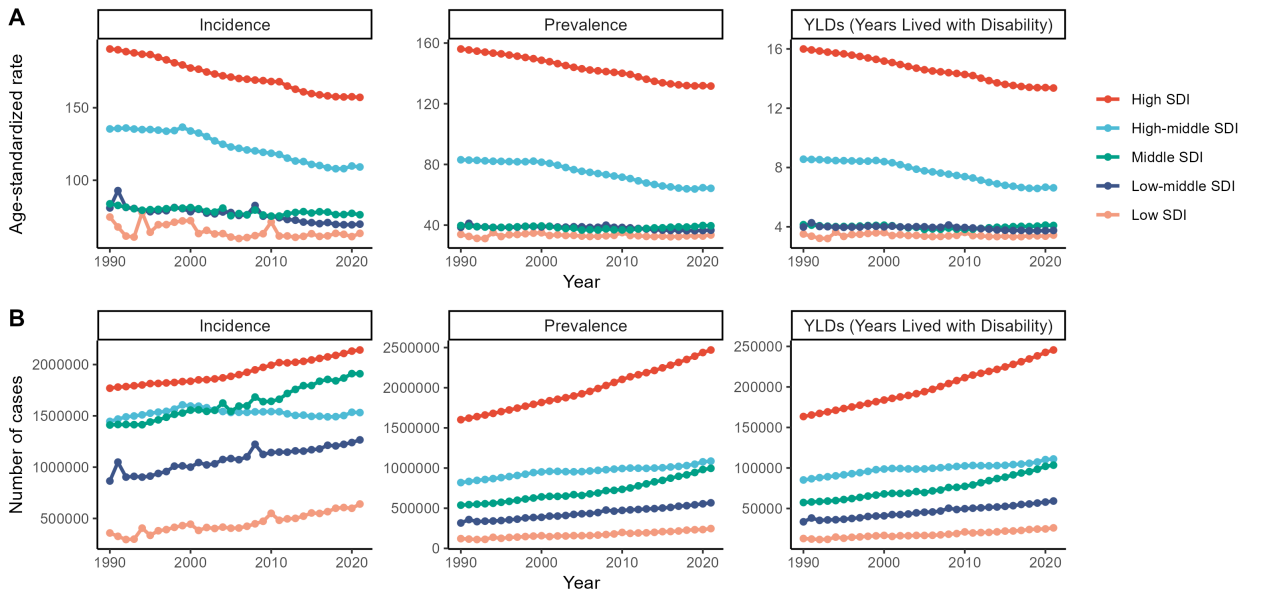

Supplement: Supplementary file 1 [file Data_Sheet_1.docx]
